# 1,4-Benzothiazepines with Cyclopropanol Groups and Their Structural Analogues Exhibit Both RyR2-Stabilizing and SERCA2a-Stimulating Activities

**DOI:** 10.1021/acs.jmedchem.3c01235

**Published:** 2023-11-22

**Authors:** Gyuzel Y. Mitronova, Christine Quentin, Vladimir N. Belov, Jörg W. Wegener, Kamila A. Kiszka, Stephan E. Lehnart

**Affiliations:** † Department of NanoBiophotonics, Max Planck Institute for Multidisciplinary Sciences, Am Fassberg 11, Göttingen 37077, Germany; ‡ Department of Cardiology & Pulmonology, Heart Research Center Göttingen, University Medical Center Göttingen, Robert-Koch-Strasse 42a, Göttingen 37075, Germany; § German Centre for Cardiovascular Research (DZHK), Partner Site Göttingen, Göttingen 37075, Germany

## Abstract

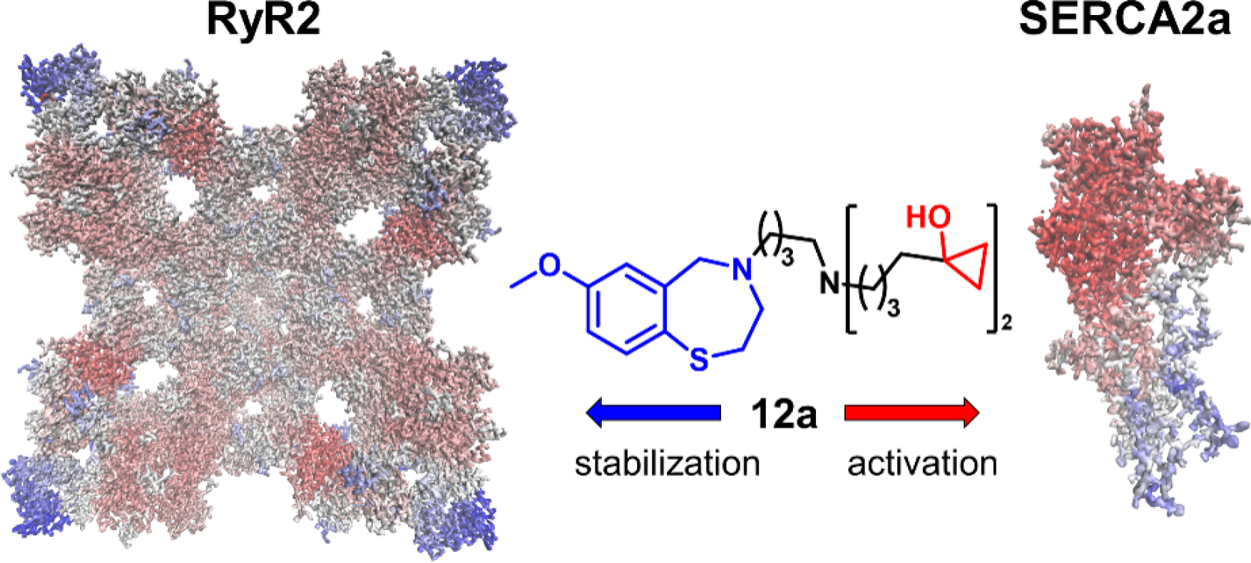

To discover new multifunctional agents for the treatment of cardiovascular diseases, we designed and synthesized a series of compounds with a cyclopropyl alcohol moiety and evaluated them in biochemical assays. Biological screening identified derivatives with dual activity: preventing Ca^2+^ leak through ryanodine receptor 2 (RyR2) and enhancing cardiac sarco-endoplasmic reticulum (SR) Ca^2+^ load by activation of Ca^2+^-dependent ATPase 2a (SERCA2a). The compounds that stabilize RyR2 at micro- and nanomolar concentrations are either structurally related to RyR-stabilizing drugs or Rycals or have structures similar to them. The novel compounds also demonstrate a good ability to increase ATP hydrolysis mediated by SERCA2a activity in cardiac microsomes, e.g., the half-maximal effective concentration (EC_50_) was as low as 383 nM for compound 12a, which is 1,4-benzothiazepine with two cyclopropanol groups. Our findings indicate that these derivatives can be considered as new lead compounds to improve cardiac function in heart failure.

## Introduction

Ryanodine receptor type 2 (RyR2) is an intracellular Ca^2+^ release channel enriched in the sarcoplasmic reticulum (SR) of cardiac muscle cells and in the endoplasmic reticulum (ER) of neurons in the central nervous system.^1−3^ Its main function is to initiate Ca^2+^ release from the SR into the cytoplasm, which activates the contraction of the heart muscle by a process termed calcium-induced Ca^2+^ release.^4,5^ The sarco/endoplasmic reticulum Ca^2+^-ATPase 2a (SERCA2a) is another cardiac Ca^2+^-transport SR protein, a subtype of SERCA transmembrane P-type ATPase, whose primary function is to pump calcium ions back into the SR after they are released by RyR2 during cardiac muscle contraction. This decreases the concentration of cytosolic Ca^2+^ and initiates the diastolic relaxation phase of the myocardium during which a muscle relieves its tension. The Ca^2+^ ions are then stored in the SR, until they are released again by activated RyR2 triggering muscle fiber contraction during the next heartbeat. Abnormal regulation of RyR2 and the reduced activity of SERCA2a lead to impaired cardiac contractility, contributing to cardiovascular diseases, including heart failure (HF) and cardiac rhythm disorders.^2,5−9^ RyR2 channels in the healthy heart remain mostly closed during the relaxation phase of the cardiac cycle.^7^ However, a sustained increased level of Ca^2+^ leak from the SR in the diastolic phase has been reported in heart disease, which has been monitored, for example, in the form of Ca^2+^ sparks representing the spontaneous opening of individual RyR2 clusters.^7^ Under HF conditions, the opening of the receptor is promoted by sustained stress signaling mainly by its hyper-phosphorylation by protein kinase A (PKA) and/or calcium/calmodulin-dependent kinase II (CAMKII), as well as oxidation resulting in chronically increased RyR2 channel activity and increased Ca^2+^ leak from the SR.^2,3,5,10,11^ Increased RyR2 leak leads to Ca^2+^ depletion from the SR reducing cardiac contractility and overactivates sodium–calcium exchanger (NCX) that induces abnormal cardiomyocyte depolarization, promoting cardiac arrhythmias.^12^ Decreased SERCA2a activity, which may occur, for example, because of higher levels of oxidative stress in HF leads to impaired Ca^2+^ replenishment of the SR in cardiomyocytes, resulting in delayed diastolic relaxation.^13−15^

**Figure 1:**
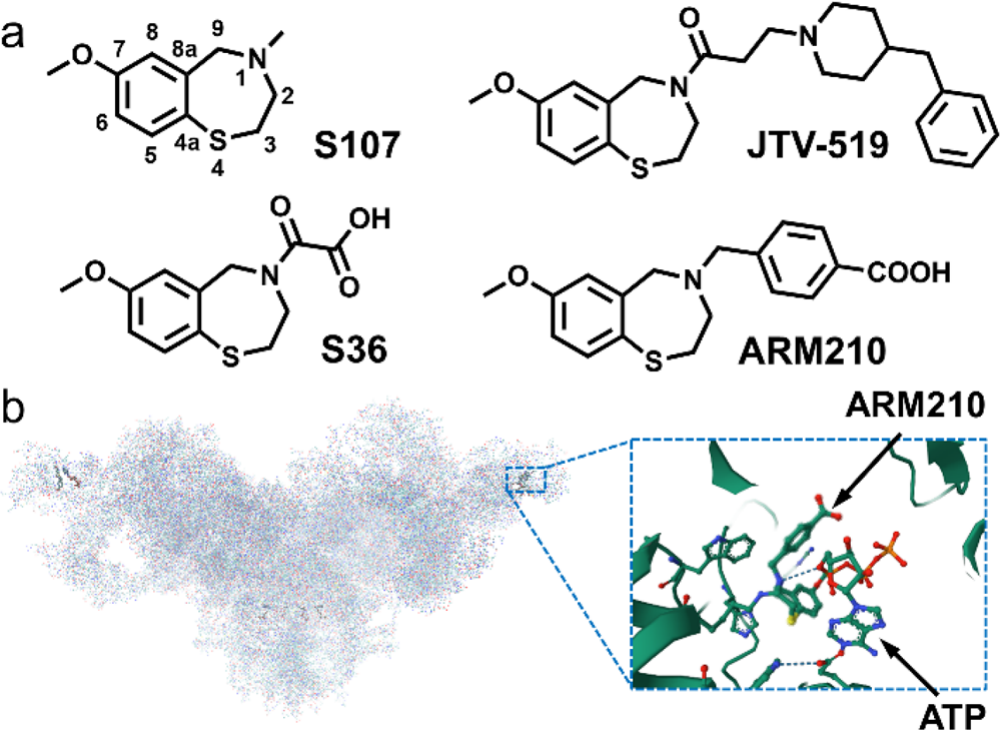
(a) 7-Methoxy-2,3,4,5-tetrahydrobenzo [1,4-*f*]thiazepine derivatives that prevent Ca^2+^ leak from RyR channels. (b) ARM210 in the pocket of human disease-related RyR2-R2474S channel (PDB: 7UA1).^23^

Spontaneous Ca^2+^ leak from the SR through the RyR2 macromolecular complex can be prevented by increasing its affinity to small stabilizing proteins, e.g., the peptidyl-propyl-*cis*-*trans* isomerase calstabin2 (FKBP12.6). Calstabin2 binds to the RyR2 release channel via amphiphilic β-sheet structures reducing the probability of the RyR2 open state during diastole.^16−18^ Restoring calstabin-RyR interaction is a therapeutic strategy that relies on a number of agents belonging to 1,4-benzothiazepines known as Rycals.^2,3,5,16,19,20^ The RyR stabilizers S36, S107, and JTV-519 (Figure 1a) were found to be effective in reducing HF progression and promoting RyR-calstabin binding in both cardiac and skeletal muscle.^17−19^ However, JTV-519 is also a nonspecific blocker of Na^+^, K^+^, and Ca^2+^ channels and acts as a Ca^2+^-dependent SERCA blocker.^17,21,22^ In recent years, there has been considerable interest in ARM210, a second-generation Rycal compound, which binds to disease-associated ryanodine receptor RyR channels not only near the caffeine binding site but also in the RY1&2 domain, thereby stabilizing the RyR closed state by inhibiting the formation of the primed state and thus preventing pathological pore opening (Figure 1a,b).^20,23^

The enhancement of SR calcium load can be achieved by SERCA2a overexpression or by modification of signaling cascades that regulate SERCA2a at multiple levels, for example, via displacement or attenuating the inhibitory effect of the small endogenous protein phospholamban (PLB).^15,24−27^ Recently, Luraghi et al. reported on selective SERCA2a activators, derivatives of istaroxime metabolite, with unknown mechanism of action.^28^ There are published data on the *N*-aryl-*N*-alkyl-thiophene-2-carboxamide compound, which increases endoplasmic reticulum Ca^2+^ load by enhancing SERCA2a-mediated Ca^2+^ transport.^29^ The authors mentioned that the mechanism of action of this compound may involve synergistic effects on both SERCA2a and RyR2.

**Scheme 1:**
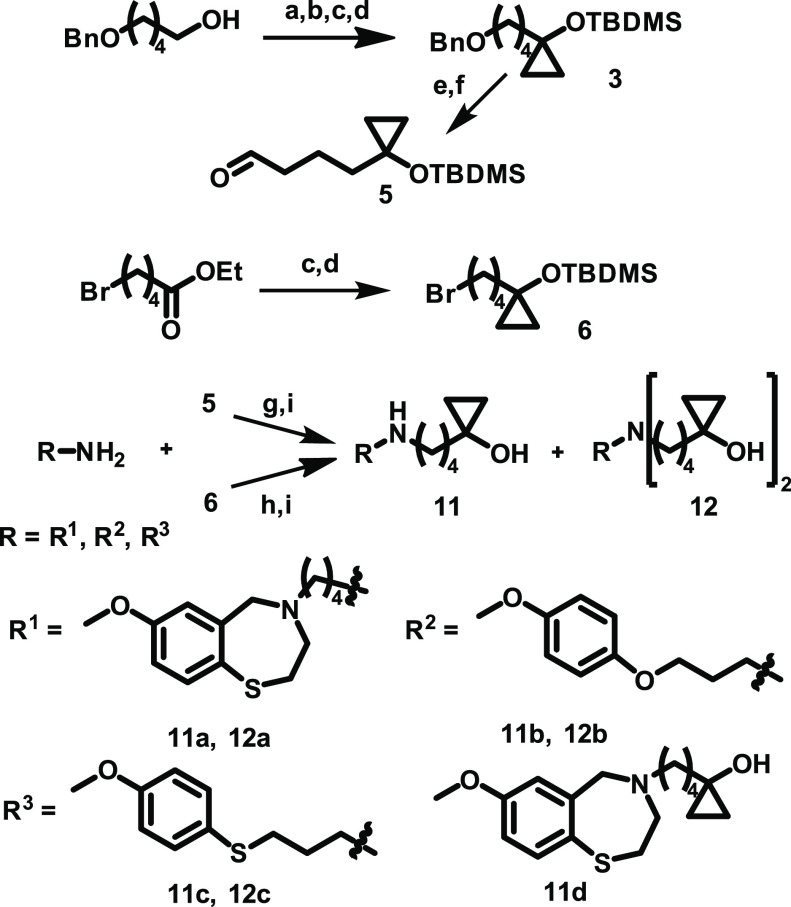
Synthesis of Dual-Acting Compounds with Cyclopropanol Residues Reagents and conditions: (a) Jones reagent, aceton; (b) H_2_SO_4_, EtOH; (c) EtMgBr, Ti(O*i*Pr)_4_,^31^ Et_2_O; (d) *t*-BuMe_2_SiOSO_2_CF_3_, 2,6-lutidine, DCM; (e) H_2_, Pd/C, THF; (f) Dess–Martin reagent, DCM; (g) Na(CH_3_COO)_3_BH, 1,2-dichloroethane; (h) NaH, DMF; (i) aq. HF (55%), MeCN (compounds **11a**, **12a**); 1 M *n*-Bu_4_NF in THF (compounds **11b**,**c** and **12b**,**c**).

In our search for new chemical agents that improve maintenance of diastolic Ca^2+^ levels, we aimed on the development of dual-acting drugs that both inhibit RyR2 Ca^2+^ leak and simultaneously activate Ca^2+^ uptake via SERCA2a. To this end, we developed novel 1,4-benzothiazepines, 3-[(4-methoxyphenyl)oxy]- and 3-[(4-methoxyphenyl)thio]propane-1-amine derivatives containing a cyclopropyl alcohol fragment as a pharmacophore (Scheme 1). The rationale was that the structural similarity between these molecules may result in similar physical properties and biological functions. Indeed, not only 1,4-benzothiazepines but also their “open” analogs, 3-[(4-methoxyphenyl)oxy]-and 3-[(4-methoxyphenyl)thio]propane-1-amine derivatives, reduced Ca^2+^ leak from the ER in our assay on inducible RyR2-expressing HEK-293 cells. In addition, these compounds enhanced ATP hydrolysis mediated by SERCA2a activity in cardiac SR microsomes and increased the SR Ca^2+^ content in HL-1 heart cells in the caffeine-induced Ca^2+^ release assay. Therefore, the presence of cyclopropyl alcohol groups in 1,4-benzothiazepines and their structural analogs results in compounds with dual RyR2-stabilizing and SERCA2a-activating properties.

We report here the synthesis and structure–activity relationship of these compounds, some of which exhibit EC_50_ in the nanomolar range for modulating either RyR2 or SERCA2a activity, suggesting their potential therapeutic application.

## Results

### Chemistry

To prepare the cyclopropyl alcohol derivatives, we used the following major steps: 3-[(4-methoxyphenyl)thio]propan-1-amine was synthesized from 4-methoxybenzenethiol by alkylation with 3-bromopropan-1-amine. The Kulinkovich cyclopropanation of ethyl 5-(benzyloxy)pentanoate and 5-bromopentanoate followed by *tert*-butyldimethylsilyl (TBDMS) protection of the hydroxyl group gave intermediates **3** and **6** (Scheme 1).^30,31^ Compound **11a** was prepared by direct alkylation of 4-(7-methoxy-2,3-dihydrobenzo[1,4-*f*]thiazepin-4(5*H*)-yl)butan-1-amine (**8**) with alkyl bromide **6** (Scheme 1) followed by cleavage of the TBDMS group. Amine **8** was synthesized in two steps from commercially available 7-methoxy-2,3,4,5-tetrahydrobenzo[1,4-*f*]thiazepine and 2-(4-bromobutyl)isoindoline-1,3-dione. Compounds **11b**,**c** and **12c** were obtained from 3-[(4-methoxyphenyl)oxy]propan-1-amine or freshly prepared 3-[(4-methoxyphenyl)thio]propan-1-amine (**15**) using a procedure similar to that of **11a**. Alternatively, we used reductive alkylation of these amines with 4-[1-[(*tert*-butyldimethylsilyl)oxy]cyclopropyl]butanal (**5**) for getting compounds **11d** and **12a**,**b** (Scheme 1). The cleavage of the *tert*-butyldimethylsilyl group was achieved in aq. HF/MeCN mixture, which was then added at 0 °C. Compounds **11a** and **12a** formed rapidly under these conditions after 1 h.

The syntheses of compounds **11b**,**c** and **12b**,**c** were not performed under these acidic conditions, because we expected the rearrangement of cyclopropanols into the corresponding ethyl ketones.^30^ Stirring of TBDMS-protected cyclopropyl alcohols in 1 M tetrabutylammonium fluoride (TBAF) in THF afforded compounds **11b**,**c** and **12b**,**c**. The synthesis of S107 (4-methyl-7-methoxy-2,3,4,5-tetrahydro-1,4-benzothiazepine, Figure 1a) was performed by reductive methylation of 7-methoxy-2,3,4,5-tetrahydrobenzo[1,4-*f*]thiazepine with aqueous formaldehyde.^32^ Compound S36 was synthesized according to the procedure described elsewhere.^19^ Compound ARM210 was obtained from 7-methoxy-2,3,4,5-tetrahydrobenzo[1,4-*f*]thiazepine and 4-(bromomethyl)benzoic acid.

### Characterization of Target Compounds: RyR2 Activity

To assess the functional potency of novel agents for modulating RyR2 channel activity, we measured changes in Ca^2+^ levels in ER of HEK-293 cells with inducible expression of wild-type RyR2 (WT RyR2).^33,34^ Because the expression of WT RyR2 introduces an additional spontaneous Ca^2+^ leak from the ER, these cells are suitable for our purpose of testing potential RyR2 stabilizers.^35^ The cells additionally stably express the Ca^2+^ fluorescence indicator R-CEPIA1*er*, which is a genetically encoded sensor protein of Ca^2+^ in the ER (the cell line was a gift from T. Murayama, Department of Pharmacology, Juntendo University School of Medicine, Tokyo, Japan).^36^

**Figure 2:**
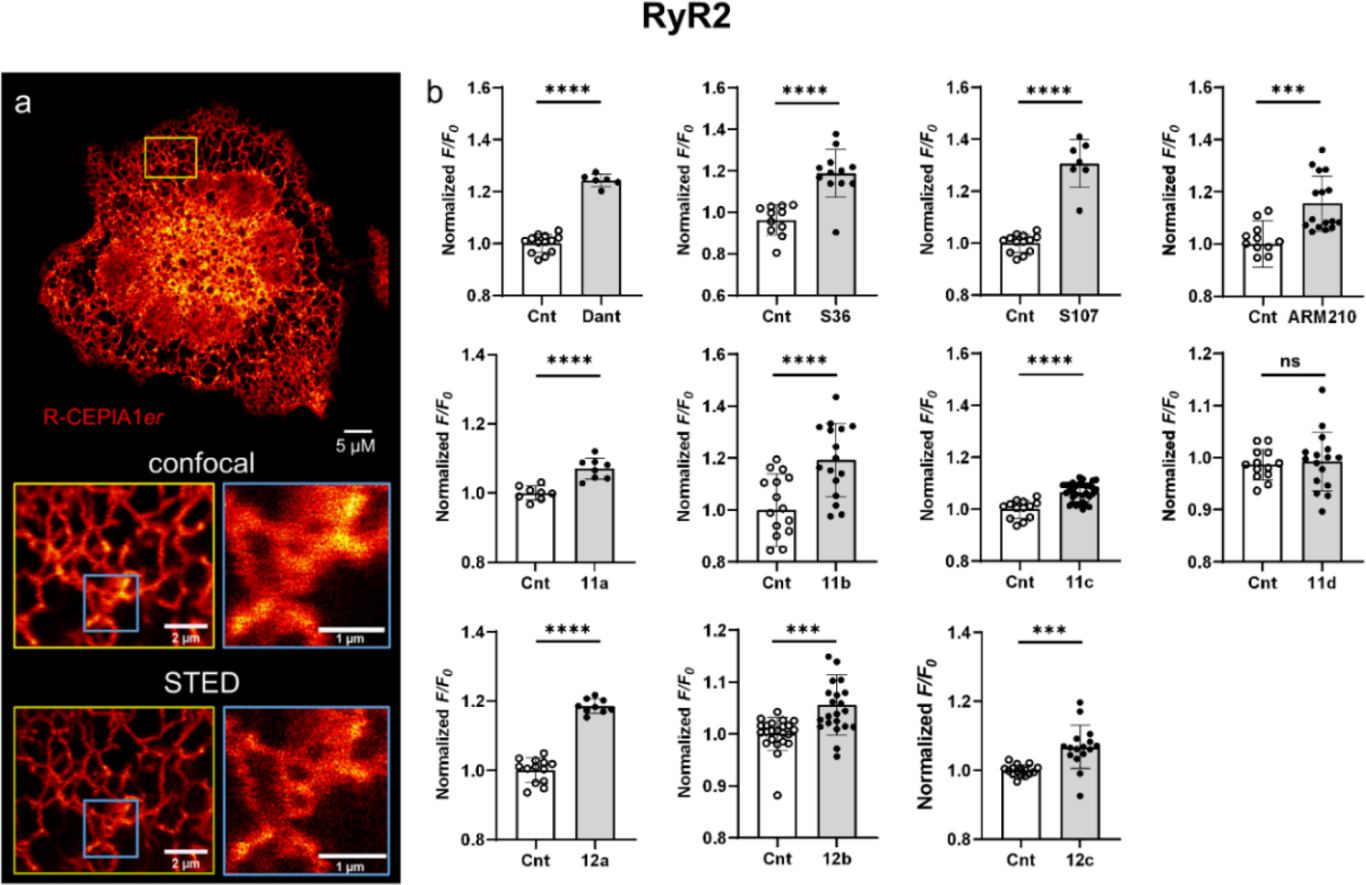
(a) Confocal and STED images (Abberior STED 775 QUAD scan) of live HEK-293 RyR2 R-CEPIA1*er* cells. Scale bar: overview −5 μm; inserts −2 and 1 μm. (b) R-CEPIA1*er* assay fluorescence measurements with HEK-293 cells expressing WT RyR2. *F*/*F*_0_ is the ratio between the average fluorescence before (first 90 s) and after (last 100 s) injection of the test compound. The effect of compound addition (25 μM, 1 mM S107) on [Ca^2+^]_ER_ was normalized to the control (Cnt, 0.1 v/v% DMSO). Dant—dantrolene. Values represent the mean ± S.D., ***P < 0.0005, ****P < 0.0001 vs DMSO by unpaired *t* test; *n* = 6–28.

Fluorescence microscopy of live HEK-293 RyR2 R-CEPIA1*er* cells revealed a highly dynamic branching tubule network of ER structures, indicating the correct position of the calcium sensor in the ER (Figure 2a). Additionally, we used stimulated emission depletion (STED) microscopy and found out that the R-CEPIA1*er* indicator is compatible with superresolution live cell imaging.^37^ In STED microscopy, the stained ER structures appear about three times thinner than in the confocal one (for the line profiles, see Figure S1).

Using time-resolved fluorescence measurements (λ_ex_ = 560 nm, λ_em_ = 610 nm), we observed changes in the concentration of Ca^2+^ in the ER ([Ca^2+^]_ER_) in HEK-293 RyR2 R-CEPIA1*er* cells upon addition of tested compounds that correspond to RyR2 channel activity (Figure S2a).^36^ We used a RyR2 inhibitor dantrolene,^38^ as well as RyR stabilizers S36^8^ and S107^3^ as positive controls to test how their addition would affect the calcium concentration in ER in this model system. The addition of these compounds, which are known to inhibit excessive cardiac RyR2 activity, should reduce Ca^2+^ leak from the ER, thus increasing the R-CEPIA1*er* fluorescence.

For the evaluations of our results, we calculated fluorescence ratio *F*/*F*_0_, where *F*_0_ is the average fluorescence for the first 90 s and *F* is the average fluorescence for the last 100 s, and normalized it to the effect obtained from the control solution (Figure S2a). Indeed, the addition of 25 μM dantrolene or S36 or 1 mM S107 resulted in an increase in R-CEPIA1*er* fluorescence (Figure 2b). ARM210,^23^ which belongs to the second generation of Rycals, significantly increased [Ca^2+^]_ER_, proving its stabilizing activity in HEK-293 RyR2 R-CEPIA1*er* cells (Figure 2b). To avoid false-positive results due to autofluorescence of the tested compounds, we repeated experiments with HEK-293 cells endogenously expressing wild-type RyR2 without the Ca^2+^ indicator (Juntendo University School of Medicine, Tokyo, Japan) and detected no fluorescence in this spectral region (data not shown).

Compounds **11** and **12**, with the exception of **11d**, significantly increased [Ca^2+^]_ER_, in the RyR2 R-CEPIA1*er* assay (Figure 2b). Compounds **11a**–**c** and **12** were tested in a concentration range from 10^–11^ to 10^–4^ M. These compounds increased *F*/*F*_0_ in a dose-dependent manner (Figure S2b–d). At concentrations above 25 μM, compound **12a** demonstrated the most pronounced effect of all of the other compounds. Compounds **11b**,**c** and **12c** were less efficacious than **12a** but showed lower values of half-maximal effective concentration (81.5, 10.2, and 18.6 nM, respectively) (Table 1).

**Table 1:** Properties of 1,4-Benzothiazepines with Cyclopropyl Alcohol Groups and Their Structural Analogues^a^

						SERCA2a^d^		
compound	MW	Log *P*^b^	*P*_e_ 10^–6^ cm/s	viability^c^, %	RyR2 (EC_50_, nM)	HL-1 (EC_50_, μM)	HEK ER (EC_50_, nM)	mouse SR (EC_50_, nM)
S36	267	0.9 ± 0.7	1.0 (low)	94	+++	–	n.d.	n.d.
S107	209	0.8 ± 0.7	1.2 (medium)	98	+++	–	n.d.	n.d.
ARM210	329	3.8 ± 0.6	6.8 (high)	90	++	+ (34.3; 13.1–157.1)	+	++
**11a**	378	3.3 ± 0.5	0.8 (low)	84	++ (8170; 1221–48060)	+ (2.2; 1.0–5.1)	–	+
**11b**	293	2.2 ± 0.3	4.0 (high)	100	+++ (81.5; 28.9–241.4)	++ (8.6; 4.0–20.1)	–	+
**11c**	309	3.3 ± 0.5	6.9 (high)	73	++ (10.2; 0.8–60.3)	++ (7.6; 4.2–14.4)	+	+++
**11d**	307	3.1 ± 0.5	3.6 (high)	95	–	+ (>1000)	+	++
**12a**	490	4.3 ± 0.6	0.6 (low)	80	+++ (2700; 1160–6570)	++ (9.2; 4.1–22.8)	++ (16; 6–39)	+++ (383; 26–985)
**12b**	405	3.0 ± 0.4	4.0 (high)	100	+ (>23,000)	++ (1.5; 0.6–4.4)	+	+
**12c**	421	4.4 ± 0.5	3.6 (high)	90	+ (18.6; 1.0–101.6)	+++ (10.9; 5.27–24.3)	+	+

a S36, S107, and ARM210 are the known inhibitors of RyR-Ca^2+^ leak.

b Partition coefficients were evaluated using ChemSketch, version 2021.1.1, Advanced Chemistry Development, Inc. (ACD/Labs).

c CytoTox-Glo assay in 100 μM solutions after 24 h incubation.

d “+++” a strong effect, comparable to or greater than that of a known compound at a given concentration (25 μM in RyR2 R-CEPIA1*er* assay, 10 μM in caffeine assay and on microsomal membrane vesicles), “++” moderate effect, “+” observable effect, “–” no effect.

Encouraged by these results, we moved on to a caffeine assay to investigate how the new compounds might modulate the SERCA2a activity.

### SERCA2a Activity in HL-1 Cells

It is known that caffeine stimulates the release of calcium from intracellular stores in cells by acting as a RyR activator.^39,40^ The binding site for caffeine is located on the cytosolic side of the protein between the C-terminal and the S2S3 domain.^41^ When caffeine binds to RyR2, it interacts with specific amino acids on the protein, which causes conformational changes in the protein’s structure.^42^ This opens the RyR2 channel pore, enabling the mass release of calcium ions from the SR. We investigated the effect of caffeine-induced Ca^2+^ release in HL-1 cells treated with cytosolic Ca^2+^ indicator FLIPR Calcium 6 (Molecular Devices). The HL-1 cell line is an immortalized mouse cardiomyocyte cell line commonly used to study the effects of various substances on cell function.^43^ If the tested compound modulates SERCA2a activation, it will cause an increase in Ca^2+^ concentration in the SR and the effect of a fixed dose of caffeine on SR Ca^2+^ release will be potentiated by increasing doses of a proposed SERCA activator.^26^ As a positive control, we used CDN1163, a small-molecule SERCA activator, which is at present in a clinical trial due to its potential therapeutic use for certain cardiac conditions.^27^

Indeed, the addition of 10 mM caffeine solution to HL-1 cells incubated for 2 h in CDN1163 solutions of various concentrations together with the cytoplasmic Ca^2+^ indicator showed a clear dependence of the magnitude of the caffeine effect from the concentration of CDN1163 (Figure S3a,b). The half decay time (*T*_1/2_) of caffeine-induced Ca^2+^ release gradually increases from 3 to 50 s with increasing CDN1163 concentration (Figures S3a). We assumed that the decay of cytosolic [Ca^2+^] in the presence of caffeine is an indicator of NCX activity, as postulated by Chen et al.^44^ NCX is a transmembrane transporter in the cytoplasmic membrane that removes one Ca^2+^ ion out of the cell in exchange for three sodium ions.^45^ Such a concentration-dependent change in *T*_1/2_ may indicate that NCX activity slows down as the concentration of CDN1163 increases.

**Figure 3:**
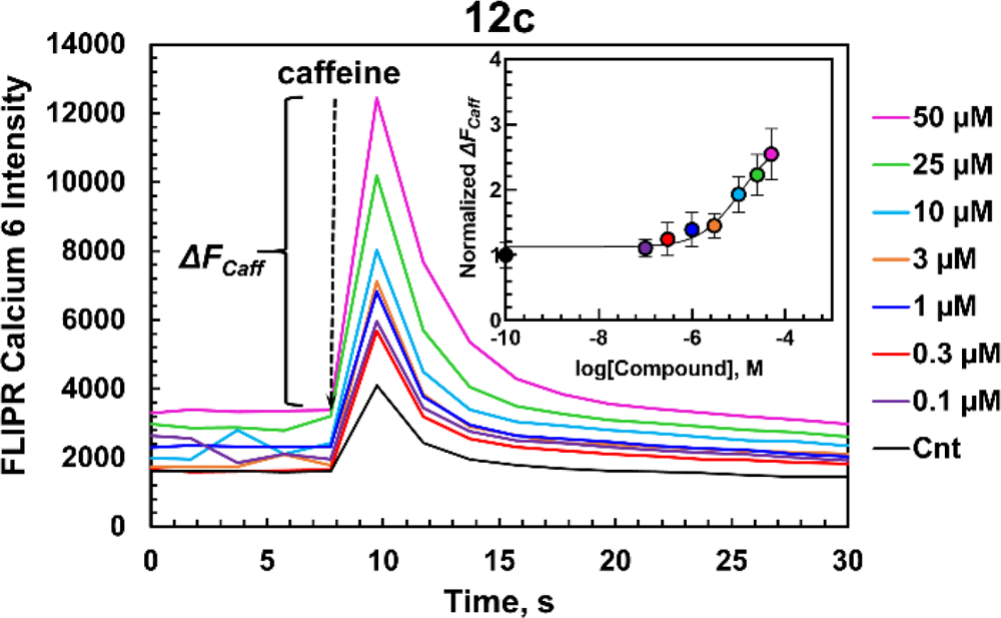
Caffeine-induced Ca^2+^ release assay. HL-1 cells were incubated with the FLIPR Calcium 6 Ca^2+^ indicator and then treated with various concentrations of compound **12c**. Ca^2+^ influx was initiated by the addition of 10 mM caffeine. The differences between the caffeine-induced peak minus basal fluorescence, Δ*F*_Caff_, were taken for the analysis. The inset shows the dose–response curve of **12c**. The response on caffeine addition was normalized to the control (Cnt, 0.1 v/v% DMSO). Data represent as mean ± S.D., *n* = 8 independent measurements.

**Figure 4:**
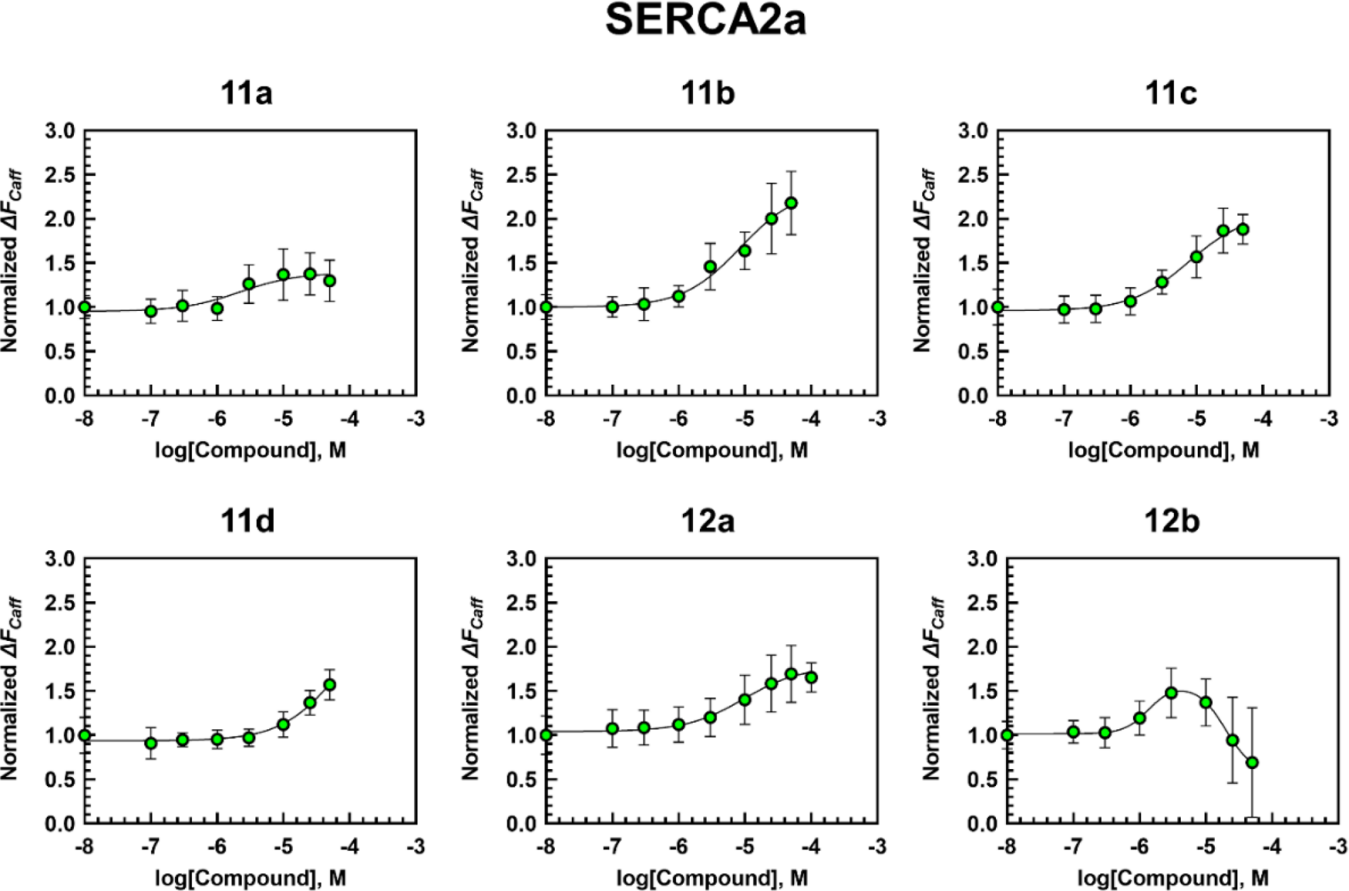
Dose–response curves of compounds **11** and **12a**,**b**. The data were normalized to the 0.1 v/v% DMSO. Values represent the mean ± S.D., *n* = 8–16.

Similarly to CDN1163, compounds **11** and **12** improved SERCA2a activity in the micromolar concentration range, with **11d** being the least efficacious and **12c** being the most efficacious of all other compounds including CDN1163 (Figures 3 and 4). Compound **11d** showed no saturation effect at concentrations up to 50 μM, which did not allow us to calculate its EC_50_ (Figure 4). The EC_50_ values for **11a**–**c** and **12a**,**c** were found to be 2.2, 8.6, 7.6, 9.2, and 10.9 μM, correspondingly (Table 1). Compound **12b** displayed a bell-shaped dose response; it has shown a moderate to good stimulatory effect at 3 and 10 μM (up to 150% compared to the control) that decreased at higher doses, suggesting that the compound may exhibit inhibitory activity at higher concentrations (Figure 4). *T*_1/2_ values demonstrated only a slight difference among samples with various concentrations of tested compounds (from 3 to maximum 5 s when the concentrations changed from 0 to 50 μM), suggesting no significant change of NCX activity within this set of compounds.

**Figure 5:**
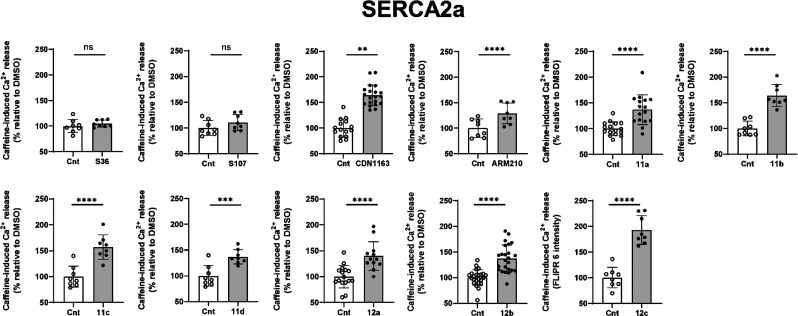
Increase of caffeine-induced Ca^2+^ release by test compounds. Effects of 10 μM solutions of CDN1163, ARM210, S107, **11**, and **12** (25 μM for **11d**) on caffeine-induced Ca^2+^ release. Values represent the mean ± S.D., ^**^P < 0.005, ^***^P < 0.001, ^****^P < 0.0001 vs control (Cnt, 0.1 v/v% DMSO) by unpaired *t* test, *n* = 8–24.

S36 has shown no concentration-dependent effect in the caffeine-induced Ca^2+^ release assay (Figure 5 and Figure S4). This suggests that S36 does not increase SERCA2a activity, which agrees with our previous experiments on wild-type (WT) and disease model cardiomyocytes.^46^ Addition of caffeine to HL-1 cells treated with different concentrations of S107 did not show any effects on SR Ca^2+^ content as well (Figure 5 and Figure S4). Surprisingly, the second-generation Rycal ARM210 drug demonstrated a clear concentration-dependent effect in this assay (Figure S3b). Figure 5 shows the data obtained after the incubation of HL-1 cells in 10 μM (25 μM for **11d**) solutions of compounds.

### SERCA2 Activity in Microsomal Membrane Vesicles

Cyclopropanol derivatives have a variety of biological activities including enzyme inhibition and antibacterial and anticancer properties.^47−50^ However, there have been no published reports on cyclopropanol-containing molecules specifically acting as modulators of RyRs and/or activators of SERCA. To investigate more directly whether the compounds influence the SERCA2 activity, we performed nicotinamide adenine dinucleotide (NADH) fluorescence-coupled ATPase assay according to Radnai et al.^51^ In this assay, the change in NADH coenzyme fluorescence reflects the level of the consumption of ATP, which we took as indicator of SERCA2 activity.^51,52^ ATP hydrolysis by SERCA2 is required to pump calcium ions into the SR. We assume that a higher rate of ATP hydrolysis reflects an increase in SERCA2 ATPase activity that leads to a greater pumping of calcium ions into the SR.

**Figure 6:**
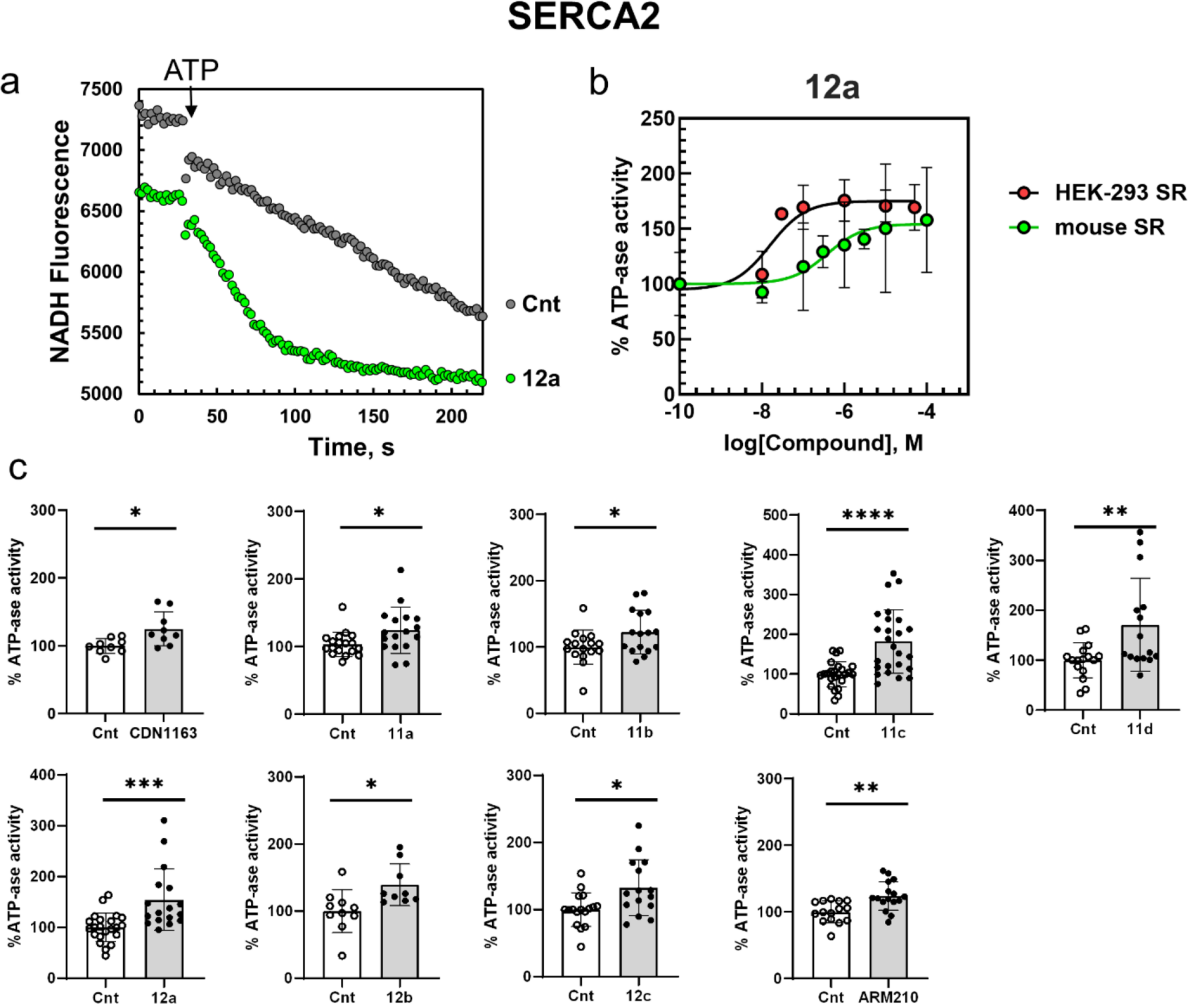
NADH-coupled ATPase assay. (a) Mouse ventricular microsomes were incubated in the assay buffer with and without 100 μM **12a**. After ATP injection, the intrinsic fluorescence of NADH decreases due to ATP consumption. The reaction rate of NADH oxidation is greater in the mouse SR sample containing **12a**. (b) Compound **12a** evokes an enhancement in the kinetic rate of NADH–NAD^+^ conversion in a dose-dependent manner. The effects of compound **12a** are expressed as a percentage of the control effect. Data represented as mean ± S.D., *n* = 3–15 independent measurements. (c) Effect of 10 μM CDN1163, **11**, **12** (3 μM for **12b**), and ARM210 on SERCA2a activity in mouse SR. The data were normalized to the control (Cnt, 0.1 v/v% DMSO). Values represent the mean ± S.D., ^*^P < 0.05, ^**^P < 0.005, ^***^P < 0.001, ^****^P < 0.0001 vs control by unpaired *t* test; *n* = 9–24/3–5 mice.

We observed the rate of NADH oxidation, which is coupled to ATP hydrolysis, by measuring changes in NADH intrinsic fluorescence over time after ATP injection into microsomal membranes enriched in SR vesicles isolated from mouse heart ventricles (mouse SR) and HEK-293T cells (HEK-293 ER). The presence of SERCA2 in mouse SR and HEK-293 ER of isolated microsomal vesicles was confirmed by Western blotting (Figures S5 and S6; for details see Experimental Section). The NADH fluorescence decrease was recorded on a multiwell plate reader TECAN Spark 20M (λ_ex_ = 380 nm, λ_em_ = 470 nm). Figure 6a shows a typical decrease in NADH fluorescence after the injection of 2 mM ATP using mouse heart SR vesicles pretreated with compound **12a**. Consistent with our previous experiment on HL-1 cells, we observed a strong decrease in NADH fluorescence reflecting an increase in SERCA2a activity in SR microsomes derived from mouse heart ventricles (*n* = 3–5) in solutions containing the tested compounds compared to the 0.1 v/v% DMSO sample (Figure 6). In microsomes from HEK-293 ER, the treatment with compounds **11a** and **11b** did not evoke a significant increase in ATPase activity while other compounds, **11c**,**d**, **12a**–**c**, and ARM210, showed significant activation of SERCA2 ATPase (*n* = 3–5) (Figure S7). Similar results were obtained in cardiac SR vesicles from the mouse heart. In this case, all new cyclopropanol compounds, as well as ARM210, caused the improvement of ATPase activity (Figure 6c). For compound **12a**, ATPase activity increased in a concentration-dependent manner with an EC_50_ of 16 and 383 nM for HEK-293 ER and mouse SR, respectively (Table 1, Figure 6b). This difference may indicate isoform-specific effects of our compound in HEK-293 cells expressing mostly SERCA2b^53^ versus mouse SR vesicles containing mostly SERCA2a.^54^ In both experiments, we used CDN1163 as a positive control.

### Drug-Related Cell Toxicity and Membrane Permeability

The cyclopropyl alcohol-substituted compounds showed no significant cell death at concentrations up to 50 μM in CytoTox-Glo Cytotoxicity Assay (Promega GmbH, Figure S8). The membrane permeability of the derivatives was determined using cell-free transport models (PermeaPad, innoME GmbH), and permeability rates (*P*_e_), which are a measure of how fast a compound can cross a membrane, were established (Table 1). Albeit poor permeability shown in the PAMPA assay, compounds S36, **11a**, and **12a** acted on live RyR2-HEK-293 R-CEPIA1*er* cells and the latter two had an effect on HL-1 cells in the caffeine-induced Ca^2+^ release assay.

The properties of the new compounds together with the properties of the known substances S36, S107, and ARM210 are given in Table 1. The values of calculated partition coefficients (log *P*) for cyclopropanol derivatives range from 2.2 to 4.4, which is in line with Lipinski’s “rule of five” claiming that the calculated octanol–water partition coefficient should not exceed 5.^55^

## Discussion and Conclusions

Almost all cyclopropyl alcohols, except for **11d**, which is a 1,4-benzothiazepine derivative with the shortest C_4_H_8_ linker, showed dual RyR2/SERCA2a activity. 1,4-Benzothiazepine **11d** had no significant RyR2-stabilizing effect on HEK-293 RyR2 R-CEPIAer cells but demonstrated slight (HL-1 and HEK ER) and moderate (mouse SR) SERCA2 stimulating activity. The RyR2 tests clearly point out that derivatives of 3-[(4-methoxyphenyl)oxy]- and 3-[(4-methoxyphenyl)thio]propane-1-amine, which are the mimetic analogues of 1,4-benzothiazepine, can activate RyR2 almost to the same extent as known Rycals. Compound **11a**, which is a 1,4-benzothiazepine derivative with one cyclopropanol group and C_4_H_8_NHC_4_H_8_ linker, was most potent in the caffeine-induced Ca^2+^ release assay in HL-1 cells. Although we could not detect SERCA2 activation by this compound (10 μM) on ER vesicles derived from HEK-293T cells, we observed a significant effect on mouse heart microsomes (Figure 6c).

Under certain experimental conditions, compounds **11b**,**c** and **12** demonstrated activities comparable to or superior to those of the known SERCA activator CDN1163. Compound **11c**, which is the 3-[(4-methoxyphenyl)thio]propane-1-amine derivative with one cyclopropanol group, demonstrated nanomolar EC_50_ in the RyR2 assay. The 1,4-benzothiazepine analog **12a**, which contains two cyclopropanol groups and the C_4_H_8_NH(C_4_H_8_)_2_ linker, demonstrated RyR2 stabilizing activity in micromolar concentrations and a nanomolar EC_50_ on HEK-293 microsomes and mouse SR in the NADH assay. Of the other two compounds, 3-[(4-methoxyphenyl)oxy]- and 3-[(4-methoxyphenyl)thio]propane-1-amine derivatives with the same NH(C_4_H_8_)_2_ linker and two cyclopropanol groups (**12b** and **12c**), compound **12c** had a stronger activating effect on caffeine-induced Ca^2+^ release at concentrations above 10 μM, whereas **12b** showed a slight increase in SERCA2a-stimulating activity up to 10 μM and a presumable inhibitory effect at higher concentrations, 25 and 50 μM (Figure 4). Results arising from physiological target validation of this group of compounds will be published in a separate manuscript.

The 1,4-benzothiazepine derivative ARM210, despite its known RyR2-modulating activity, was found to act as a SERCA2a activator. According to single-particle cryo-electron microscopy data, this compound modulates PKA-phosphorylated RyR1 and RyR2, binding to the secondary adenosine triphosphate (ATP) site in the RY1&2 domain.^20,23^ The authors proposed that the similarity of the ATP and ARM210 structures allows this Rycal to occupy the ATP site. It would be interesting for further studies to determine whether ARM210 may also interact with the ATP site of SERCA2a.

**Figure 7:**
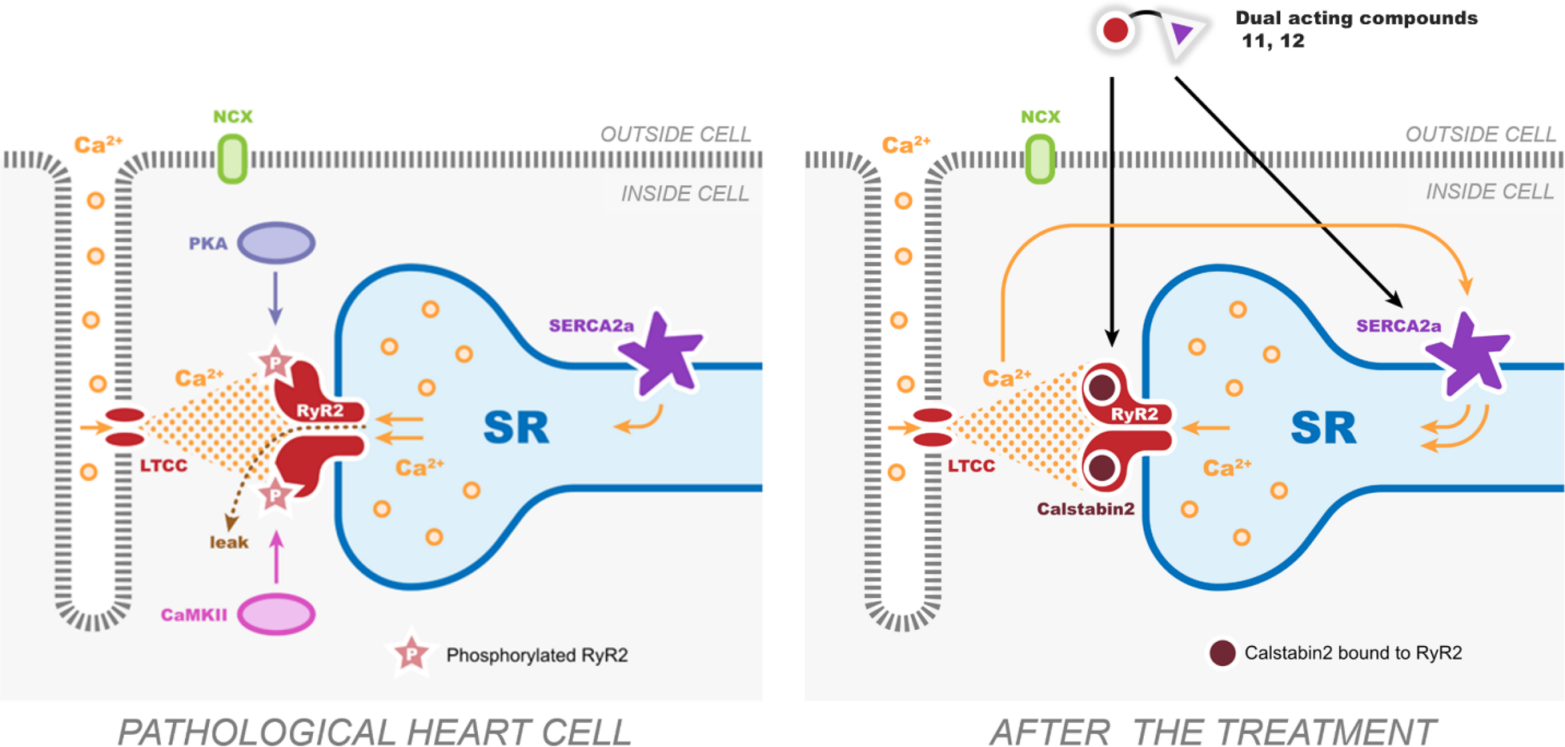
Schematic representation of the modulating effect of cyclopropanol compounds **11** and **12** (modified from Niggli et al.^56^). LTCC = L-type calcium channel; NCX = sodium–calcium exchanger that removes Ca^2+^ from cells. Phosphorylated RyR2 is depicted by added beige stars with a “P” sign. The binding of Calstabin2 (FKBP12.6) to RyR2 is shown by brown circles.

Figure 7 shows a schematic representation of the intracellular mechanism of dual RyR2-SERCA2a action of the novel compounds. The entry of a small amount of Ca^2+^ through the L-type Ca channel (LTCC) activates RyR2 and initiates Ca^2+^ release from the SR, a process called Ca^2+^-induced Ca^2+^-release or CICR, which leads to cardiomyocyte contraction.^56^ During the relaxation, Ca^2+^ is removed from the cytoplasm and pumped back into the SR by SERCA2a. This requires that the RyR channels remain closed. In the heart cells at the pathological stage, one or both proteins might be dysfunctional, leading to abnormal distribution of intracellular Ca^2+^. PKA and CAMKII can phosphorylate RyR2 increasing its sensitivity toward activating Ca^2+^ leading to hyper-active RyR2 that decrease SR Ca^2+^ load due to Ca^2+^ leak, which is not compensated by the reduced Ca^2+^ uptake. Diminished SR Ca^2+^ load is involved in impaired contractility and relaxation observed in HF.

The new cyclopropyl alcohol containing 1,4-benzothiazepines and their structural analogs act on RyR2 stabilizing its closed state while they activate SERCA2a, increasing SR Ca^2+^ load. We showed that the relatively small changes in the structure of the new compounds lead to different biochemical properties with respect to RyR2 and SERCA2a. Both proteins, RyR2 and SERCA2a, are key players in cardiac Ca^2+^ cycling. Therefore, the ability to obtain drugs that can be flexibly adjusted to individual needs (for example, with stronger RyR2 stabilization and moderate SERCA2a activation, or vice versa) could be used in future studies as to achieve dual-acting tools for personalized treatment, improving the contractility of heart muscle to prevent or slow HF and to prevent Ca^2+^ triggered heart rhythm disorders on the individual level.

## Experimental Section

### Chemistry

#### General Materials and Methods

All reagents and solvents were purchased from commercial sources and used without further purification. Flash chromatography was performed using a Biotage Isolera One Flash purification system with a cartridge and solvent gradient indicated. NMR spectra were recorded at ambient temperature on an Agilent 400-MR spectrometer (MPI NAT Göttingen) at 400.06 MHz (^1^H) and 100.60 MHz (^13^C), and the chemical shifts are reported in ppm. All ^1^H spectra are referenced to tetramethylsilane (δ = 0 ppm) by using the signals of the residual protons of CHCl_3_ (7.26 ppm) in CDCl_3_, CHD_2_CN (1.94 ppm) in CD_3_CN, CHD_2_OD (3.31 ppm) in CD3OD, or DMSO-*d*_*5*_ (2.50 ppm) in DMSO-*d*_*6*_. ^13^C spectra are referenced to tetramethylsilane (δ = 0 ppm) using the signals of the solvent: CDCl_3_ (77.16 ppm), CD_3_CN (1.32 ppm), CD_3_OD (49.00 ppm), or DMSO-*d*_*6*_ (39.52 ppm). Multiplicities of signals are described as follows: s = singlet, d = doublet, q = quartet, dq = doublet of quartets, and m = multiplet or overlap of signals. Low-resolution mass spectra (50–3500 *m*/*z*) with electrospray ionization (ESI) were recorded on a Varian 500-MS spectrometer (Agilent) at MPI NAT Göttingen. ^1^H and ^13^C NMR spectra are available in the Supporting Information (Figures S9–S26). High-resolution mass spectra (ESI-HRMS) were recorded on a microTOF spectrometer (Bruker) equipped with an ESI ion source (Apollo) and direct injector with an Agilent RR 1200 LC autosampler at the Institute of Organic and Biomolecular Chemistry (Georg-August-Universität Göttingen).

Liquid chromatography: analytical HPLC was performed with a Knauer AZURA liquid chromatography system. Analytical column: Knauer Eurospher II 100-5, C18–H, 5 μm, 150 × 4.6 mm (unless otherwise stated); solvent A: H_2_O + 0.1% v/v TFA, solvent B: MeCN + 0.1% v/v TFA; temperature 25 °C. All final compounds are >95% pure by HPLC analysis (HPLC peak area >95%, Figures S27–S34). The purity of the compounds was additionally confirmed by the LC-MS method (Figures S35–S40). UltiMate 3000 Standard (SD) HPLC Systems with an ISQ EM Single Quadrupole Mass-Spectrometer were employed. Column: Phenomenex (2.6 μm; 3.0 × 75 mm). Flow rate: 0.5 mL/min. UV detection wavelength: 254 nm. Solvent A: H_2_O + 0.1% formic acid, solvent B: MeCN + 0.1% formic acid; temperature 25 °C. Analytical TLC was performed on MERCK ready-to-use plates with silica gel 60 (F_254_).

Compound S36 was obtained in two steps from 7-methoxy-2,3,4,5-tetrahydrobenzo[1,4-*f*]thiazepine (BLD Pharm, Kaiserslautern, Germany) as described by Marks et al.^19^ Compound S107 was synthesized by methylation of 7-methoxy-2,3,4,5-tetrahydrobenzo[1,4-*f*]thiazepine with aqueous formaldehyde, as described by Smith et al.^32^

ARM210 was obtained from 7-methoxy-2,3,4,5-tetrahydrobenzo[1,4-*f*]thiazepine (20 mg, 0.1 mmol) and 43 mg of 4-(bromomethyl)benzoic acid (0.2 mmol) with 20 μL (0.26 mmol) of pyridine in 3 mL of DCM. The reaction mixture was stirred overnight at RT. After the solvent was evaporated, 2 mL of water was added and the aqueous solution extracted with EtOAc (2 × 5 mL). The combined organic solutions were dried over Na_2_SO_4_. The filtrate was evaporated, and the product was isolated by flash column chromatography using a SNAP Ultra 10 g cartridge with SiO_2_ (gradient: MeOH in DCM 8% – 100%). Yield −9 mg (27%) of yellowish oil. ^1^H NMR (400 MHz, CD_3_OD) δ 8.19–8.10 (m, 2H, H_Ar_), 7.69–7.62 (m, 2H, H_Ar_), 7.57 (d, *J* = 9.2 Hz, 1H, H_Ar_), 7.04–6.96 (m, 2H, H_Ar_), 4.73 (s, 2H, CH_2_), 4.51 (s, 2H, CH_2_), 3.82 (s, 3H, CH_3_), 3.69–3.59 (m, 2H, CH_2_), 3.16–3.06 (m, 1H, CH_2_). ESI-MS, negative mode: *m*/*z* (rel. int., %) = 328 (100) [*M*-H]^−^. HRMS *m*/*z* calcd for C_18_H_18_NO_3_S [M - H]^−^, 328.1013; found, 328.1017.

Ethyl 5-(benzyloxy)pentanoate (**1**) was obtained in two steps from 5-(benzyloxy)pentan-1-ol (BLD Pharm, 5g, 26 mmol) as described by Shi et al.^57^

1-(4-(Benzyloxy)butyl)cyclopropan-1-ol (**2**) was prepared from compound **1** by the Kulinkovich reaction.^31^ To a mixture of Ti(O^*i*^Pr)_4_ (279 μL, 265 mg, 0.93 mmol) and ethyl 5-(benzyloxy)pentanoate (2.6 g, 11 mmol) in 3 mL Et_2_O, a solution of EtMgBr in diethyl ether (3 M, 8 mL, 24 mmol) was added over 1 h at 0 °C. The reaction mixture was stirred for 5 h at 0 °C, hydrolyzed by addition of cold 10% H_2_SO_4_ solution in water (20 mL), and then extracted with EtOAc (3 × 10 mL). The combined organic solutions were washed with saturated aq. NaHCO_3_ and brine and dried over Na_2_SO_4_. The product was used as obtained without further purification. Yield −2.4 g (99%) of yellowish oil. ^1^H NMR (400 MHz, CDCl_3_) δ 7.34 (s, 5H, H_Ar_), 4.51 (s, 2H, OCH_2_), 3.50 (s, 2H, OCH_2_), 2.49–2.37 (m, 2H, CH_2_), 1.71–1.51 (m, 5H, OH, CH_2_), 0.73 (s, 2H, CH_2_), 0.43 (s, 2H, CH_2_). ^13^C NMR (101 MHz, CDCl_3_) δ: 138.5, 128.4, 128.3, 127.7, 127.6, 127.5, 72.9, 70.4, 70.0, 42.0, 38.0, 29.6, 22.6, 13.5, 13.5. ESI-MS, positive mode: *m*/*z* (rel. int., %) = 221 (100) [M + H]^+^.

(1-(4-(Benzyloxy)butyl)cyclopropoxy)(*tert*-butyl)dimethylsilane (**3**) was obtained from compound **2** (1.2 g, 5.4 mmol), *tert*-butyldimethylsilyl trifluoromethanesulfonate (Sigma-Aldrich, 2.2 g, 8.2 mmol), and 2,6-lutidine (Acros Organics, 1.4 g, 13.1 mmol) in 10 mL of DCM.^58^ The reaction mixture was stirred at 0 °C for 2 h, poured onto ice (30 mL) and saturated aq. NaHCO_3_ (30 mL), and extracted with DCM. The combined organic solutions were dried over Na_2_SO_4_. The filtrate was evaporated, and the product was isolated by flash column chromatography using a SNAP Ultra 25 g cartridge with SiO_2_ (gradient: DCM in hexane 8–100%). Yield −1.7 g (94%) of clear oil. The compound was used in the next reaction without further purification. ^1^H NMR (400 MHz, CDCl_3_) δ 7.35 (s, 2H, H_Ar_), 7.34–7.33 (m, 2H, H_Ar_), 7.31–7.27 (m, 1H, H_Ar_), 4.51 (s, 2H, CH_2_), 3.50 to – 3.45 (m, 2H, CH_2_), 1.70–1.34 (m, 6H, CH_2_), 0.85 (d, *J* = 0.5 Hz, 9H, CH_3_), 0.74–0.62 (m, 2H, CH_2_), 0.45–0.32 (m, 2H, CH_2_), 0.09 (s, 6H, SiCH_3_). ^13^C NMR (101 MHz, CDCl_3_) δ: 138.8, 128.5, 127.7, 127.6, 73.0, 70.7, 56.9, 39.1, 30.0, 25.9, 22.8, 17.9, 13.2, −3.3. ESI-MS, positive mode: *m*/*z* (rel. int., %) = 335 (100) [M + H]^+^.

##### 4-(1-((*tert*-Butyldimethylsilyl)oxy)cyclopropyl)butan-1-ol (**4**)

A 50 mL Schlenk flask was evacuated and flushed with argon two times. Pd/C (Merck, 89 mg; oxidized form) and THF (4 mL) were added, and the mixture was stirred vigorously under hydrogen to activate the catalyst. A solution of **3** (200 mg, 0.60 mmol) in 2 mL of THF was then added. The reaction mixture was stirred for 30 min at room temperature under H_2_. Hydrogen was replaced with argon, and the mixture was filtered through Celite. The filter cake was washed with MeOH. The solvents were evaporated *in vacuo*. The title compound was isolated by flash chromatography using Biotage HP-Sfär Silica HC Duo 20 μm, 25 g (gradient: DCM in hexane 8–100%). Yield −90 mg (62%) of clear oil. ^1^H NMR (400 MHz, CDCl_3_) δ 3.68–3.60 (m, 2H, CH_2_), 1.66–1.38 (m, 2H, 6H, CH_2_), 0.84 (s, 9H, CH_3_), 0.71–0.64 (m, 2H, CH_2_), 0.44–0.38 (m, 2H, CH_2_), 0.08 (s, 6H, CH_3_). ^13^C NMR (101 MHz, CDCl_3_) δ: 63.0, 56.7, 38.9, 32.8, 25.7, 22.2, 17.8, 13.0, −3.5. ESI-MS, positive mode: *m*/*z* (rel. int., %) = 245 (100) [M + H]^+^. HRMS *m*/*z* calcd for C_13_H_28_NaO_2_Si [M + Na]^+^, 267.1751; found, 267.1754.

4-(1-((*tert*-Butyldimethylsilyl)oxy)cyclopropyl)butanal (**5**) was prepared from alcohol **4** (512 mg, 2.1 mol) and Dess–Martin periodinane (TCI, 1.15 g, 2.7 mmol) in 5 mL of DCM by the method of Dess and Martin.^59^ Yield −467 mg (92%) of yellowish oil. ^1^H NMR (400 MHz, CD_3_CN) δ 9.69 (s, 1H, CH), 2.46–2.38 (m, 2H, CH_2_), 1.84–1.73 (m, 2H, CH_2_), 1.56–1.47 (m, 2H, CH_2_), 0.85 (s, 9H, CH_3_), 0.67 (d, *J* = 5.1 Hz, 2H, CH_2_), 0.47–0.37 (m, 2H, CH_2_), 0.10 (s, 6H, CH_3_). ^13^C NMR (101 MHz, CD_3_CN) δ 204.0 (CO), 118.3, 67.7, 57.4, 44.1, 39.0, 26.1, 19.6, 13.5, −3.2. ESI-MS, positive mode: *m*/*z* (rel. int., %) = 242 (100) [M]^+^. HRMS *m*/*z* calcd for C_18_H_26_NaO_2_Si [M + Na]^+^, 265.1594; found, 265.1596.

(1-(4-Bromobutyl)cyclopropoxy)(*tert*-butyl)dimethylsilane (**6**) was obtained from ethyl 5-bromopentanoate (BLD Pharm) using the same procedure as described by Elek et al.^60^

##### 2-(4-(7-Methoxy-2,3-dihydrobenzo [1,4- *f*]thiazepin-4(5*H*)-yl)butyl)isoindoline-1,3-dione (**7**)

2-(4-Bromobutyl)isoindoline-1,3-dione (BLD Pharm, 700 mg, 2.5 mmol) and K_2_CO_3_ (Merk, 350 mg, 2.5 mmol) were added to a solution of 7-methoxy-2,3,4,5-tetrahydrobenzo[1,4-*f*]thiazepine (530 mg, 2.7 mmol) in anhydrous 1,4-dioxane (20 mL). Then, the reaction mixture was refluxed overnight under an argon atmosphere. After the reaction mixture was filtered from the precipitate, 10 mL of DCM and 10 mL of water were added to the filtrate. The organic phase was separated, and the aqueous layer was extracted with DCM (2 × 3 mL). The combined organic solutions were dried over Na_2_SO_4_. The filtrate was evaporated, and the product was isolated by flash chromatography using a Biotage SNAP Ultra 25 g cartridge with SiO_2_ (gradient: ethyl acetate in hexane 20–100%). Yield −683 mg (69%) of yellowish oil. ^1^H NMR (400 MHz, CDCl_3_) δ 7.85–7.78 (m, 2H, H_Ar_), 7.73–7.66 (m, 2H, H_Ar_), 7.42 (d, *J* = 8.4 Hz, 1H, H_Ar_-5), 6.78 (d, *J* = 2.8 Hz, 1H, H_Ar_-8), 6.66 (dd, *J* = 8.4, 2.8 Hz, 1H, H_Ar_-6), 4.09 (s, 2H, NCH_2_), 3.77 (s, 3H, OCH_3_), 3.68 (t, *J* = 7.1 Hz, 2H, CH_2_), 3.33–3.26 (m, 2H, CH_2_), 2.67 (dd, *J* = 6.6, 3.3 Hz, 2H, CH_2_), 2.45–2.35 (m, 2H, CH_2_), 1.73–1.63 (m, 2H, CH_2_), 1.57–1.48 (m, 2H, CH_2_). ^13^C NMR (101 MHz, CDCl_3_) δ: 168.5, 159.2, 144.7, 134.1, 134.0, 133.7, 132.2, 127.8, 123.4, 123.3, 116.9, 112.4, 59.6, 58.3, 55.4, 51.4, 37.9, 30.2, 26.4, 24.7. ESI-MS, positive mode: *m*/*z* (rel. int., %) = 397 (100) [M + H]^+^. HRMS *m*/*z* calcd for C_22_H_25_N_2_O_3_S [M + H]^+^, 397.1580; found, 397.1583.

##### 4-(7-Methoxy-2,3-dihydrobenzo[1,4-*f*]thiazepin-4(5*H*)-yl)butan-1-amine (**8**)

The mixture of compound **7** (230 mg, 0.58 mmol) and hydrazine hydrate (ABCR, 65 mg, 1.3 mmol) in 2 mL of ethanol was stirred at 60 °C for 30 min. Then, 3 mL of water was added and the aqueous solution was extracted with DCM (2 × 3 mL). The combined organic solutions were dried over Na_2_SO_4_. The filtrate was evaporated, and the product was isolated by preparative HPLC with gradient elution (B/A, 20/80 → 100/0). Yield −144 mg (93%) of yellowish oil. ^1^H NMR (400 MHz, CDCl_3_) δ 7.42 (d, *J* = 8.4 Hz, 1H, H_Ar_-5), 6.97 (d, *J* = 2.8 Hz, 1H, H_Ar_-8), 6.72 (dd, *J* = 8.5, 2.8 Hz, 1H, H_Ar_-6), 4.27 (s, 2H, NCH_2_), 3.79 (s, 3H, OCH_3_), 3.48–3.40 (m, 2H, CH_2_), 3.08 (t, *J* = 6.1 Hz, 2H, CH_2_), 2.78 (dd, *J* = 6.6, 3.2 Hz, 2H, CH_2_), 2.58 (t, *J* = 6.1 Hz, 2H, CH_2_), 1.94 (p, *J* = 6.2 Hz, 2H, CH_2_), 1.77 (q, *J* = 6.3 Hz, 2H, CH_2_). ^13^C NMR (101 MHz, CDCl_3_) δ: 159.2, 144.8, 133.7, 127.9, 117.1, 112.1, 77.5, 77.4, 77.2, 76.8, 59.6, 58.4, 55.5, 51.9, 42.1, 31.2, 30.2, 28.1, 25.0, 24.8. ESI-MS, positive mode: *m*/*z* (rel. int., %) = 267 (100) [M + H]^+^. HRMS *m*/*z* calcd for C_14_H_23_N_2_OS [M + H]^+^, 267.1526; found, 267.1527.

##### 4-(1-((*tert*-Butyldimethylsilyl)oxy)cyclopropyl)-*N*-(4-(7-methoxy-2,3-dihydrobenzo[1,4-*f*]thiazepin-4(5*H*)-yl)butyl)butan-1-amine (**9**)

To the suspension of NaH (60% in mineral oil, 115 mg, 0.38 mmol) in 2 mL of anhydrous DMF, compound **8** (100 mg, 0.38 mmol) was added and the reaction mixture was stirred at RT for 5 min. Then, bromide **6** (115 mg, 0.38 mmol) was added and the reaction mixture was stirred overnight at RT. After the solvent was removed *in vacuo*, 10 mL of water and 10 mL of DCM were added. The organic solution was separated, and the aqueous solution extracted with DCM (2 × 5 mL). The combined organic solutions were dried over Na_2_SO_4_. The filtrate was evaporated, and the product was isolated by flash column chromatography using a Biotage SNAP Ultra 10 g cartridge (gradient: methanol in DCM 4–40%). Yield −39 mg (21%) of yellowish oil. HPLC: *t*_R_ = 13.7 min (column: Interchim Uptisphere C18-HQ, particle size 10 μm, 250 × 4.6 mm; B/A: 10/90–100/0 in 20 min, flow 1.2 mL/min, 254 nm). ^1^H NMR (400 MHz, CDCl_3_) δ 7.45 (d, *J* = 8.5 Hz, 1H, H_Ar_-5), 7.01 (d, *J* = 2.8 Hz, 1H, H_Ar_-8), 6.73 (dd, *J* = 8.5, 2.8 Hz, 1H, H_Ar_-6), 4.12 (s, 2H, NCH_2_), 3.83 (s, 3H, OCH_3_), 3.36 (d, *J* = 6.8 Hz, 2H, CH_2_), 2.91–2.82 (m, 2H, CH_2_), 2.79–2.75 (m, 2H, CH_2_), 2.67–2.56 (m, 4H, CH_2_), 2.18–2.08 (m, 2H, CH_2_), 1.78–1.71 (m, 2H, CH_2_), 1.68–1.57 (m, 2H, CH_2_), 1.56–1.36 (m, 4H, CH_2_), 0.83 (s, 9H, CH_3_), 0.73–0.64 (m, 2H, CH_2_), 0.45–0.33 (m, 2H, CH_2_), 0.07 (s, 6H, CH_3_). ^13^C NMR (101 MHz, CDCl_3_) δ 159.5, 142.0, 134.0, 127.7, 117.1, 113.3, 58.9, 58.3, 56.4, 52.7, 48.2, 47.7, 38.4, 30.4, 26.1, 25.7, 25.2, 23.5, 17.7, 13.1, −3.4. ESI-MS, positive mode: *m*/*z* (rel. int., %) = 493 (100) [M + H]^+^. HRMS *m*/*z* calcd for C_27_H_49_N_2_O_2_SSi [M + H]^+^, 493.3275; found, 493.3281.

##### *N,N*-bis(4-(1-(*tert*-Butyldimethylsilyl)oxy)cyclopropyl)butyl-*N*-(4-(7-methoxy-2,3-dihydrobenzo[1,4-*f*]thiazepin-4(5*H*)-yl)butan-1-amine (**10**)

To compound **8** (19 mg, 0.07 mmol) in 0.4 mL of 1,2-dichloroethane, aldehyde **5** (36 mg, 0.15 mmol) in 0.4 mL of 1,2-dichloroethane and sodium triacetoxyborohydride (225 mg, 1.06 mmol) were added. The reaction mixture was stirred at room temperature for 1.5 h and concentrated *in vacuo*. The product was isolated by flash chromatography using Biotage HP-Sfär Silica HC Duo 20 μm, 10 g; gradient 5 to 50% methanol in DCM. Yield −13 mg (37%) of yellowish oil. HPLC: *t*_R_ = 17.8 min (column: Interchim Uptisphere C18-HQ, particle size 10 μm, 250 × 4.6 mm; B/A: 10/90–100/0 in 20 min, flow 1.2 mL/min, 254 nm). ^1^H NMR (400 MHz, CDCl_3_) δ 7.44 (d, *J* = 8.4 Hz, 1H, H_Ar_-5), 6.79 (d, *J* = 2.8 Hz, 1H, H_Ar_-8), 6.68 (dd, *J* = 8.4, 2.8 Hz, 1H, H_Ar_-6), 4.09 (s, 2H, NCH_2_), 3.79 (s, 3H, OCH_3_), 3.32–3.26 (m, 2H, CH_2_), 3.00–2.86 (m, 6H, CH_2_), 2.69–2.63 (m, 2H CH_2_), 2.43–2.37 (m, 2H, CH_2_), 1.82–1.70 (m, 6H CH_2_), 1.58–1.45 (m, 10H, CH_2_), 0.84 (s, 18H, CH_3_), 0.71–0.62 (m, 4H, CH_2_), 0.42–0.35 (m, 4H, CH_2_), 0.08 (s, 12H, CH_3_). ^13^C NMR (101 MHz, CDCl_3_) δ 159.2, 145.2, 133.7, 127.9, 117.0, 112.0, 59.8 (CH_2_), 58.5 (CH_2_), 57.0 (CH_2_), 55.5 (OCH_3_), 54.3, 39.2 (CH_2_), 30.4 (CH_2_), 25.9 (CH_3_), 24.1, 17.9, 13.2 (CH_2_), −3.3 (CH_3_). ESI-MS, positive mode: *m*/*z* (rel. int., %) = 719 (100) [M + H]^+^. HRMS *m*/*z* calcd for C_40_H_75_N_2_O_3_SSi_2_ [M + H]^+^, 719.5031; found, 719.5033.

##### 1-(4-((4-(7-Methoxy-2,3-dihydrobenzo[1,4-*f*]thiazepin-4(5*H*)-yl)butyl)amino)butyl)cyclopropan-1-ol (**11a**)

Caution: Cyclopropanols can rearrange into the corresponding ethyl ketones in the presence of acids or strong bases!^30^ Hydrogen fluoride cleavage of TBDMS groups was performed at 0 °C in MeCN. A polyethylene container was charged with **9** (35 mg, 71 μmol) and 1 mL of MeCN and cooled to 0 °C. Then, 15 μL of aq. HF (55%) was added dropwise and the reaction mixture was stirred for 1 h at 0 °C. After the reaction mixture was washed with 5% aq. Na_2_CO_3_, the organic layer was separated and the solvent removed in vacuo. The title compound was isolated by preparative HPLC using reversed-phase cartridge Interchim PF-C18HC 12 g, particle size 30 μm, and gradient elution (B/A, 10/90 → 80/20; B = MeCN + 0.1% TFA; A = H_2_O + 0.1% TFA). Yield −19 mg (71%) of clear oil. ^1^H NMR (400 MHz, DMSO-*d*_6_) δ 7.51 (d, *J* = 8.5 Hz, 1H, H_Ar_-5), 7.30 (d, *J* = 2.8 Hz, 1H, H_Ar_-8), 6.97 (dd, *J* = 8.5, 2.8 Hz, 1H, H_Ar_-6), 4.71 (br. s., 1H, NH), 4.57 (s, 2H, NCH_2_), 3.78 (s, 3H, OCH_3_), 3.71–3.56 (m, 2H, CH_2_), 3.26–2.98 (m, 4H, CH_2_), 2.95–2.85 (m, 4H, CH_2_), 1.84–1.69 (s, 2H, CH_2_), 1.69–1.54 (s, 4H, CH_2_), 1.49–1.38 (s, 4H, CH_2_), 0.52 (t, *J* = 5.5 Hz, 2H), 0.37–0.25 (m, 2H). ^13^C NMR (101 MHz, DMSO-*d*_6_) δ 159.2, 158.1, 133.8, 127.8, 119.1, 114.9, 55.5, 53.5, 46.9, 46.0, 37.5, 25.5, 22.9, 22.6, 20.7, 12.7. ESI-MS, positive mode: *m*/*z* (rel. int., %) = 379 (100) [M + H]^+^. HRMS *m*/*z* calcd for C_21_H_35_N_2_O_2_S [M + H]^+^, 379.2414; found, 379.2415.

##### 1,1′-(((4-(7-Methoxy-2,3-dihydrobenzo[1,4-*f*]thiazepin-4(5*H*)-yl)butyl)azanediyl)bis(butane-4,1-diyl))bis(cyclopropan-1-ol) (**12a**)

The title compound was obtained from **10** (54 mg, 75 μmol) and 90 μL aq. HF (55%), similarly to that described for compound **11a**. After the solvents were removed *in vacuo*, the title compound was isolated by preparative HPLC with gradient elution (B/A: 10/90 → 80/20). Yield −20 mg (54%) of clear oil. ^1^H NMR (400 MHz, CD_3_OD) δ 7.43 (d, *J* = 8.4 Hz, 1H, H_Ar_-5), 6.95 (d, *J* = 2.8 Hz, 1H, H_Ar_-8), 6.78 (d, *J* = 11.3 Hz, 1H, H_Ar_-6), 4.15 (s, 2H, NCH_2_), 3.79 (s, 3H, OCH_3_), 3.36–3.33 (m, 2H, NCH_2_), 3.12–3.01 (m, 2H, CH_2_), 2.80–2.73 (m, 2H, CH_2_), 2.54 (t, *J* = 7.0 Hz, 2H, CH_2_), 1.80–1.67 (m, 6H, CH_2_), 1.67–1.53 (m, 10H, CH_2_), 0.70–0.63 (m, 4H, CH_2_), 0.48–0.39 (m, 4H, CH_2_). ^13^C NMR (101 MHz, CD_3_OD) δ 160.9, 144.4, 134.7, 129.3, 118.5, 113.7, 60.2, 59.6, 55.9, 55.3, 54.1, 53.9, 53.0, 38.6, 31.0, 24.7, 24.7, 24.3, 22.6, 13.6. ESI-MS, positive mode: *m*/*z* (rel. int., %) = 491 (100) [M + H]^+^. HRMS *m*/*z* calcd for C_28_H_47_N_2_O_3_S [M + H]^+^, 491.3302; found, 491.3289.

4-(1-((*tert*-Butyldimethylsilyl)oxy)cyclopropyl)-*N*-(3-(4-methoxyphenoxy)propyl)butan-1-amine (**13**) was obtained from 3-(4-methoxyphenoxy)propan-1-amine (BLD Pharm, 180 mg, 1.00 mmol), 307 mg (1.0 mmol) (1-(4-bromobutyl)cyclopropoxy)(*tert*-butyl)dimethylsilane **6**, and 120 mg of NaH (60% in mineral oil) using the same procedure, as described for compound **9**. The title compound was isolated by flash column chromatography using Biotage HP-Sfär Silica HC Duo cartridge 20 μm, 25 g (gradient: methanol in DCM 2% – 20%). Yield −167 mg (41%) of yellowish solid. ^1^H NMR (400 MHz, CDCl_3_) δ 6.85–6.77 (m, 4H, H_Ar_), 3.98 (t, *J* = 6.2 Hz, 2H, NCH_2_), 3.75 (s, 3H, OCH_3_), 2.80 (t, *J* = 7.0 Hz, 2H, NCH_2_), 2.67–2.58 (m, 2H), 2.01–1.91 (m, 2H), 1.57–1.44 (m, 6H, CH_2_), 0.84 (s, 9H, CH_3_), 0.70–0.62 (m, 2H, CH_2_), 0.43–0.34 (m, 2H, CH_2_), 0.07 (s, 6H, CH_3_). ^13^C NMR (101 MHz, cdcl_3_) δ 153.7, 153.1, 115.4, 114.6, 67.0, 56.8, 55.7, 55.7, 50.0, 46.9, 39.0, 30.0, 29.7, 25.7, 23.8, 17.8, 13.0, −3.4. ESI-MS, positive mode: *m*/*z* (rel. int., %) = 408 (100) [M + H]^+^. HRMS *m*/*z* calcd for C_23_H_42_NO_3_Si [M + H]^+^, 408.2928; found, 408.2931.

*N,N*-bis(4-(1-(*tert*-Butyldimethylsilyl)oxy)cyclopropyl)butyl-*N*-(3-(4-methoxyphenyl)oxy)propan-1-amine (**14**) was prepared from 3-(4-methoxyphenoxy)propan-1-amine (BLD Pharm, 30 mg, 0.17 mmol) and aldehyde **5** (100 mg, 0.41 mmol) in DCE according to the procedure described for compound **10**. The title compound was isolated by flash column chromatography using Biotage HP-Sfär Silica HC Duo 20 μm, 10 g (gradient: methanol in DCM 2% – 40%). Yield −60 mg (55%) of yellowish oil. HPLC: *t*_R_ = 5.1 min (B/A: 30/70–100/0 in 15 min, flow 1.2 mL/min, 254 nm). ^1^H NMR (400 MHz, CDCl_3_) δ 6.85–6.77 (m, 4H, H_Ar_), 3.99–3.90 (m, 2H, NCH_2_), 3.80–3.73 (m, 3H, OCH_3_), 3.68–3.61 (m, 2H, NCH_2_), 2.69–2.60 (m, 2H, CH_2_), 2.50–2.42 (m, 2H, CH_2_), 1.63–1.41 (m, 14H, CH_2_), 0.88–0.79 (m, 18H, CH_3_), 0.70–0.62 (m, 4H, CH_2_), 0.45–0.35 (m, 4H, CH_2_), 0.13–0.02 (m, 12H, CH_3_). ^13^C NMR (101 MHz, CDCl_3_) δ 153.7, 153.1, 115.4, 114.6, 63.0, 56.8, 55.7, 54.2, 50.6, 39.0, 38.9, 32.8, 25.7, 23.9, 22.2, 17.8, 13.0, −3.4. ESI-MS, positive mode: *m*/*z* (rel. int., %) = 634 (100) [M + H]^+^. HRMS *m*/*z* calcd for C_36_H_68_NO_4_Si_2_ [M + H]^+^, 634.4681; found, 634.4665.

##### 1-(4-((3-(4-Methoxyphenoxy)propyl)amino)butyl)cyclopropan-1-ol (**11b**)

*tert*-Butyldimethylsilyl deprotection of **13** (30 mg, 0.07 mmol) was performed in 140 μL of 1 M TBAF in THF. The reaction mixture was stirred overnight at RT. The title compound was isolated by preparative HPLC with gradient elution (B/A: 20/80 → 100/0). Yield 5 mg (24%) of clear oil. ^1^H NMR (400 MHz, CDCl_3_) δ 6.82 (s, 4H, H_ar_), 3.97 (t, *J* = 6.1 Hz, 2H, OCH_2_), 3.76 (s, 3H,OCH_3_), 2.78 (t, *J* = 7.0 Hz, 2H, CH_2_), 2.70–2.62 (m, 2H, CH_2_), 1.98–1.88 (m, 2H, CH_2_), 1.69–1.52 (m, 7H, CH_2_, OH), 0.99 (t, *J* = 7.3 Hz, 1H, NH), 0.74–0.67 (m, 2H, CH_2_), 0.43–0.36 (m, 2H, CH_2_). ^13^C NMR (101 MHz, CDCl_3_) δ: 153.9 (C), 153.2 (C), 115.5 (CH), 114.8 (CH), 67.2 (CH), 55.9 (CH), 55.1 (C), 49.8 (CH), 47.1 (CH), 38.2 (CH), 29.8 (CH), 29.6 (CH), 23.6 (CH), 13.8 (CH). ESI-MS, positive mode: *m*/*z* (rel. int., %) = 294 (100) [M + H]^+^. HRMS *m*/*z* calcd for C_17_H_28_N_3_O_3_ [M + H]^+^, 294.2064; found, 294.2068.

1-(4-((3-(4-Methoxyphenoxy)propyl)amino)butyl)cyclopropan-1-ol (**12b**) was obtained by *t*-butyldimethylsilyl deprotection of **14** (34 mg, 0.05 mmol) in DMF using 48 mg (0.82 mmol) of KF together with 112 mg (0.82 mmol) Et_3_N × HCl. The reaction mixture was stirred overnight at 70 °C. After the precipitate was filtered off, DMF was evaporated *in vacuo*. The title compound was isolated by flash column chromatography using Biotage HP-Sfär Silica HC Duo 20 μm, 10 g (gradient: methanol in DCM 2–20%). Yield −13 mg (64%). ^1^H NMR (400 MHz, CDCl_3_) δ 6.85–6.75 (m, 4H, H_ar_), 4.01 (q, *J* = 5.2 Hz, 2H, CH_2_), 3.75 (s, 3H, CH_3_), 3.29–3.20 (m, 2H, CH_2_), 3.15–3.02 (m, 4H, CH_2_), 2.35–2.26 (m, 2H, CH_2_), 1.99–1.86 (m, 4H, CH_2_), 1.70–1.53 (m, 8H, CH_2_), 0.77–0.70 (m, 4H, CH_2_), 0.42–0.34 (m, 4H, CH_2_). ^13^C NMR (126 MHz, CDCl_3_) δ: 154.3, 152.4, 115.5, 114.9, 65.5, 55.8, 54.9, 53.2, 50.8, 37.3, 23.9, 23.6, 23.2, 13.7. ESI-MS, positive mode: *m*/*z* (rel. int., %) = 406 (100) [M + H]^+^. HRMS *m*/*z* calcd for C_24_H_40_NO_4_ [M + H]^+^, 406.2952; found, 406.2957.

##### 3-((4-Methoxyphenyl)thio)propan-1-amine (**15**)

4-Methoxybenzenethiol (BLD Pharm, 280 mg, 2 mmol) was added to the solution of 3-bromopropan-1-amine hydrobromide (BLD Pharm, 432 mg, 2 mmol) in 6 mL of ethanol containing potassium carbonate (560 mg, 4 mmol). Then, the reaction mixture was heated to reflux. After 12 h, it was poured into cold water (10 mL) and extracted with CH_2_Cl_2_ (4 × 5 mL). The extracts were combined, washed with water (3 × 5 mL), and dried over anhydrous Na_2_SO_4_. The solvent was evaporated *in vacuo*, affording the title compound as a yellowish oil. Yield −265 mg (67%). ^1^H NMR (400 MHz, CD_3_OD) δ 7.37–7.30 (m, 2H, H_Ar_), 6.90–6.83 (m, 2H, H_Ar_), 3.77 (s, 3H, OCH_3_), 2.86 (t, *J* = 7.2 Hz, 2H, CH_2_), 2.73 (t, *J* = 7.1 Hz, 2H, CH_2_), 1.71 (p, *J* = 7.1 Hz, 2H, CH_2_). ^13^C NMR (101 MHz, CD_3_OD) δ 160.5, 134.3, 127.7, 115.6, 55.8, 41.3, 33.9, 33.1. ESI-MS, positive mode: *m*/*z* (rel. int., %) = 198 (100) [M + H]^+^.

4-(1-((*tert*-Butyldimethylsilyl)oxy)cyclopropyl)-*N*-(3-((4-methoxyphenyl)thio)propyl)butan-1-amine (**16**) and *N,N*-bis(4-(1-((*tert*-butyldimethylsilyl)oxy)cyclopropyl)butyl-*N*-(3-(4-methoxyphenyl)thio)propan-1-amine (**17**) were obtained from amine **15** (168 mg, 0.85 mmol) and bromide **6** (644 mg, 2.1 mmol) in anhydrous DMF according to the procedure as described for compound **9**. The title compounds were isolated by flash column chromatography using Biotage HP-Sfär Silica HC Duo 20 μm, 25 g (gradient: methanol in DCM 2–40%). Yield −90 mg (21%) of *N*-mono- (**16**) and 100 mg (18%) of *N*,*N*-disubstituted compound **17**. Compound **16**: ^1^H NMR (400 MHz, CDCl_3_) δ 7.34 (d, *J* = 8.9 Hz, 2H, H_Ar_), 6.83 (d, *J* = 8.9 Hz, 2H, H_Ar_), 3.78 (s, 3H, OCH_3_), 2.87 (t, *J* = 7.2 Hz, 2H, CH_2_), 2.76 (t, *J* = 7.1 Hz, 2H, CH_2_), 2.63 (t, *J* = 7.0 Hz, 2H, CH_2_), 1.82 (p, *J* = 7.1 Hz, 2H, CH_2_), 1.62–1.41 (m, 6H, CH_2_), 0.84 (s, 9H), 0.70–0.62 (m, 2H, CH_2_), 0.44–0.29 (m, 2H, CH_2_), 0.07 (s, 6H, CH_3_). ^13^C NMR (101 MHz, CDCl_3_) δ: 159.1, 133.4, 126.4, 114.7, 114.6, 56.9, 55.5, 49.8, 48.4, 39.2, 39.1, 33.8, 29.5, 29.1, 25.9, 23.8, 17.9, 13.2, 13.2, −3.3, −3.3. ESI-MS, positive mode: *m*/*z* (rel. int., %) = 424 (100) [M + H]^+^. HRMS *m*/*z* calcd for C_23_H_42_NO_2_SSi [M + H]^+^, 424.2700; found, 424.2698. Compound **17**: ^1^H NMR (400 MHz, CDCl_3_) δ 7.32 (d, *J* = 8.9 Hz, 1H, H_ar_), 6.82 (d, *J* = 8.9 Hz, 1H, H_ar_), 3.78 (s, 3H, OCH_3_), 2.84 (t, *J* = 7.1 Hz, 2H), 2.60–2.32 (m, 4H, CH_2_), 1.86–1.61 (m, 4H, CH_2_), 1.60–1.37 (m, 12H, CH_2_), 0.84 (d, *J* = 1.2 Hz, 18H, CH_3_), 0.70–0.63 (m, 4H, CH_2_), 0.43–0.33 (m, 2H, CH_2_), 0.07 (s, 12H, CH_3_). ^13^C NMR (101 MHz, CDCl_3_) δ: 133.2, 114.7, 63.1, 56.9, 56.9, 55.4, 39.1, 39.0, 33.9, 32.9, 31.6, 26.2, 24.0, 22.3, 17.9, 13.2, 13.2, 8.4, −1.7, −3.3, −3.3. ESI-MS, positive mode: *m*/*z* (rel. int., %) = 650 (100) [M + H]^+^. HRMS *m*/*z* calcd for C_36_H_68_NO_3_SSi_2_ [M + H]^+^, 650.4453; found, 650.4434.

##### 1-(4-((3-((4-Methoxyphenyl)thio)propyl)amino)butyl)cyclopropan-1-ol (**11c**)

*tert*-Butyldimethylsilyl deprotection of **16** (90 mg, 0.21 mmol) was performed in 350 μL of 1 M TBAF in THF. The reaction mixture was stirred overnight at RT. The title compound was isolated by preparative HPLC with gradient elution (B/A: 10/90 → 100/0). Yield −43 mg (67%) of yellowish oil. ^1^H NMR (400 MHz, CD_3_OD) δ 7.46–7.32 (m, 2H, H_ar_), 6.95–6.85 (m, 2H, H_ar_), 3.78 (s, 3H, OCH_3_), 3.15–3.04 (m, 2H, CH_2_), 3.05–2.93 (m, 2H, CH_2_), 2.91 (t, *J* = 7.0 Hz, 2H), 1.94 (p, *J* = 7.1 Hz, 2H, CH_2_), 1.80–1.65 (m, 2H, CH_2_), 1.64–1.52 (m, 4H, CH_2_), 0.72–0.57 (m, 2H, CH_2_), 0.48–0.32 (m, 2H, CH_2_). ^13^C NMR (101 MHz, CD_3_OD) δ 160.8 (C), 134.7 (CH), 126.5 (C), 115.8 (CH), 55.8 (CH_3_), 55.2 (C), 49.3 (CH), 49.1 (CH), 47.6 (CH), 38.6 (CH), 33.3 (CH), 27.0 (CH), 26.8 (CH), 24.1 (CH), 13.6 (CH). ESI-MS, positive mode: *m*/*z* (rel. int., %) = 310 (100) [M + H]^+^. HRMS *m*/*z* calcd for C_17_H_28_NO_2_S [M + H]^+^, 310.1835; found, 310.1839.

##### 1,1′-(((3-((4-Methoxyphenyl)thio)propyl)azanediyl)bis(butane-4,1-diyl))bis(cyclopropan-1-ol) (**12c**)

Desilylation of **17** (107 mg, 0.15 mmol) was performed in 450 μL of 1 M TBAF in THF. The reaction mixture was stirred overnight at RT. The title compound was isolated by flash column chromatography using Biotage HP-Sfär Silica HC Duo 20 μm, 10 g (gradient: methanol in DCM 8–20%). Yield 33 mg (52%) of yellowish oil. ^1^H NMR (400 MHz, CDCl_3_) δ 7.36–7.30 (m, 2H, H_Ar_), 6.86–6.79 (m, 2H, H_Ar_), 3.78 (s, 3H, OCH_3_), 2.83 (t, *J* = 6.9 Hz, 2H, CH_2_), 2.70–2.61 (m, 2H, CH_2_), 2.53 (t, *J* = 6.7 Hz, 2H, CH_2_), 1.76 (dd, *J* = 8.7, 6.3 Hz, 2H, CH_2_), 1.59–1.40 (m, 12H, CH_2_), 0.74–0.65 (m, 2H, CH_2_), 0.41–0.33 (m, 2H, CH_2_). ^13^C NMR (101 MHz, CDCl_3_) δ: 169.1, 159.0, 133.3, 126.2, 114.8, 55.5, 55.1, 53.7, 52.1, 37.9, 33.8, 25.8, 23.8, 13.7. ESI-MS, positive mode: *m*/*z* (rel. int., %) = 422 (100) [M + H]^+^. HRMS *m*/*z* calcd for C_17_H_28_NO_2_S [M + H]^+^, 422.2704; found, 422.2706.

4-(4-(1-((*tert*-Butyldimethylsilyl)oxy)cyclopropyl)butyl)-7-methoxy-2,3,4,5-tetrahydrobenzo[1,4-*f*]thiazepine (**18**) was prepared from commercial 7-methoxy-2,3,4,5-tetrahydrobenzo[1,4-*f*]thiazepine (90 mg, 0.62 mmol) and aldehyde **5** (174 mg, 0.7 mmol) in DCE according to the procedure described for compound **10**. The title compound was isolated by flash column chromatography using Biotage HP-Sfär Silica HC Duo 20 μm, 10 g (gradient: methanol in DCM 2% – 40%). Yield −111 mg (43%) of yellowish oil. ^1^H NMR (400 MHz, CDCl_3_) δ 7.47 (d, *J* = 8.5 Hz, 1H, H_Ar_), 6.87 (d, *J* = 2.8 Hz, 1H, H_Ar_), 6.76 (dd, *J* = 8.5, 2.8 Hz, 1H, H_Ar_), 4.30 (s, 2H, CH_2_), 3.79 (s, 3H, OCH_3_), 3.49–3.41 (m, 2H, CH_2_), 2.85–2.79 (m, 2H, CH_2_), 2.58–2.49 (m, 2H, CH_2_), 1.67 (d, *J* = 9.6 Hz, 2H, CH_2_), 1.47 (d, *J* = 3.7 Hz, 4H, CH_2_), 0.82 (s, 9H, CH_3_), 0.65 (d, *J* = 11.8 Hz, 2H, CH_2_), 0.37 (d, *J* = 11.9 Hz, 2H, CH_2_), 0.05 (s, 6H, CH_3_). ^13^C NMR (101 MHz, CDCl_3_) δ: 159.6, 140.2, 134.0, 127.8, 117.9, 113.4, 58.7, 56.8, 55.5, 53.6, 51.8, 39.0, 28.8, 26.0, 25.8, 23.8, 17.9, 13.2, −3.3. ESI-MS, positive mode: *m*/*z* (rel. int., %) = 422 (100) [M + H]^+^. HRMS *m*/*z* calcd for C_23_H_40_NO_2_SSi [M + H]^+^, 422.2544; found, 422.2545.

##### 1-(4-(7-Methoxy-2,3-dihydrobenzo[1,4-*f*]thiazepin-4(5H)-yl)butyl)cyclopropan-1-ol (**11d**)

Cleavage of *tert*-butyldimethylsilyl ether **18** (148 mg, 0.36 mmol) was performed in 710 μL of 1 M TBAF in THF, as described for compound **12c**. After the solvent was evaporated *in vacuo*, the title compound was isolated using flash column chromatography (Biotage HP-Sfär Silica HC Duo 20 μm, 10 g, gradient: methanol in DCM 8% – 10%) followed by preparative HPLC with gradient elution (MeCN/H_2_O: 10/90 → 100/0). Yield 22 mg (20%) of clear oil. ^1^H NMR (400 MHz, CDCl_3_) δ 7.45 (d, *J* = 8.4 Hz, 1H, H_Ar_), 6.82 (d, *J* = 2.7 Hz, 1H, H_Ar_), 6.71 (dd, *J* = 8.5, 2.8 Hz, 1H, H_Ar_), 4.16 (s, 2H, CH_2_), 3.80 (s, 3H, CH_3_), 3.41–3.29 (m, 2H, CH_2_), 2.79–2.66 (m, 2H, CH_2_), 2.46 (t, *J* = 7.0 Hz, 2H, CH_2_), 1.68–1.46 (m, 6H, CH_2_), 0.78–0.65 (m, 2H, CH_2_), 0.48–0.30 (m, 2H, CH_2_). ^13^C NMR (101 MHz, CDCl_3_) δ: 159.2, 133.6, 127.7, 117.3, 112.3, 59.2, 57.8, 55.4, 55.3, 51.5, 37.9, 29.6, 26.6, 23.4, 13.7. ESI-MS, positive mode: *m*/*z* (rel. int., %) = 308 (100) [*M*+H]^+^. HRMS *m*/*z* calcd for C_17_H_26_NO_2_S, 308.1679 [M + H]^+^; found, 308.1679.

### Cell Culture

HEK-293 cells stably expressing R-CEPIA1*er* and WT RyR2 (generous gift from Takashi Murayama, Juntendo University School of Medicine, Tokyo) were cultured in Dulbecco’s modified Eagle's medium (DMEM, Thermo Fisher Scientific, Darmstadt, Germany) supplemented with 10% fetal bovine serum (FBS, Thermo Fisher Scientific, Darmstadt, Germany), 0.9% penicillin/streptomycin (Merck KGaA, Darmstadt, Germany), 15 μg/mL blasticidin (Invivogen, Toulouse, France), 100 μg/mL hygromycin (Merck KGaA, Darmstadt, Germany), and 400 μg/mL G418 (Thermo Fisher Scientific, Darmstadt, Germany) in a humidified 5% CO_2_ incubator at 37 °C. To induce the expression of WT RyR2, 2 μg/mL doxycycline (Merck KGaA, Darmstadt, Germany) was added 24 h before experiments.

HL-1 cells (SCC065, Merck KGaA, Darmstadt, Germany) were cultivated in Claycomb Medium (51800C, Merck KGaA, Darmstadt, Germany), supplemented with Glutamax, 10% fetal bovine serum, 0.9% penicillin/streptomycin, and 0.1 mM noradrenaline (Merck KGaA, Darmstadt, Germany) on fibronectin/gelatin (Merck KGaA, Darmstadt, Germany) coated culture bottles or plates 2 days before plating.

### Fluorescence Microscopy

Confocal and STED images were acquired in an Abberior STED 775 QUAD scanning microscope (Abberior Instruments GmbH) equipped with 488, 561, and 640 nm 40 MHz pulsed excitation lasers, a pulsed 775 nm STED 40 MHz laser, and a UPlanSApo 100*×*/1.40 Oil objective. Pixel size was 30–40 nm for all STED images acquired on this setup.

### Time-Lapse [Ca^2+^]_ER_ Assay

The fluorescence plate reader [Ca^2+^]_ER_ assay was performed according to the slightly modified protocol of Murayama and Kurebayashi.^36^ HEK-293 WT RyR2 R-CEPIA1*er* cells (50,000 cells per well) were cultivated for 24 h in black-walled, clear-bottom 96-well microplates (Corning, Amsterdam, The Netherlands) covered with fibronectin (10 μg/mL, Roche, Mannheim, Germany) in a humidified incubator at 37 °C and 5% CO_2_. After the cells were inducted with doxycycline for 24 h, changes in ER calcium concentrations [Ca^2+^]_ER_ were measured on a multiwell plate reader TECAN Spark 20 M at 37 °C. The 1000 × stock solutions of the test compounds were prepared in DMSO. Briefly, the culture medium was removed and 100 μL of Tyrode’s solution containing 2 mM CaCl_2_ was added to the cells. The time courses were recorded in each well. Fluorescence was evoked by a 560 nm excitation wavelength and collected in a bottom-read mode at 610 nm. Data was recorded every 10 s, exposure −20 flashes, excitation bandwidth −20 nm, emission bandwidth −20 nm. 100 μL of 0–100 μM solution of test compound in Tyrode’s solution containing 2 mM CaCl_2_ was injected in each well (speed 100 μL/s) at the 90 s time point. *F*/*F*_0_, the ratio of the averaged fluorescence intensities between the initial (before injection compound *F*_0_) and the last (*F*) 100 s of the readout was taken for the analysis. The 0.1 v/v% DMSO and 2 mM CaCl_2_ Tyrode’s solution was used as a control.

The dose–response curves were calculated using the software package GraphPad Prism version 8.3.1. (GraphPad Software, Inc.). All data are presented as mean ± SD in three independent experiments. *Y* = bottom + (top – bottom)/(1 + 10^((LogEC_50_ – *X*) × HillSlope))^ was used, where HillSlope describes the steepness of the curve, and top and bottom are plateaus in the units of the *Y-*axis.

### Caffeine Assay on HL-1 Cells

HL-1 cells (50,000 per well) were cultivated for 24 h in black-walled, clear-bottom 96-well microplates (Corning, Amsterdam, The Netherlands) covered with fibronectin/gelatin (Merck KGaA, Darmstadt, Germany) in a humidified incubator at 37 °C and 5% CO_2_.

Changes in Ca^2+^ concentration were measured on a multiwell plate reader TECAN Spark 20 M using a FLIPR 6 Calcium Assay Kit from Molecular Devices (Molecular Devices LLC, München, Germany) at 37 °C. The 1000 × stock solutions of the test compounds were prepared in DMSO. A 100 μL solution of test compound in Tyrode’s solution containing FLIPR Calcium 6 dye was incubated for 2 h at 37 °C and 5% CO_2_ in a humidified incubator. The time courses were recorded in each well. Fluorescence was evoked at 485 nm excitation wavelength and read out in a bottom-read mode at 525 nm. Data were recorded every 2 s, exposure −20 flashes, excitation bandwidth −10 nm, emission bandwidth −15 nm. To initiate Ca^2+^ influx, 25 μL of 50 mM caffeine solution in Tyrode’s solution containing 2 mM CaCl_2_ was injected in each well (speed 100 μL/s) at the 10 s time point, resulting in 10 mM final concentration in the well. The differences between the caffeine-induced peak minus basal fluorescence, Δ*F*_Caff_, were taken into account for the analysis. The Tyrode’s solution +0.1 v/v% DMSO was used as a control. The dose–response curves were calculated using the software package GraphPad Prism version 8.3.1. All data are presented as mean ± SD from eight independent experiments. *Y* = bottom + (top – bottom)/(1 + 10^((LogEC_50_ – *X*) × HillSlope))^ was used, where HillSlope describes the steepness of the curve and top and bottom are plateaus in the units of the *Y* axis.

### Isolation of Microsomal Membrane Vesicles from HEK-293 Cells

Isolation of microsomal membrane vesicles from HEK-293T cells (HEK-293 ER) was performed according to Stewart et al.^61^ HEK-293T cells were harvested with Trypsin/EDTA, washed twice with ice-cold PBS containing protease inhibitor cocktail (cOmplete, Roche, Mannheim, Germany), and centrifuged 800 × *g* for 5 min. The pellet was resuspended in ice-cold lysis buffer (1 mM EDTA, 10 mM HEPES, cOmplete, pH 7.4) and incubated on ice for 20 min. After the cells were homogenized with a tight-fitting Dounce homogenizer (10–20 strokes), an equal volume of restoration buffer (500 mM sucrose, 10 mM HEPES, cOmplete, pH 7.2) was added and the cells were homogenized again (10–20 strokes). The homogenate was centrifuged at 10,000 × *g* for 20 min at 4 °C. The supernatant was collected and centrifuged again for another 2 h (10^5^ × g at 4 °C). Then, the pellet was carefully resuspended in resuspension buffer (250 mM sucrose, 10 mM HEPES, cOmplete, pH 7.2) on ice by pipetting the solution up and down until it was fully dissolved. The obtained solution was diluted to a protein concentration of 15–20 μg/μL with the resuspension buffer; the aliquots were snap frozen in liquid nitrogen and stored at −80 °C until usage.

### Isolation of Microsomal Membrane Vesicles from Mouse Hearts

All animal procedures were performed in accordance with Directive 2010/63/EU of the European Parliament and the Council on the protection of animals used in research as well as Animal Welfare Law of the Federal Republic of Germany (Tierschutzgesetz der Bundesrepublik Deutschland, TierSchG). Sacrificing rodents for subsequent preparation of tissue did not require specific authorization or notification (§7 Abs. Two Satz 3 TierSchG). All mice were housed with a 12 h light/dark cycle with free access to food and water. Adult C57BL/6N mice of both genders were sedated with isoflurane in a sealed container and quickly euthanized by cervical dislocation. Heart ventricles were taken out instantly, washed with ice-cold PBS plus complete, cut into smaller pieces, and snap frozen in liquid nitrogen straight away.

Lysis buffer thawed on ice (10 mM EDTA, 10 mM HEPES, cOmplete, pH 7.4) was added, and the ventricles were homogenized with a Qiagen Cellruptor for 20 s. On ice, an equal volume of restoration buffer (500 mM sucrose, 10 mM HEPES, cOmplete, pH7.2) was added and briefly mixed. The homogenate was centrifuged at 10^4^ × g for 20 min at 4 °C. The supernatant was collected and centrifuged again for another hour (10^5^ × g at 4 °C). Then, the pellet was carefully resuspended in resuspension buffer (250 mM sucrose, 10 mM HEPES, cOmplete, pH 7.2) on ice by pipetting the solution up and down until fully dissolved. The obtained solution was diluted to a protein concentration of 5 μg/μL with the resuspension buffer, and the aliquots were snap frozen in liquid nitrogen and then stored at −80 °C until usage.

### SERCA2 Activity Measurements

To determine whether new compounds could increase the SERCA2 activity, HEK-293 microsomes (40–150 μg of total protein) or mouse ventricular microsomes (1–20 μg of total protein) were diluted in 100 μL of the assay buffer used for measurement of SERCA activity (described below) and incubated at room temperature for 5 min prior to the experiments. The measurements of SERCA2 activity were made at 28 °C by using a NADH-coupled ATPase assay as described in Radnai et al. with slight modifications.^51^ Briefly, the assay buffer contained 60 mM MOPS, 120 mM KCl, 6 mM MgCl_2_, 1 mM EGTA, 5 mM NaN_3_, 0.5 mM phosphoenolpyruvate, and 1.5 mM CaCl_2_, pH 7.0. Before the reaction was started by the injection of 5 mL of ATP (resulting in 2 mM final concentration in the well), five units/mL of both lactate dehydrogenase and pyruvate kinase, 0.5 mM NADH, and 1 μM Ca^2+^ ionophore A-23187 (Sigma, C-7522) were added to the diluted sample with or without the tested compound. The 1.5 mM Ca/EGTA buffer has a free Ca^2+^ concentration of about 200 μM. The NADH fluorescence decrease was recorded each 2 s for a 10 min course on a multiwell plate reader TECAN Spark 20 M in 96-well glass bottom plates (MatTek Corporation; Cat. No. PBK96G-1.5-5-F). Fluorescence was evoked by a 380 nm excitation wavelength and collected in a bottom-read mode at 425 nm. Exposure −30 flashes, excitation bandwidth: 10 nm, emission bandwidth: 10 nm. ATP was injected into the well at 30 s. The amount of added protein, 7–13 μg/mL for mouse SR and 133–270 μg/mL for HEK ER, did not affect the slope of the fluorescence decrease in the control samples (0.1% DMSO). The data were analyzed with the computer software GraphPad Prism version 8.3.1 (GraphPad Software, Inc.). The difference in signal decrease with and without microsomes was taken as an indicator of the additional ATPase activity performed by SERCA2. The obtained values for maximal SERCA2 activity were normalized for the total protein concentration. Maximal SERCA2 activity values are reported as percentages of the control (0.1% DMSO) values. Microsomal samples were kept on ice until usage and set to 100%.

### Statistical Analysis

Normality and log normality tests were performed using the Shapiro–Wilk test. The significance between means was tested using unpaired Student’s *t*-test and one-way ANOVA. The Dunnett test was performed to compare every mean to a control (0.1% DMSO) mean. Results are represented as mean ± SD. A value of *P* < 0.05 was considered significant.

### Western Blot Analysis

SERCA2a protein levels in microsomal membrane vesicles (HEK-293T cells and mouse hearts, 8–11-week-old mice, *n* = 10 males and 2 females, C57Bl/6N genotype) were determined by Western blot analysis. 20 μL of microsomes were mixed with 2 μL of NuPAGE LDS Sample buffer (Thermo Fisher) and heated at 70 °C for 10 min. The samples (100–150 μg protein) were loaded onto a 3–8% gradient Tris–acetate gel or 4–20% SDS–Tris–glycine gel, and the gel was run for 1 h at 150 V in running buffer. After that, the gel was equilibrated for 10 min in 20% EtOH, transferred onto a nitrocellulose membrane, and blotted with the iBlot 2 system (dry blot) for 10 min at 25 V. Membranes were blocked with 5% skim milk for 30 min at room temperature and incubated with primary antibodies against SERCA2a (Badrilla A010-20 rabbit polyclonal, 1:1000) overnight at 4 °C with moderate shaking. Binding of the primary antibody was detected by a horseradish peroxidase (HRP)-conjugated secondary antibody (1 h, RT). The detection was done with the Western Lightning Plus ECL Kit (PerkinElmer LAS GmbH, Rodgau) and an Amersham Imager 600.

### Cytotoxicity

Cell viability was assessed using the CytoTox-Glo Cytotoxicity Assay according to the manufacturing procedure (Promega GmbH). Briefly, HL-1 cells were cultivated for 24 h in black-walled, clear-bottom 96-well microplates (Corning) covered with fibronectin in DMEM, containing different concentrations of the tested compound (10,000 cells/well). Luminescence of a luminogenic peptide substrate was measured before and after the addition of the lysis reagent (digitonin). The viable cell contribution was determined by a subtractive method. 0.1% DMSO-treated cells were taken as a control.

### Membrane Permeability

Membrane permeability was assessed using PermeaPad Plate according to the manufacturing procedure (innoME GmbH, Espelkamp, Germany). Briefly, 200 μL of water was added into the acceptor plate. 200 μL of 100–500 μM test compound in water was added directly to the well membranes of the donor plate. The donor plate contains a biomimetic membrane for simulating passive mass transfer through different barriers in the body. Then, the donor plate was placed into the acceptor plate wells and incubated at room temperature for 24 h in the dark. To determine the peak absorbance of test compounds, absorbance spectra of acceptor solutions and initial solutions for each test compound were read out. The permeability rate (*P*_e_) was calculated using the formula:

, where OD_A_ is the absorbance of acceptor solution and OD_E_ is the absorbance of the standard solution; if the compound is able to permeabilize the membrane and fully reach equilibrium, 250 μM will be the final concentration of solution in the donor and acceptor wells. The coefficient *C* was calculated using the formula:

, where *V*_A_ is the acceptor volume (cm^3^), *V*_D_ is the donor volume (cm^3^), the membrane area is 0.24 cm^2^, and the time is 86,400 s.

## Supporting Information Available

The Supporting Information is available free of charge via the Internet at http://pubs.acs.org.”^1^H and ^13^C NMR spectra, LC-MS and HPLC spectra, and supplementary methods (resolution measurements, time-lapse fluorescence measurements, functional measurements in isolated ER vesicles, Western blot analysis, and cell viability measurements) (PDF)
Molecular formula strings (CSV)

## Abbreviations

CAMKII: calcium/calmodulin-dependent kinase
DMEM: Dulbecco’s modified Eagle's medium
ER: endoplasmic reticulum
EtMgBr: ethylmagnesium bromide
FKBP12.6: peptidyl-propyl-*cis*-*trans* isomerase calstabin2
HBSS: Hanks’ balanced salt solution
HEK-293: human embryonic kidney 293 cells
HEPES: 4-(2-hydroxyethyl)-1-piperazineethanesulfonic acid
HF: heart failure
HL-1: atrial muscle cells
NCX: sodium–calcium exchanger
PLB: phospholamban
RyR2: ryanodine receptor 2
SERCA2a: sarco/endoplasmic reticulum Ca^2+^-ATPase 2a
SR: sarcoplasmic reticulum
STED: stimulated emission depletion
TBAF: tetrabutylammonium fluoride
TBDMS: *tert*-butyldimethylsilyl
TSTU: *N,N,N′,N′*-tetramethyl-*O*-(*N*-succinimidyl)uranium tetrafluorborat
