# Supplementary material for: 1,4-Benzothiazepines with Cyclopropanol Groups and Their Structural Analogues Exhibit Both RyR2-Stabilizing and SERCA2a-Stimulating Activities
Source: J Med Chem. 2023 Nov 22;66(23):15761–75. doi: 10.1021/acs.jmedchem.3c01235 (PMC10726367; doi:10.1021/acs.jmedchem.3c01235)
Supplement: Supplementary file 1 — jm3c01235_si_001.pdf [file jm3c01235_si_001.pdf]

SUPPORTING INFORMATION

1,4-BENZOTHIAZEPINES WITH CYCLOPROPANOL GROUPS AND THEIR STRUCTURAL ANALOGUES EXHIBIT BOTH RYR2 STABILIZING AND SERCA2A STIMULATING ACTIVITIES

Gyuzel Y. Mitronova[1,3*], Christine Quentin[1], Vladimir N. Belov[1], Jörg Wegener[2,3*], Kamila A. Kiszka[1], Stephan E. Lehnart[2,3]

[1] Department of NanoBiophotonics, Max Planck Institute for Multidisciplinary Sciences, Am Fassberg 11, 37077 Göttingen, Germany

[2] Department of Cardiology & Pulmonology, Heart Research Center Göttingen, University Medical Center Göttingen, Robert-Koch-Str 42a, 37075 Göttingen, Germany

[3] German Centre for Cardiovascular Research (DZHK), Partner Site Göttingen, 37075 Göttingen, Germany

*Corresponding Authors:

Gyuzel Y. Mitronova (G.Y.M); E-Mail: gyuzel.mitronova@mpinat.mpg.de;

Jörg Wegener (J.W); E-Mail: joerg.wegener@med.uni-goettingen.de

CONTENTS

SUPPLEMENTARY FIGURES 4
Figure S1. Resolution measurements for R-CEPIA1er in living HEK-293 RyR2 R-CEPIA1er cells........ 4
Figure S2. Time-lapse fluorescence measurements using TECAN Spark 20M plate reader with HEK293 cells expressing WT RyR2 and R-CEPIA1er indicator. 5
Figure S3. Caffeine-induced Ca^{2+} release assay. 6
Figure S4. S36 and S107 have shown no concentration dependent effect in caffeine-induced Ca^{2+} release assay 6
Figure S5. Western blot analysis of HEK293 ER-vesicles. 7
Figure S6. Western blot analysis of mouse ventricular microsomes. 7
Figure S8. Cell viability after 24-hour incubation with test compounds. 9
NMR SPECTRA 10
Figure S9. ^{1}H spectrum of ARM210........ 10
Figure S10. ^{1}H and ^{13}C spectra of compound 7. 11
Figure S11. ^{1}H and ^{13}C spectra of compound 8. 12
Figure S12. ^{1}H and ^{13}C spectra of compound 9. 13
Figure S13. ^{1}H and ^{13}C spectra of compound 10. 14
Figure S14. ^{1}H and ^{13}C spectra of compound 11a........ 15
Figure S15. ^{1}H and ^{13}C spectra of compound 12a........ 16
Figure S16. ^{1}H and ^{13}C spectra of compound 13. 17
Figure S17. ^{1}H and ^{13}C spectra of compound 14........ 18
Figure S18. ^{1}H and ^{13}C spectra of compound 11b. 19
Figure S19. ^{1}H and ^{13}C spectra of compound 12b. 20
Figure S20. ^{1}H and ^{13}C spectra of compound 15. 21
Figure S21. ^{1}H and ^{13}C spectra of compound 16. 22
Figure S22. ^{1}H and ^{13}C spectra of compound 17. 23
Figure S23. ^{1}H and ^{13}C spectra of compound 11c. 24
Figure S24. ^{1}H and ^{13}C spectra of compound 12c. 25
Figure S25. ^{1}H and ^{13}C spectra of compound 18. 26
Figure S26. ^{1}H and ^{13}C spectra of compound 11d. 27
HPLC TRACES........ 28
Figure S27. HPLC trace of ARM210. 28
Figure S28. HPLC trace of compound 11a. 28
Figure S29. HPLC trace of compound 12a. 29

Figure S30. HPLC trace of compound 11b. 29
Figure S31. HPLC trace of compound 12b. 30
Figure S32. HPLC trace of compound 11c. 30
Figure S33. HPLC trace of compound 12c. 31
Figure S34. HPLC trace of compound 11d. 31
LCMS SPECTRA 32
Figure S35. LCMS spectra of compound 11a. 32
Figure S36. LCMS spectra of compound 11b. 33
Figure S37. LCMS spectra of compound 11c. 34
Figure S38. LCMS spectra of compound 11d. 35
Figure S39. LCMS spectra of compound 12a. 36
Figure S40. LCMS spectra of compound 12c. 37
Notes 38
ABBREVIATIONS 38
REFERENCES 39

SUPPLEMENTARY FIGURES

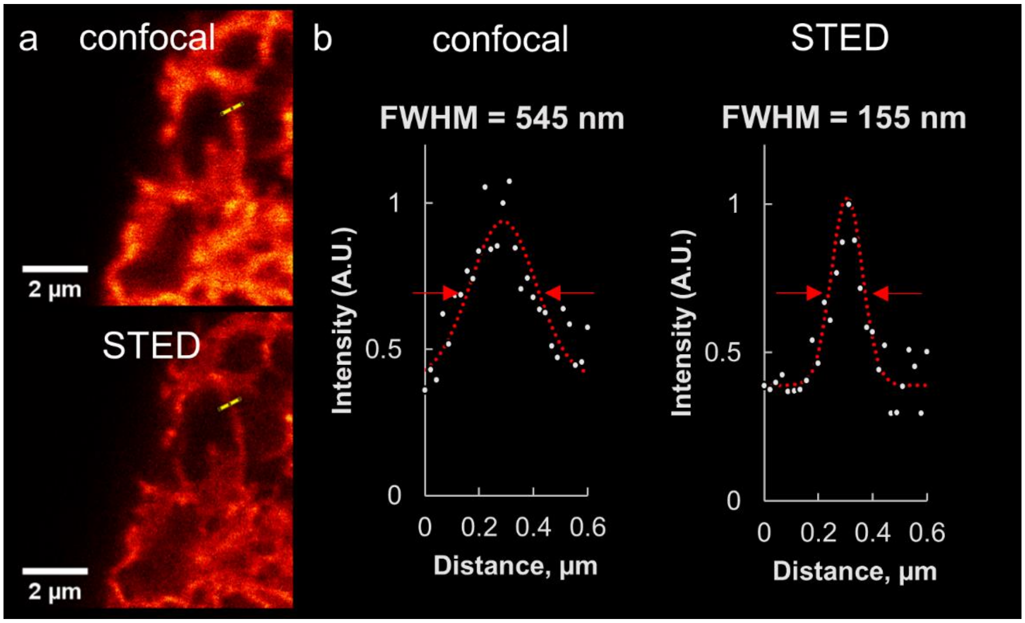

Figure S1. Resolution measurements for R-CEPIA1er in living HEK-293 RyR2 R-CEPIA1er cells. (a) Confocal and STED images were obtained using Abberior STED 775 QUAD scan microscope. (b) The line profiles were taken across the yellow line. Dotted red lines indicate the Gaussian least squares fits used to determine the full width at half-maximum (FWHM) values.

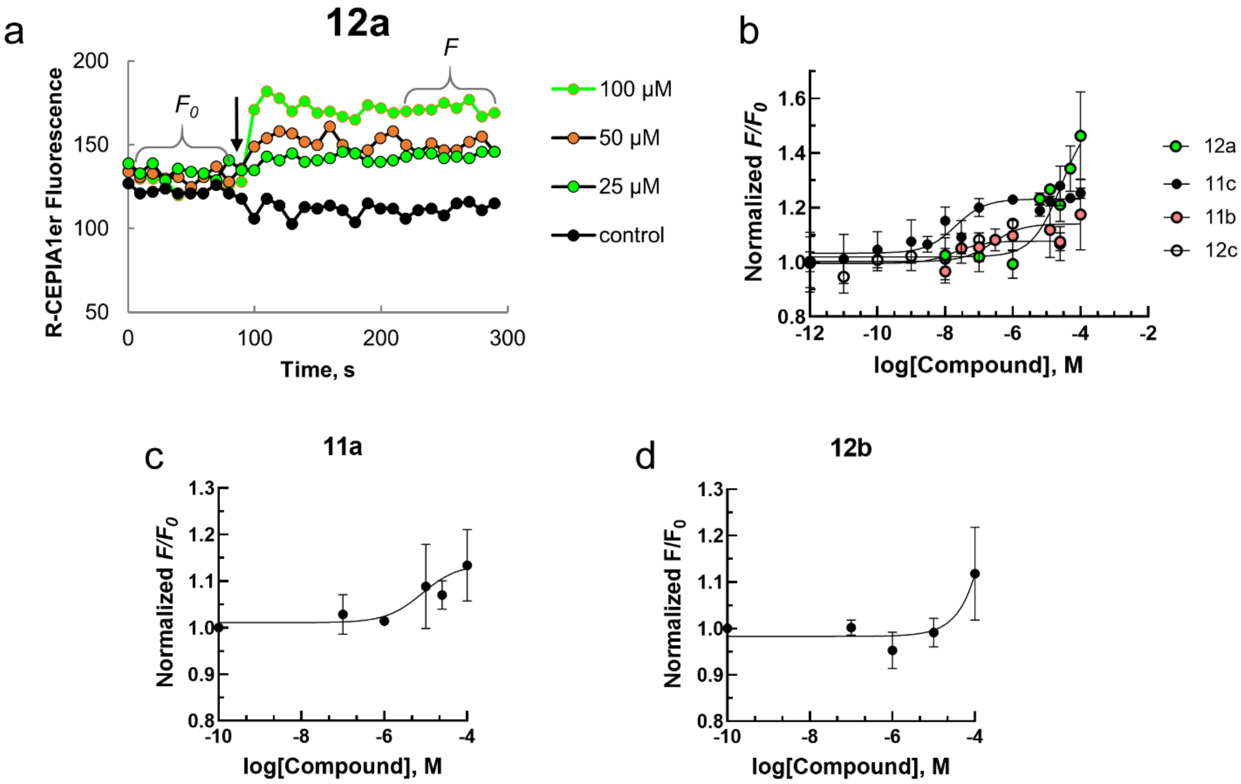

Figure S2. Time-lapse fluorescence measurements using TECAN Spark 20M plate reader with HEK293 cells expressing WT RyR2 and R-CEPIA1er indicator. (a) R-CEPIA1*er* assay, control (0.1 v/v% DMSO) or 100 µM of compound 12a was injected at 90 seconds (arrow). Fluorescence ratio F/F_o, where F_o – average fluorescence for the first 90 seconds, F – average fluorescence for the last 100 s, was taken for the calculations. (b-d) Dose-response effects of compounds 11 and 12 on $[Ca^{2+}]_{ER}$ in HEK293-RyR2-R-CEPIA1*er* cells. Data represent as mean ± SD, n = 8 - 32 independent measurements.

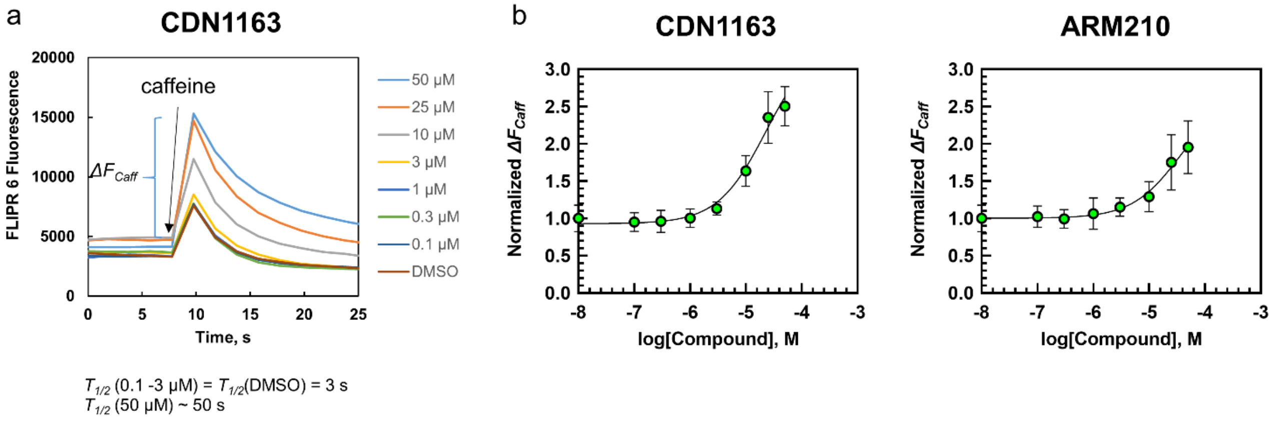

Figure S3. Caffeine-induced Ca^{2+} release assay. (a) HL-1 cells were incubated with FLIPR 6 Ca^{2+} indicator and then treated with various concentrations of CDN1163. Ca^{2+} influx was initiated by the addition of 10 mM caffeine. The differences between the caffeine induced peak minus basal fluorescence, ΔF_{Caff}, were taken for the analysys. (b) Dose-response curves of CDN1163 and ARM210. The data were normalized to the 0.1 v/v% DMSO. Values represent the mean ± S.D., n = 8.

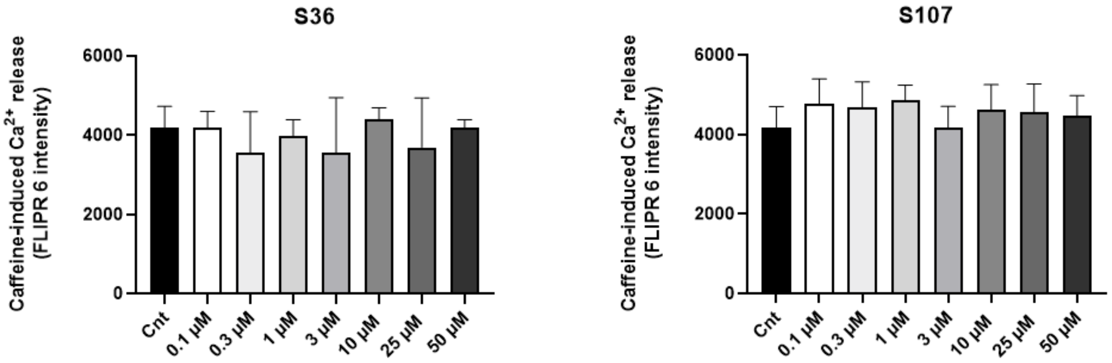

Figure S4. S36 and S107 have shown no concentration dependent effect in caffeine-induced Ca^{2+} release assay, n = 8.

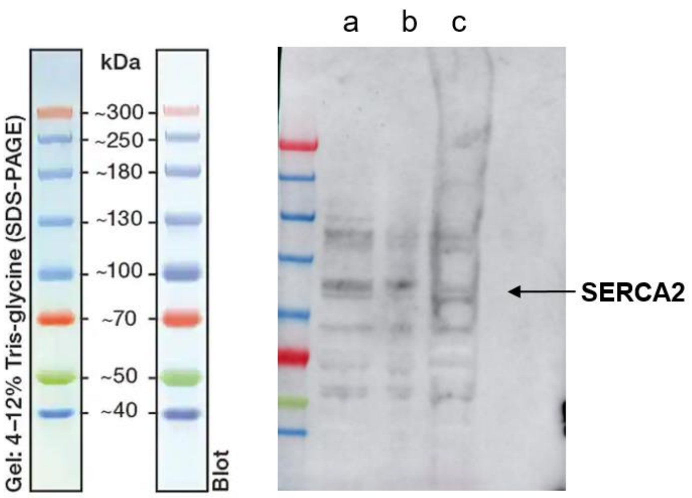

Figure S5. Western blot analysis of HEK293 ER-vesicles. (a) Supernatant after 10000 × g centrifugation, (b) supernatant after 100000 × g centrifugation, (c) pellet after 100000 × g centrifugation.

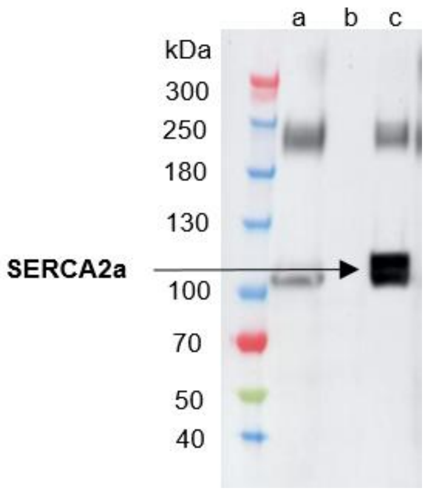

Figure S6. Western blot analysis of mouse ventricular microsomes. (a) Supernatant after 10000 × g centrifugation, (b) supernatant after 100000 × g centrifugation, (c) pellet after 100000 × g centrifugation.

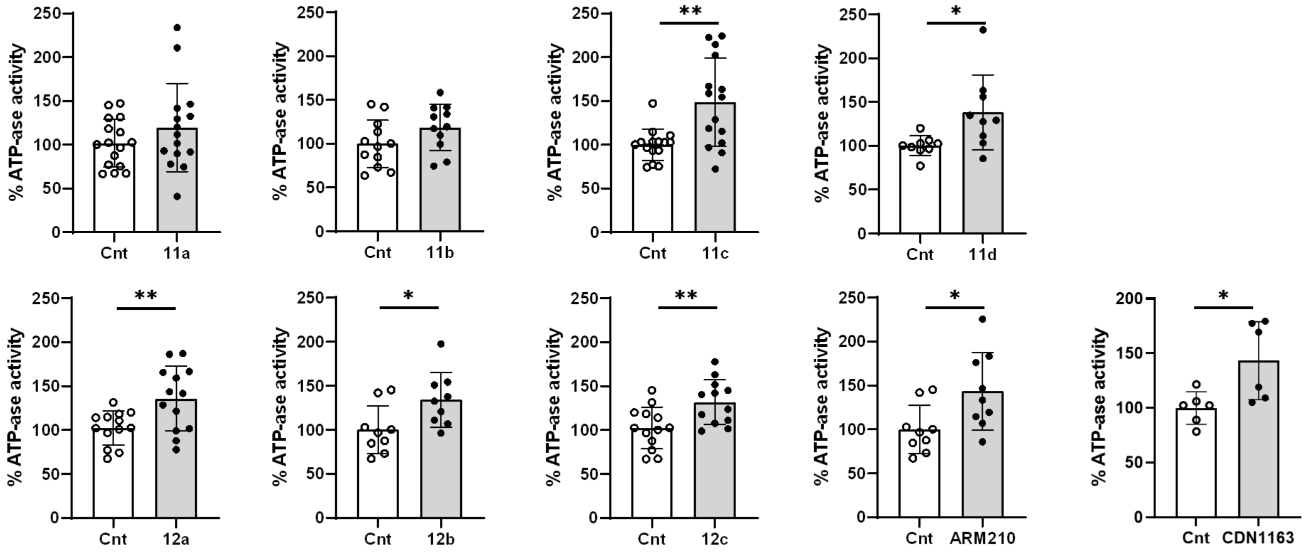

Figure S7. SERCA2 activity measurements on microsomal membrane vesicles derived from HEK-293T cells. Effect of 10 µM **11**, **12** (1 µM for 12b), **ARM210** and **CDN1163** on SERCA2 activity. The rate of ATP consumption was normalised to the 0.1 v/v% DMSO. Values represent the mean ± S.D., * $P < 0.05$ vs. control (0.1 % DMSO) by unpaired t test; n = 6 - 14.

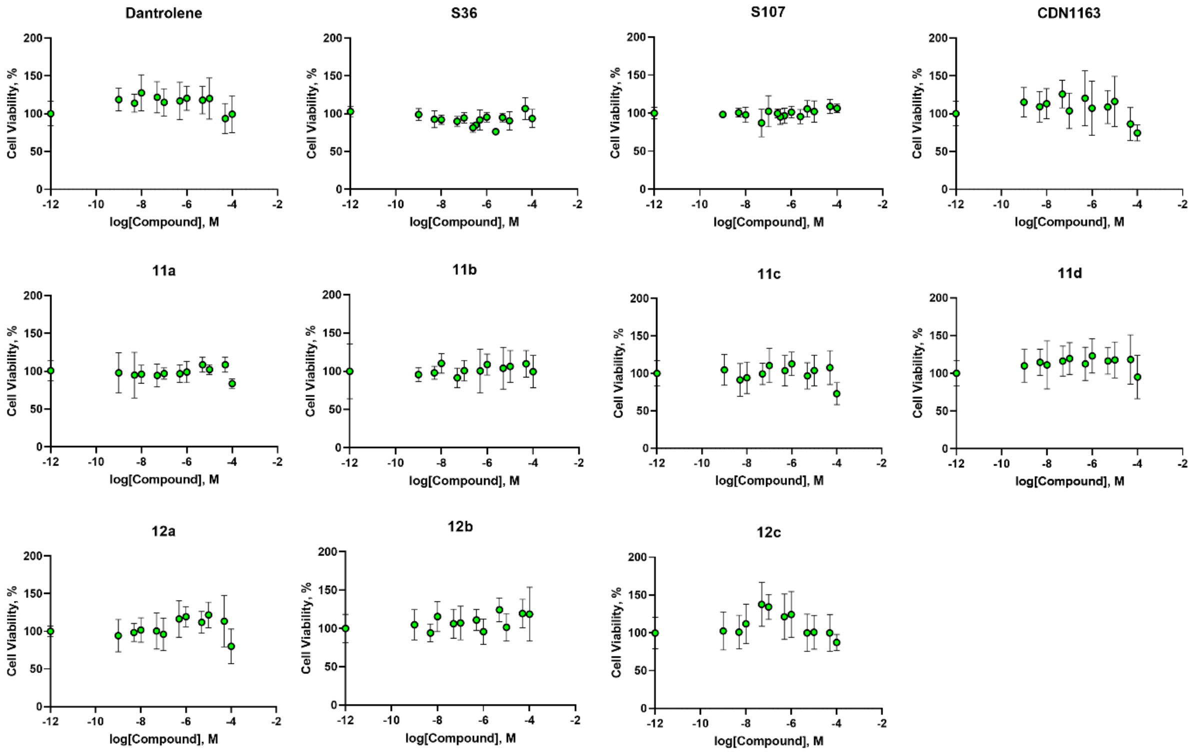

Figure S8. Cell viability after 24-hour incubation with test compounds. HL cells were treated with various concentrations of the tested compounds (24 h) and assayed using CytoTox-Glo™ Cytotoxicity kit. The effect from non-treated cells (incubation in DMEM for 24 hours) was taken as a 100% cell viability. Data represent as mean ± SD, n = 3 – 6 independent measurements.

NMR SPECTRA

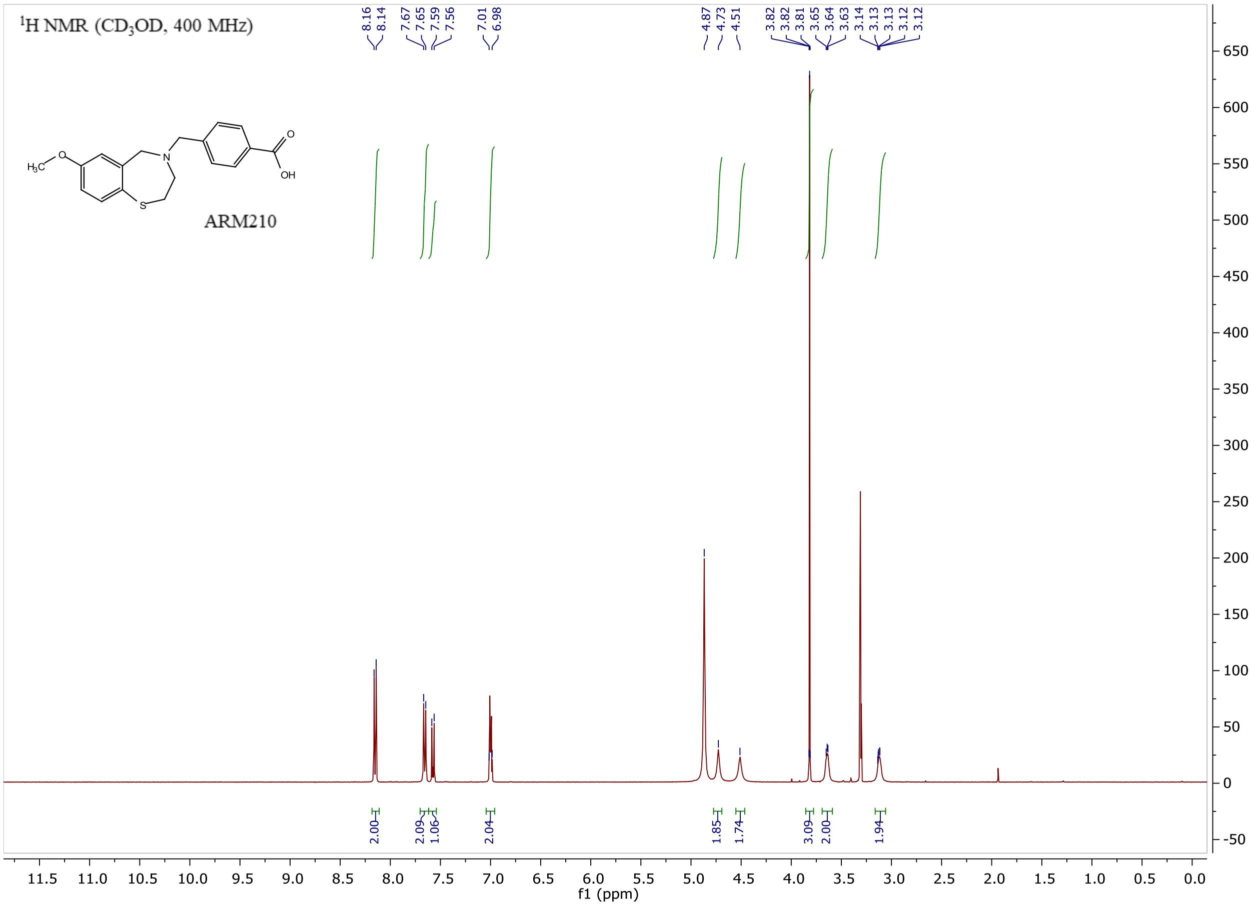

Figure S9. ^{1}H spectrum of ARM210.

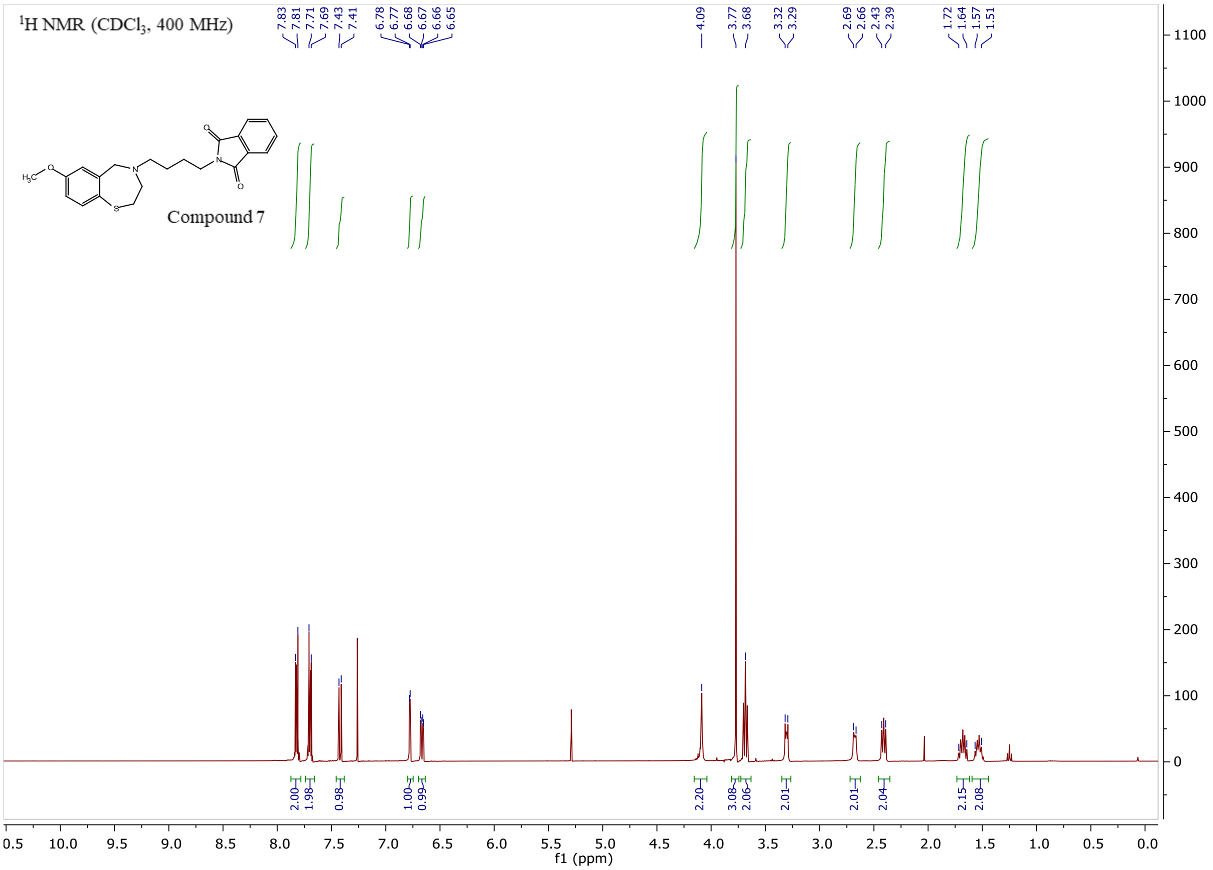

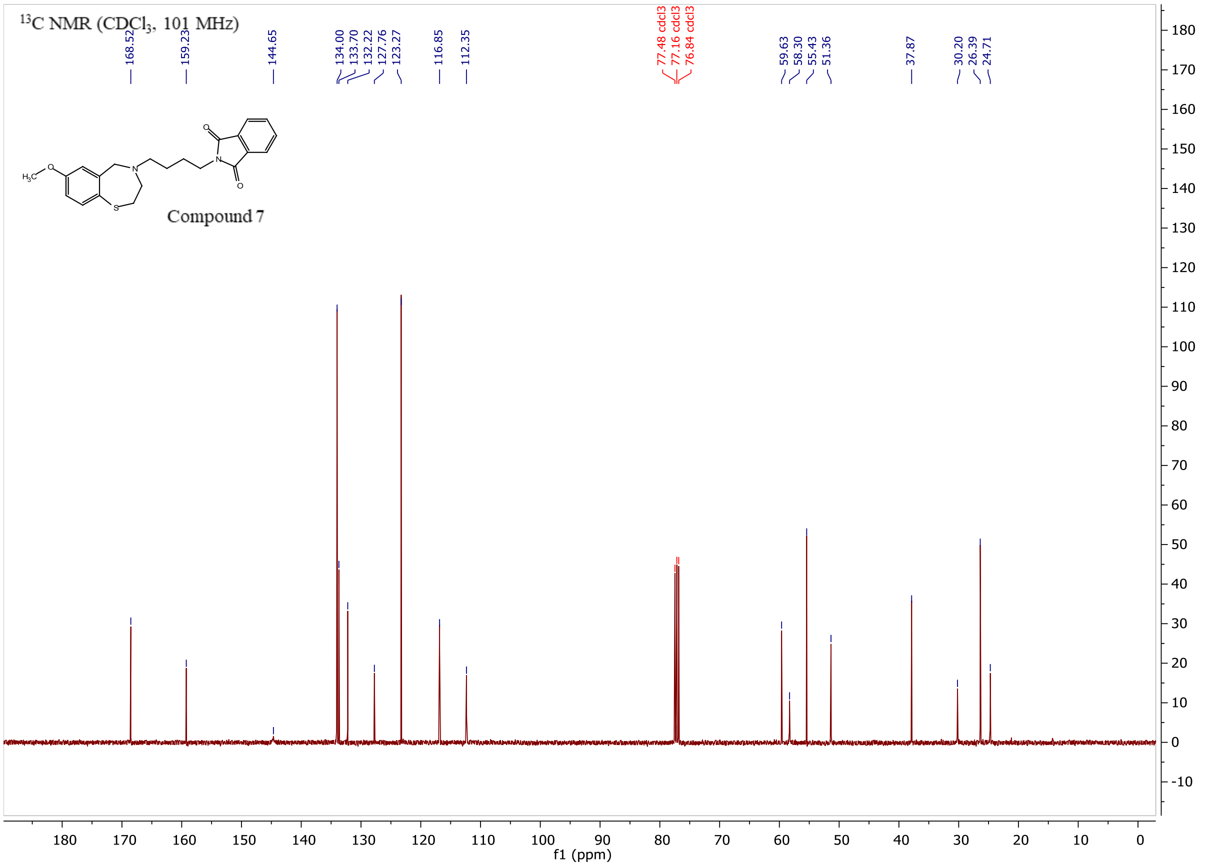

Figure S10. ¹H and ¹³C spectra of compound 7.

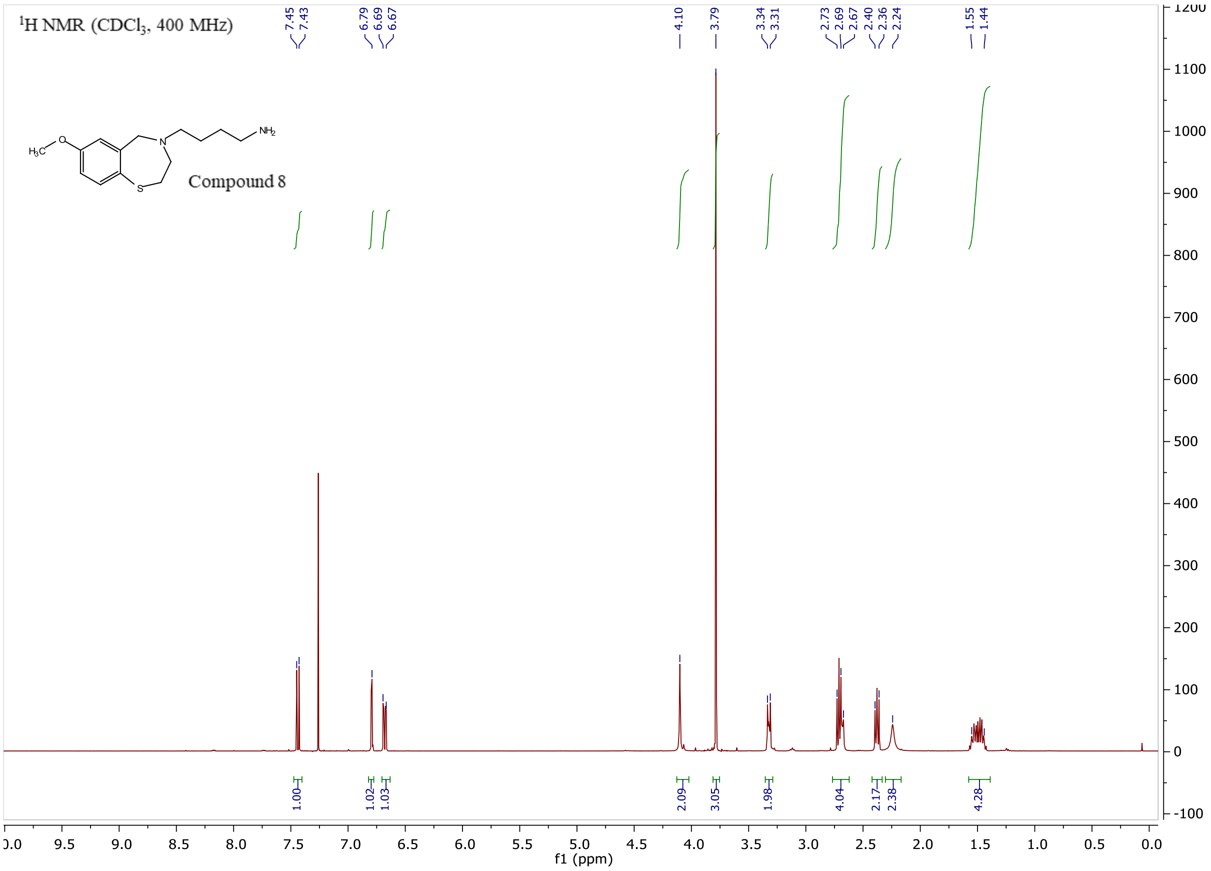

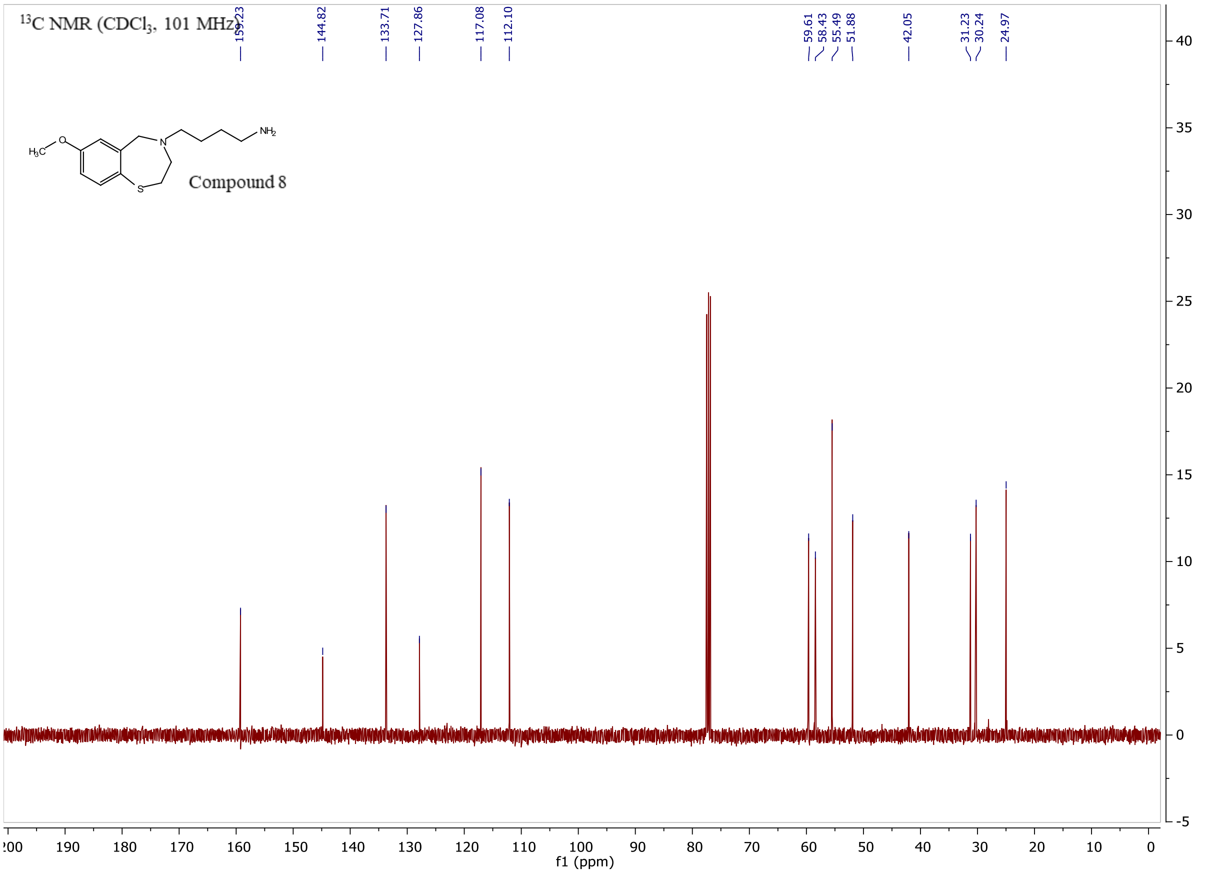

Figure S11. ^{1}H and ^{13}C spectra of compound 8.

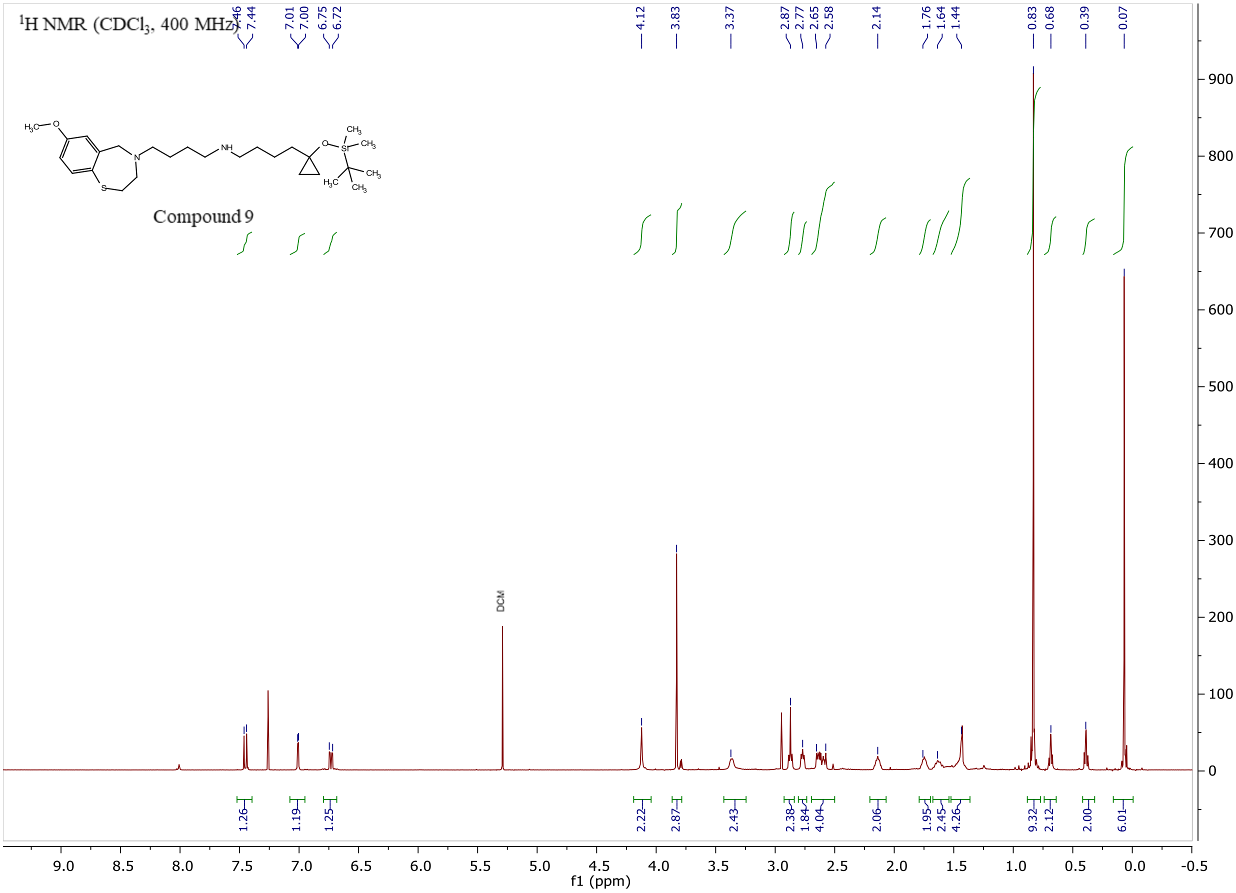

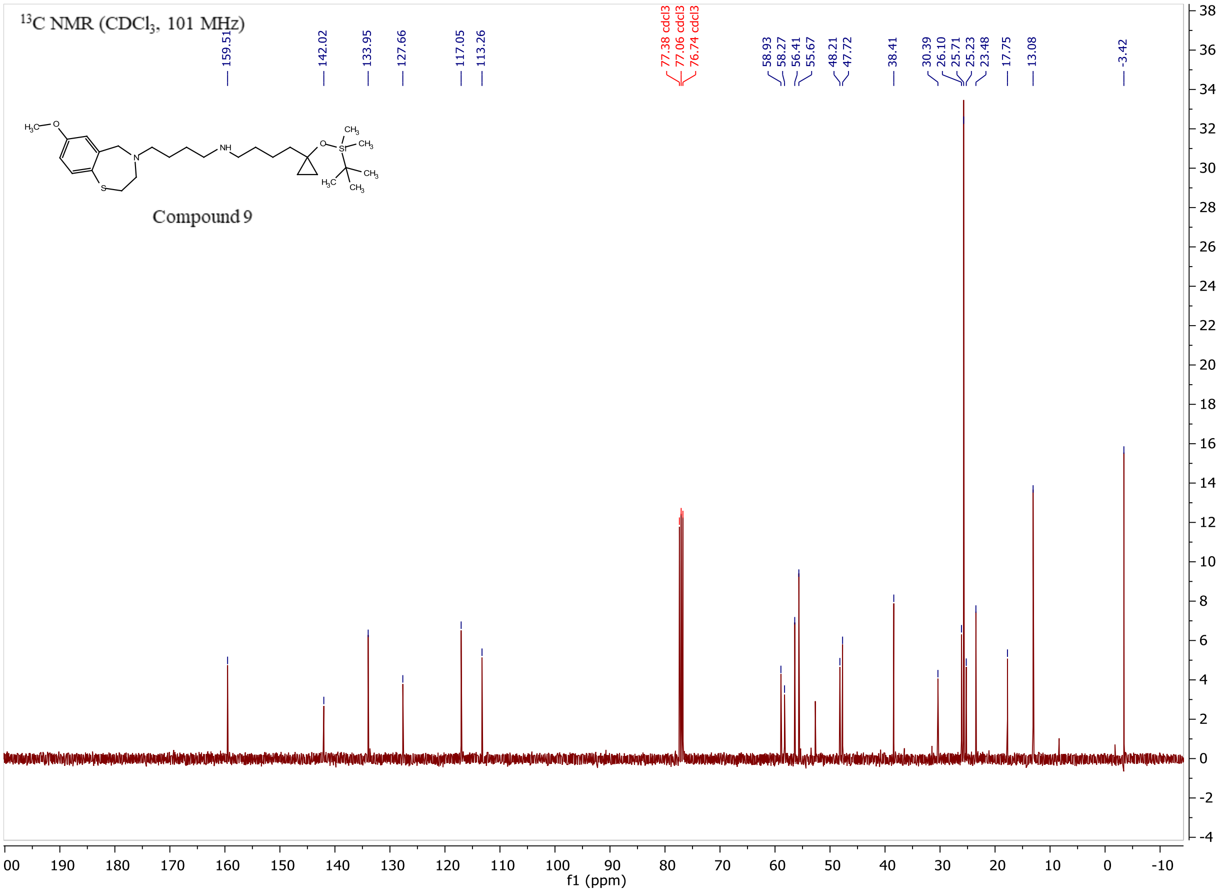

Figure S12. ^{1}H **and** ^{13}C **spectra of compound 9.**

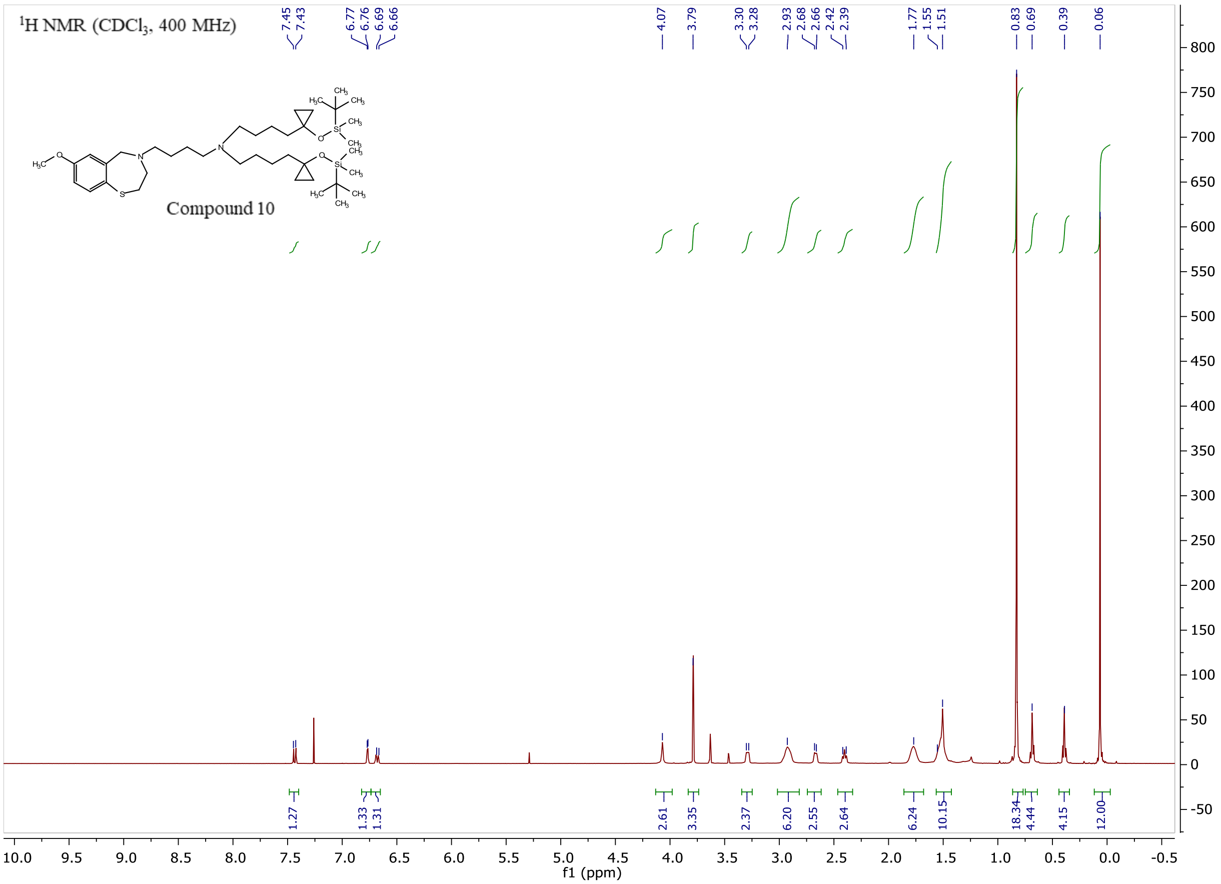

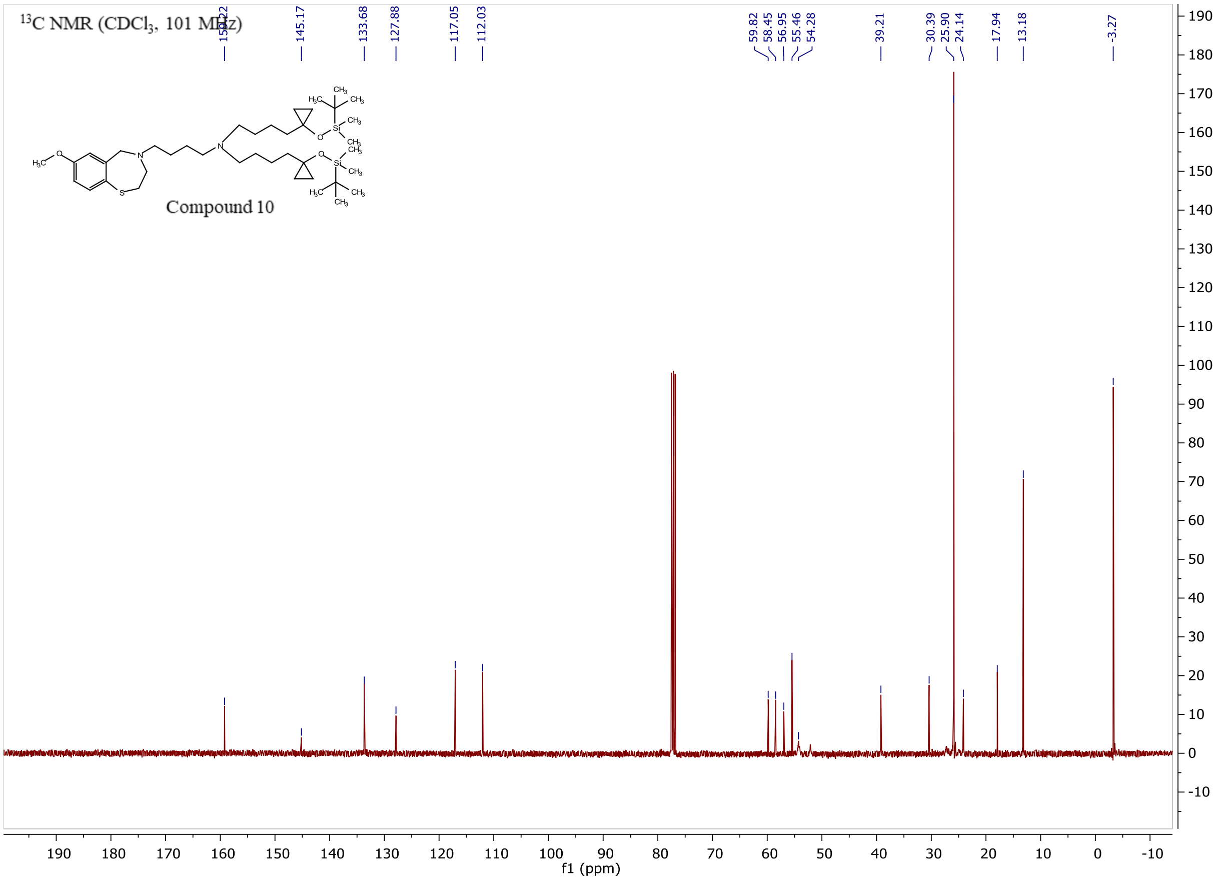

Figure S13. ¹H and ¹³C spectra of compound 10.

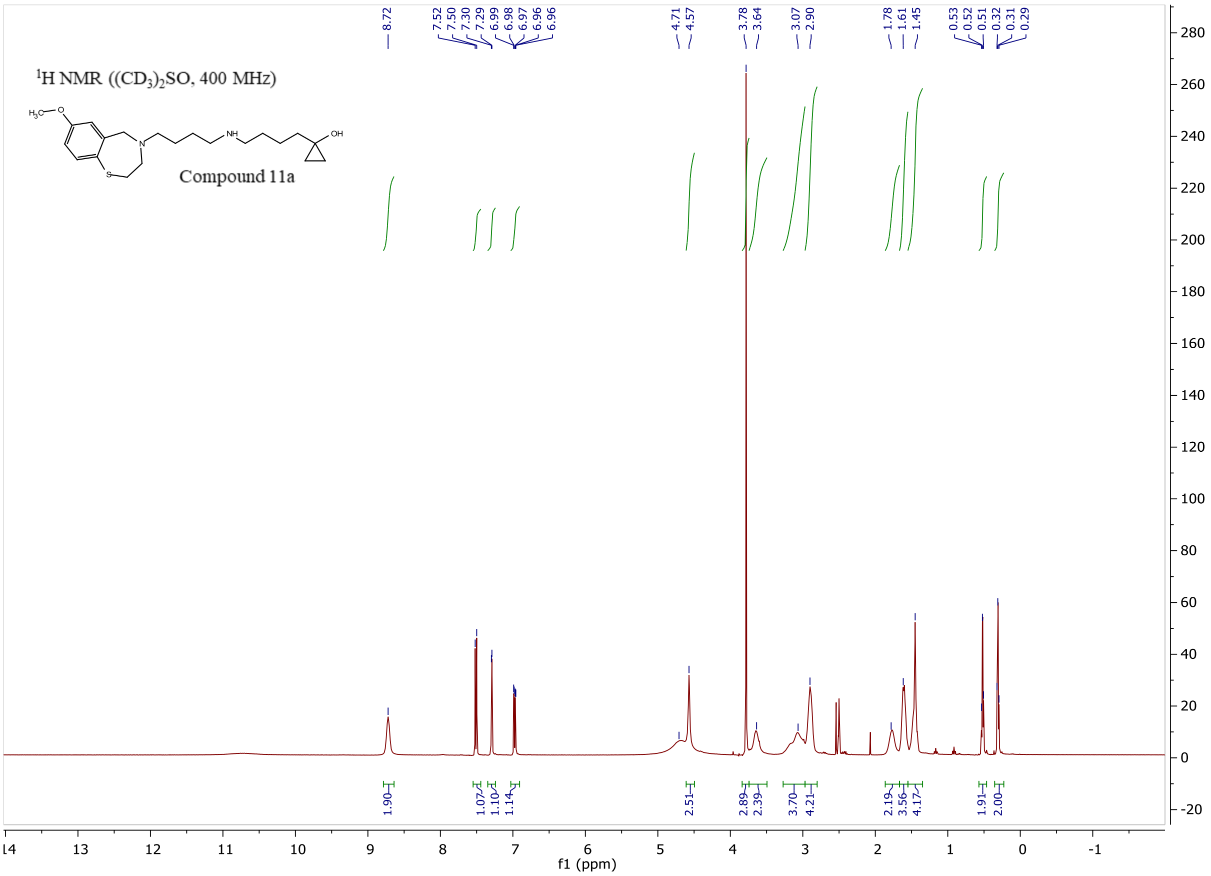

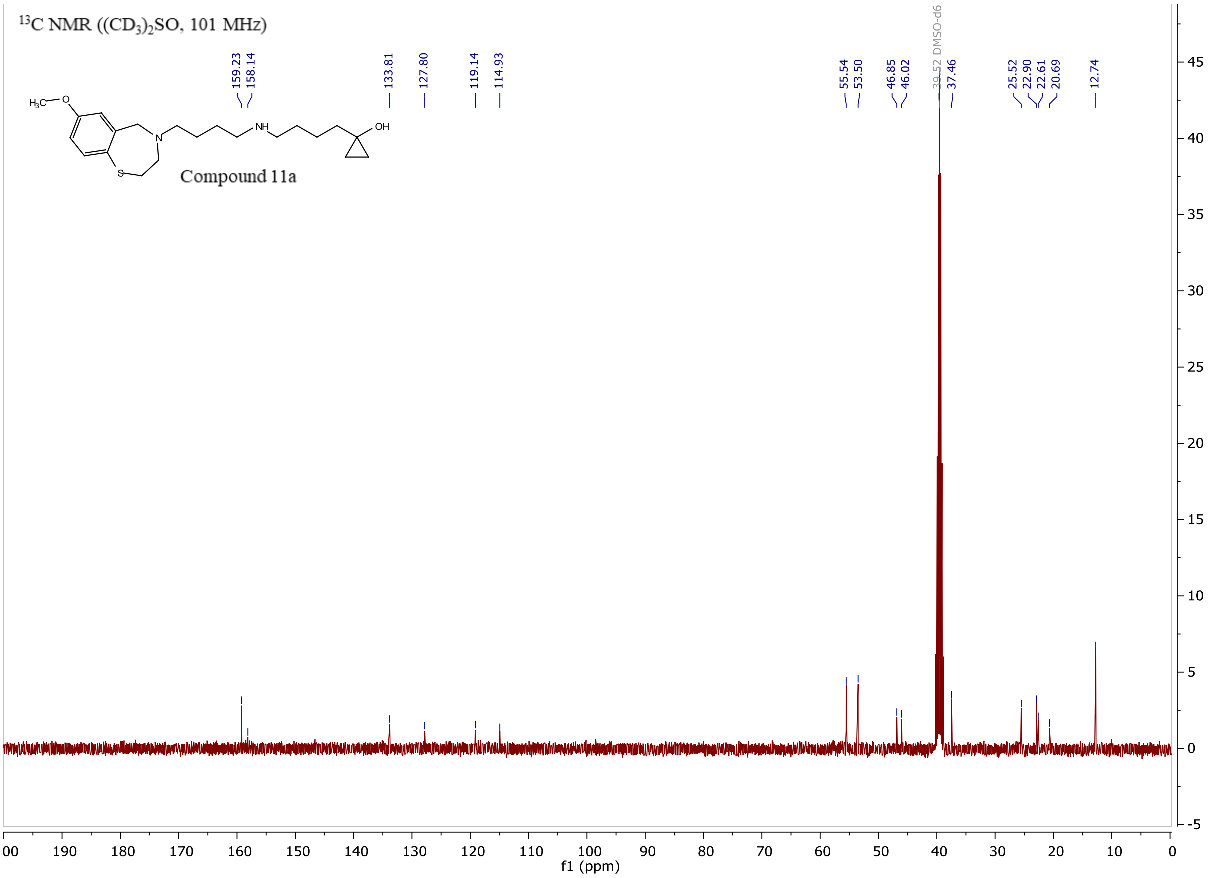

Figure S14. ^{1}H and ^{13}C spectra of compound **11a**.

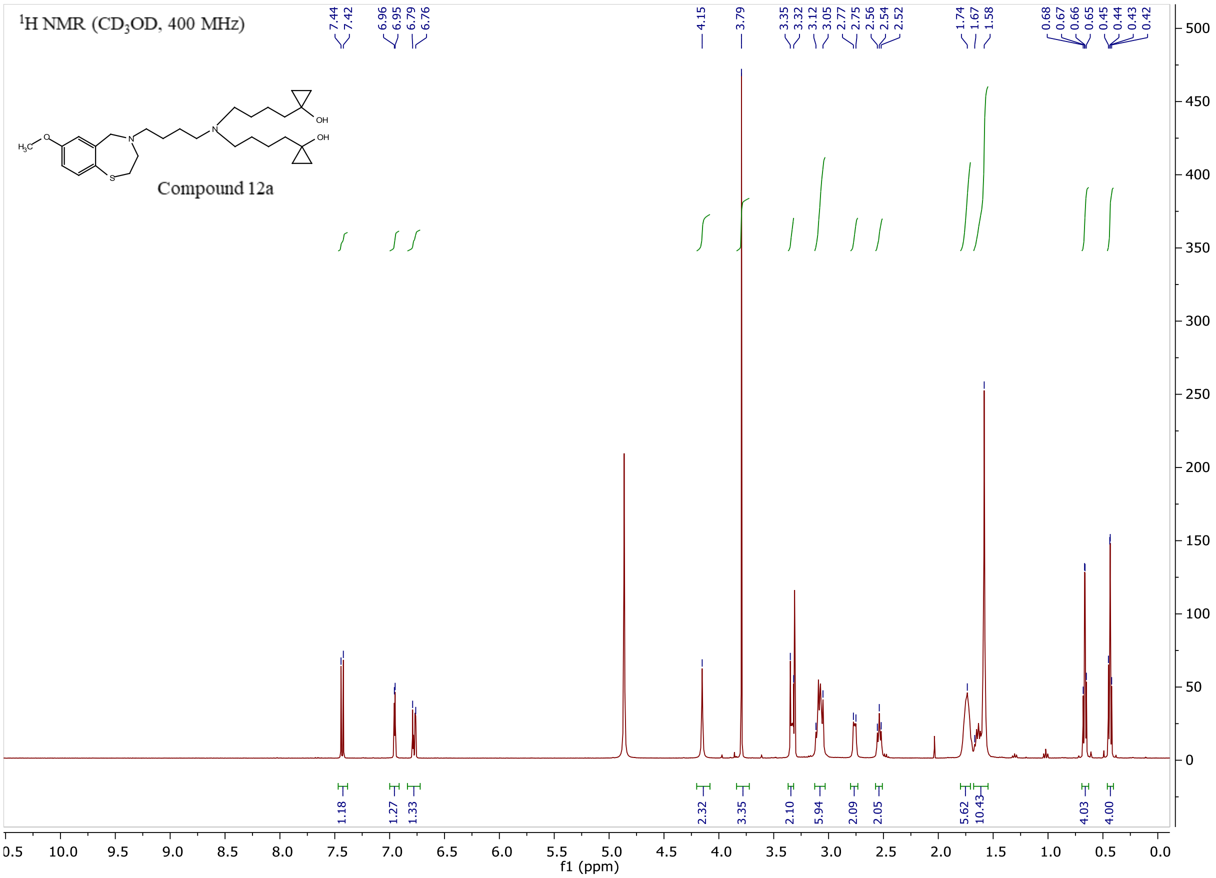

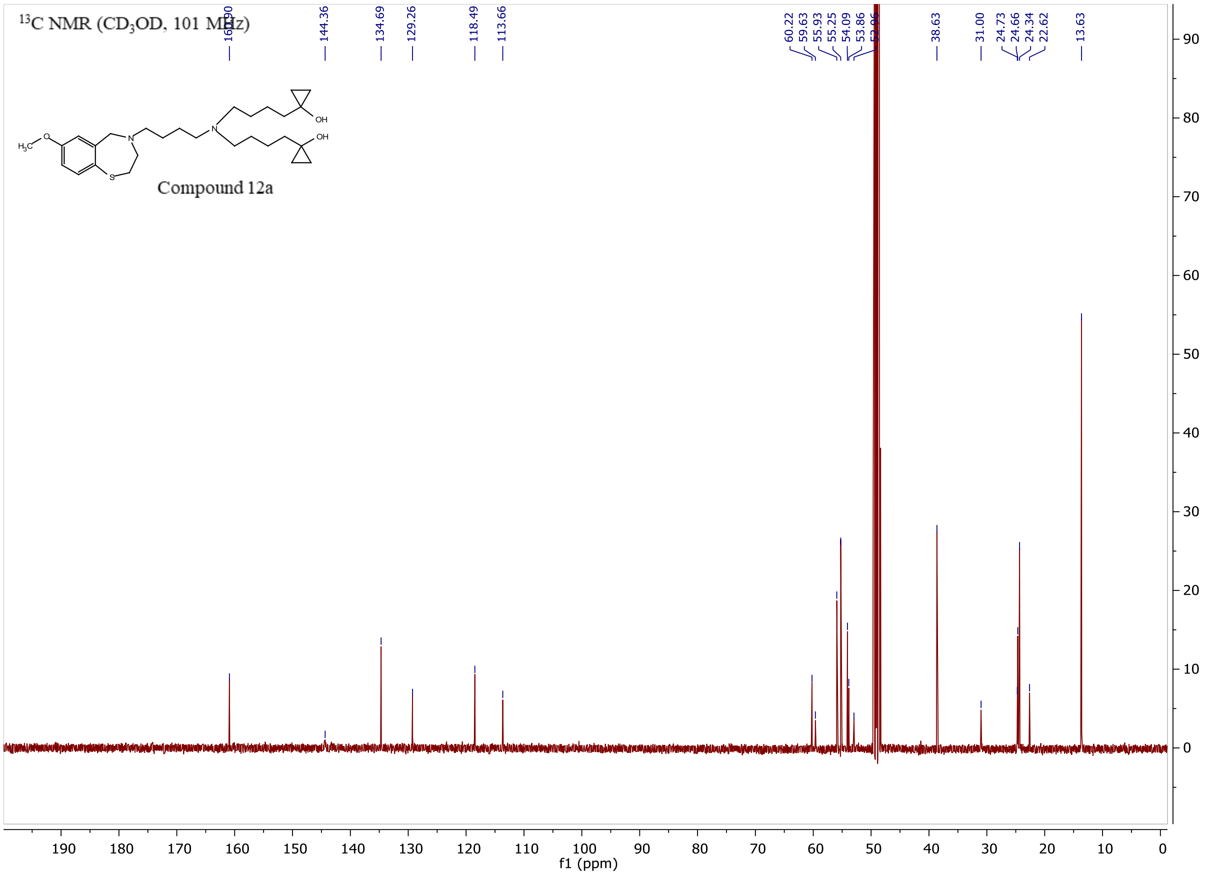

Figure S15. ^{1}H **and** ^{13}C **spectra of compound 12a.**

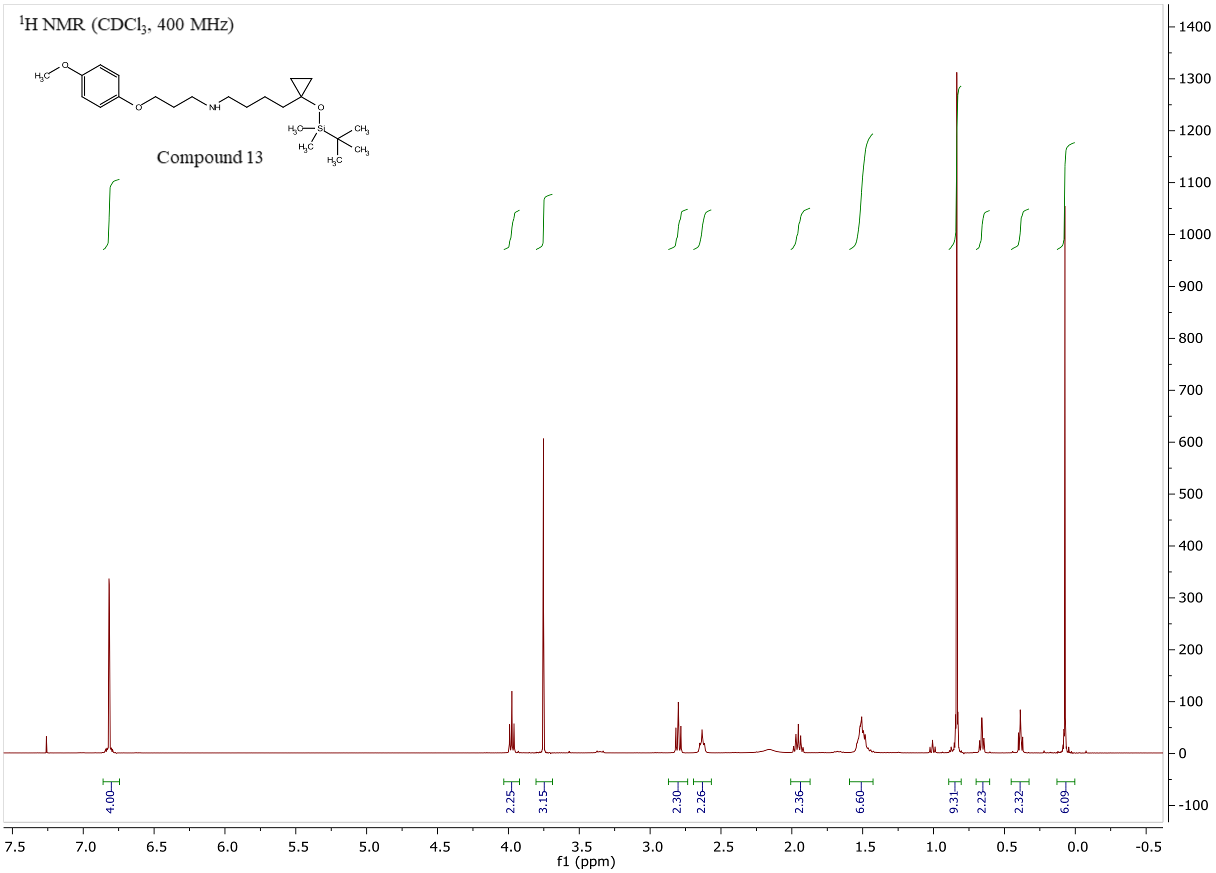

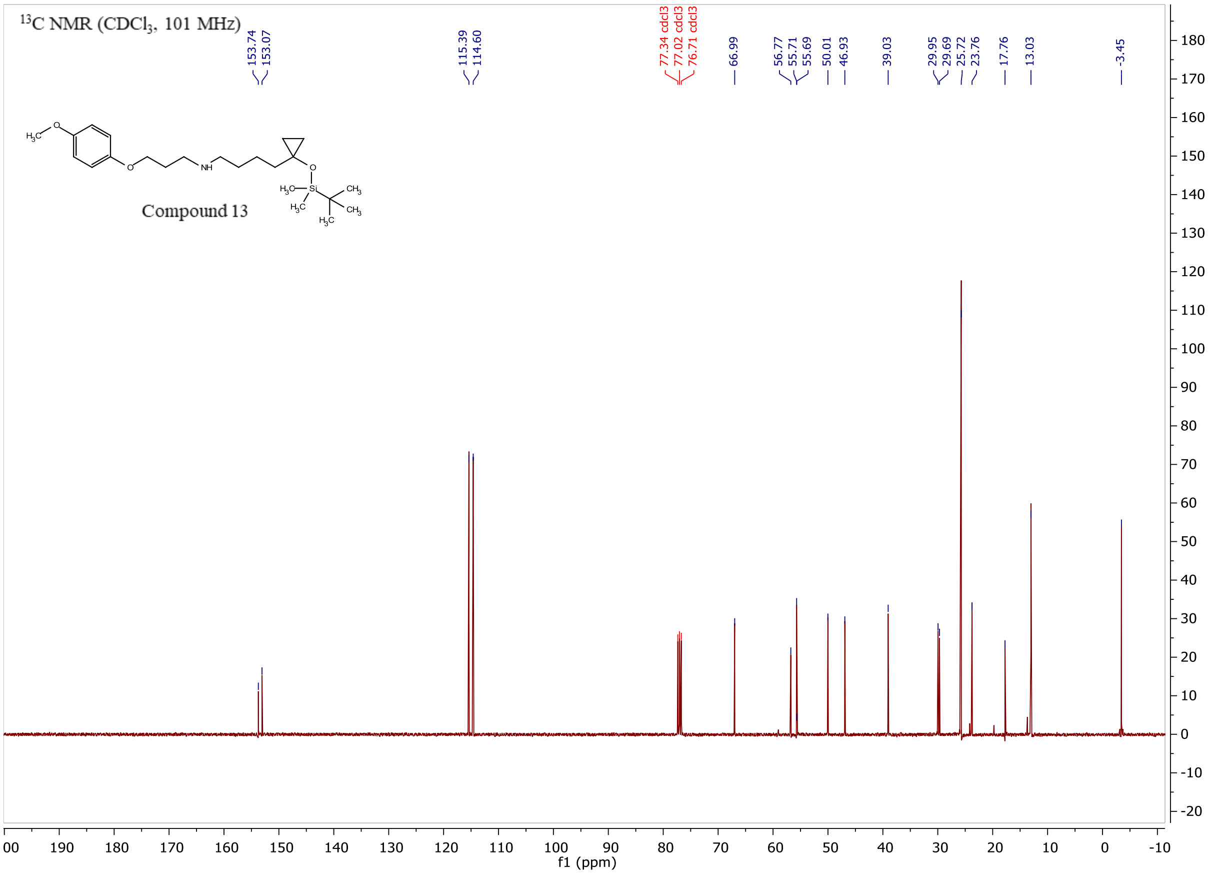

Figure S16. ¹H and ¹³C spectra of compound 13.

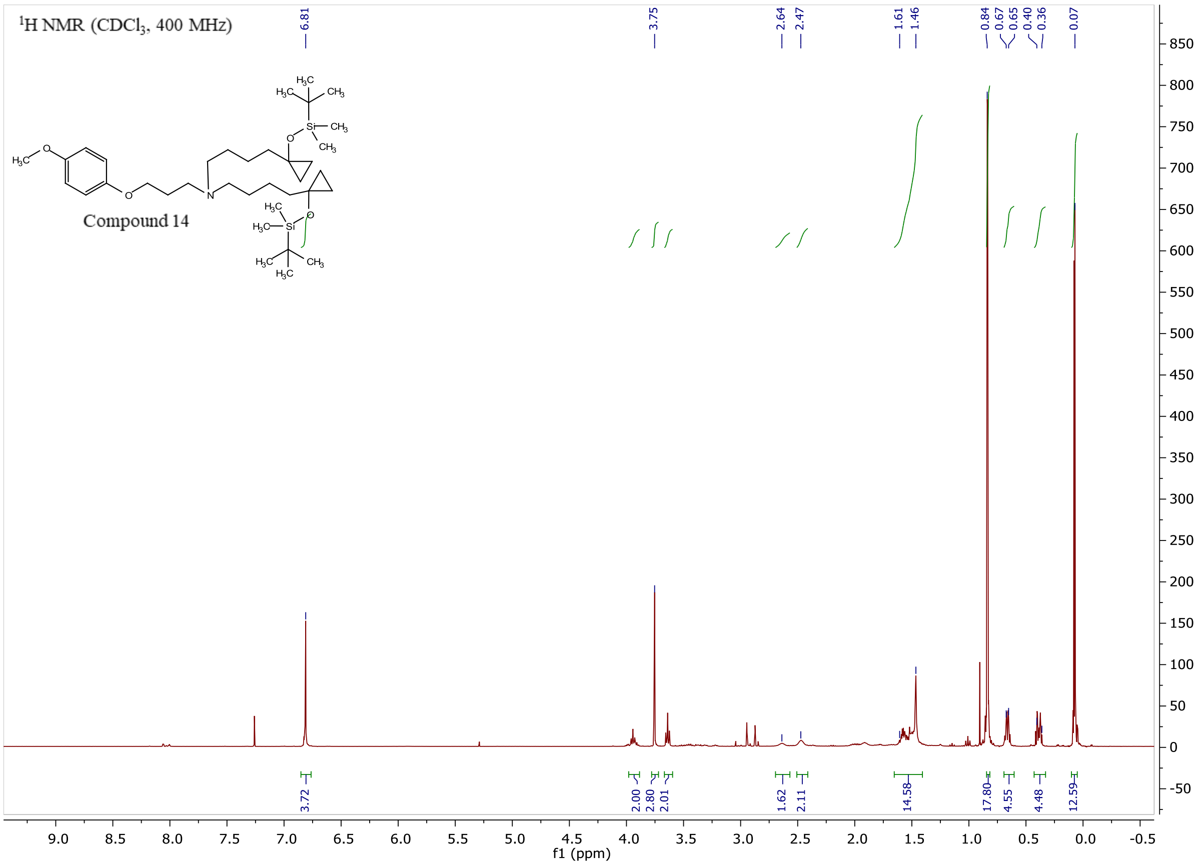

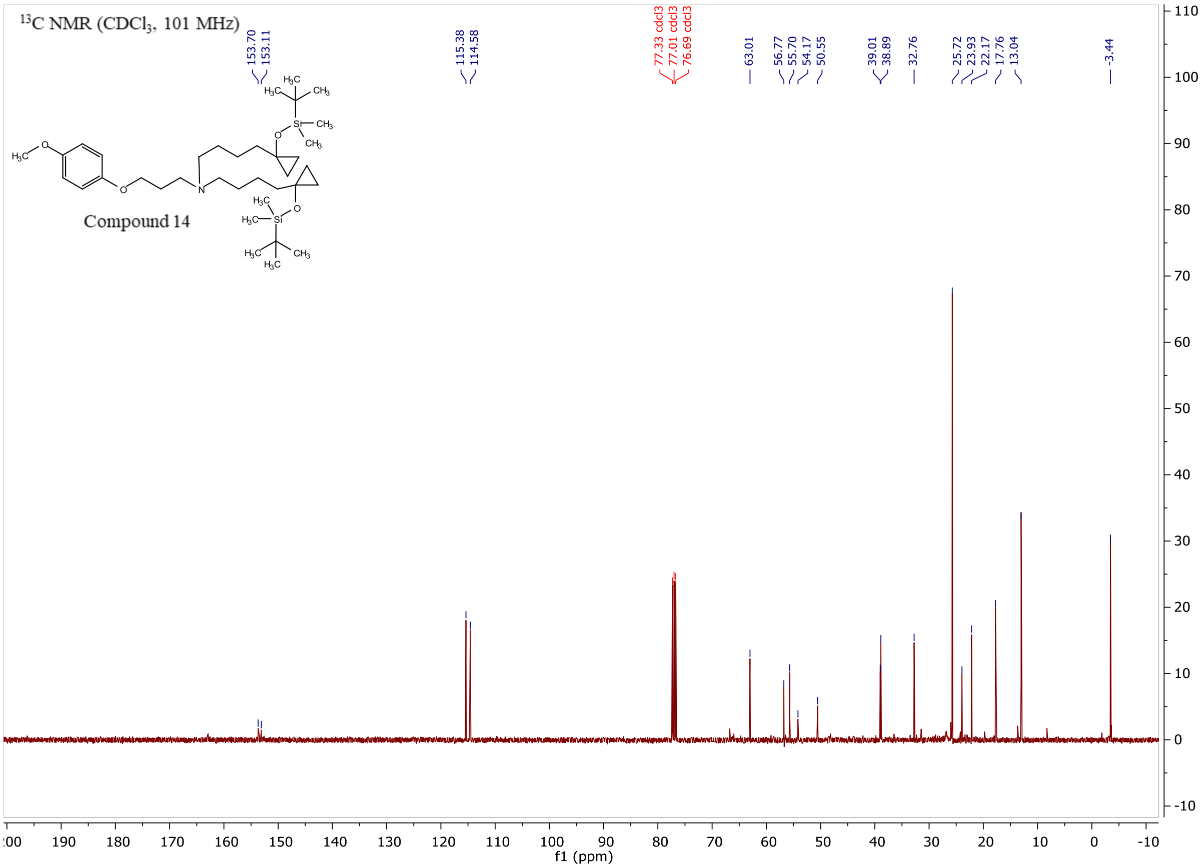

Figure S17. ^{1}H and ^{13}C spectra of compound **14**.

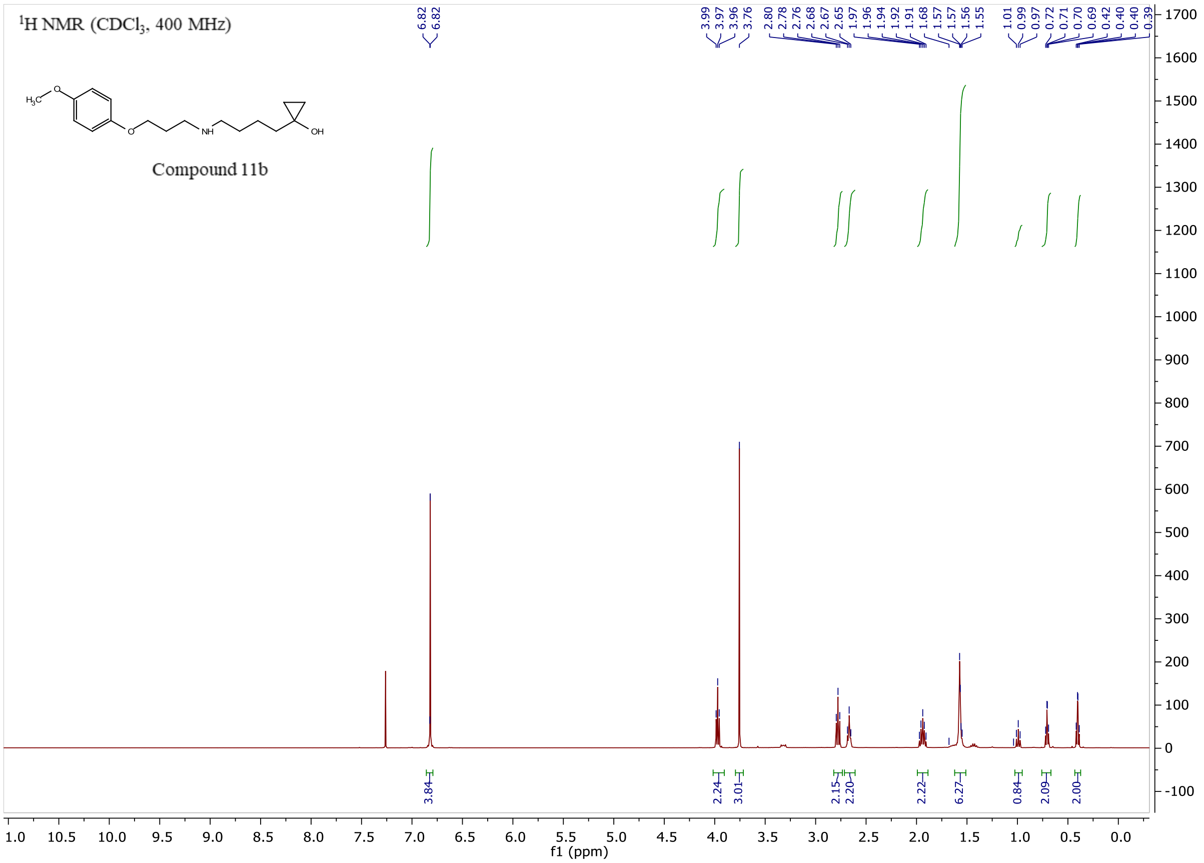

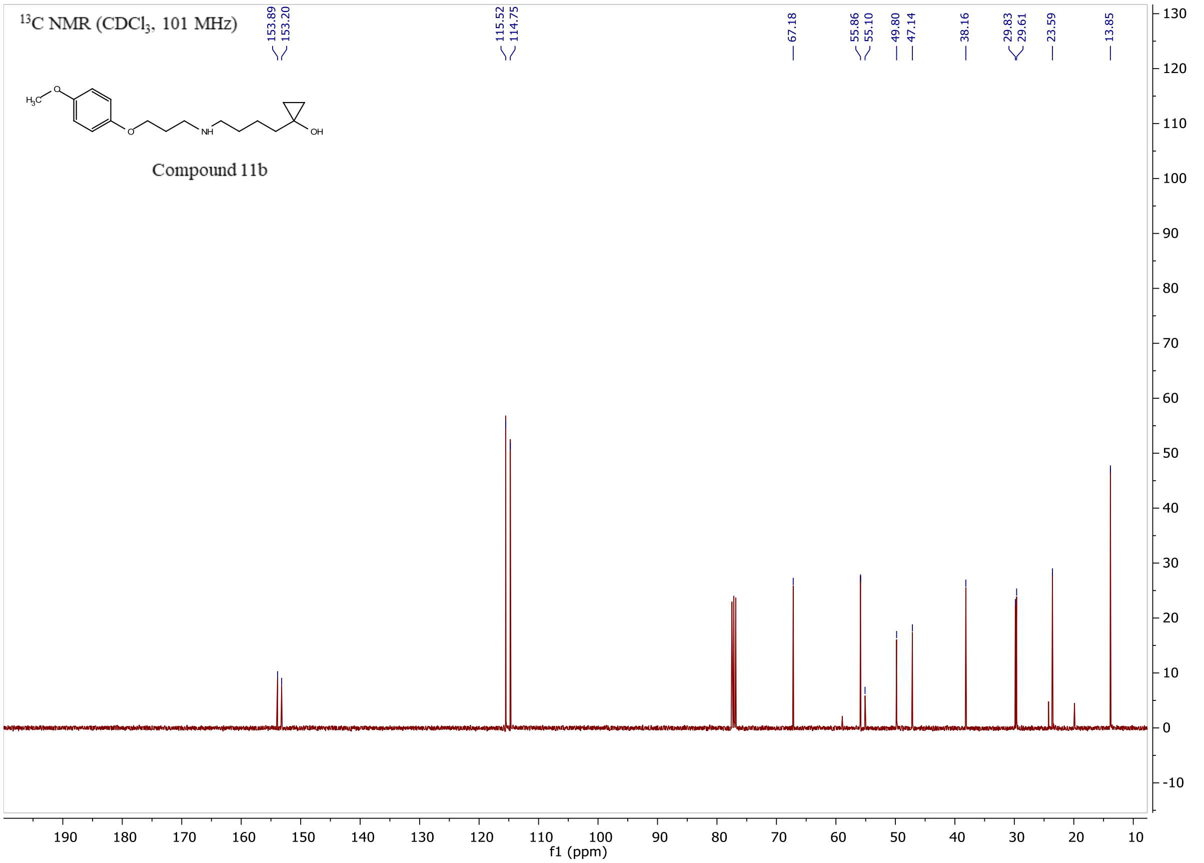

Figure S18. **^{1}H and ^{13}C spectra of compound 11b.**

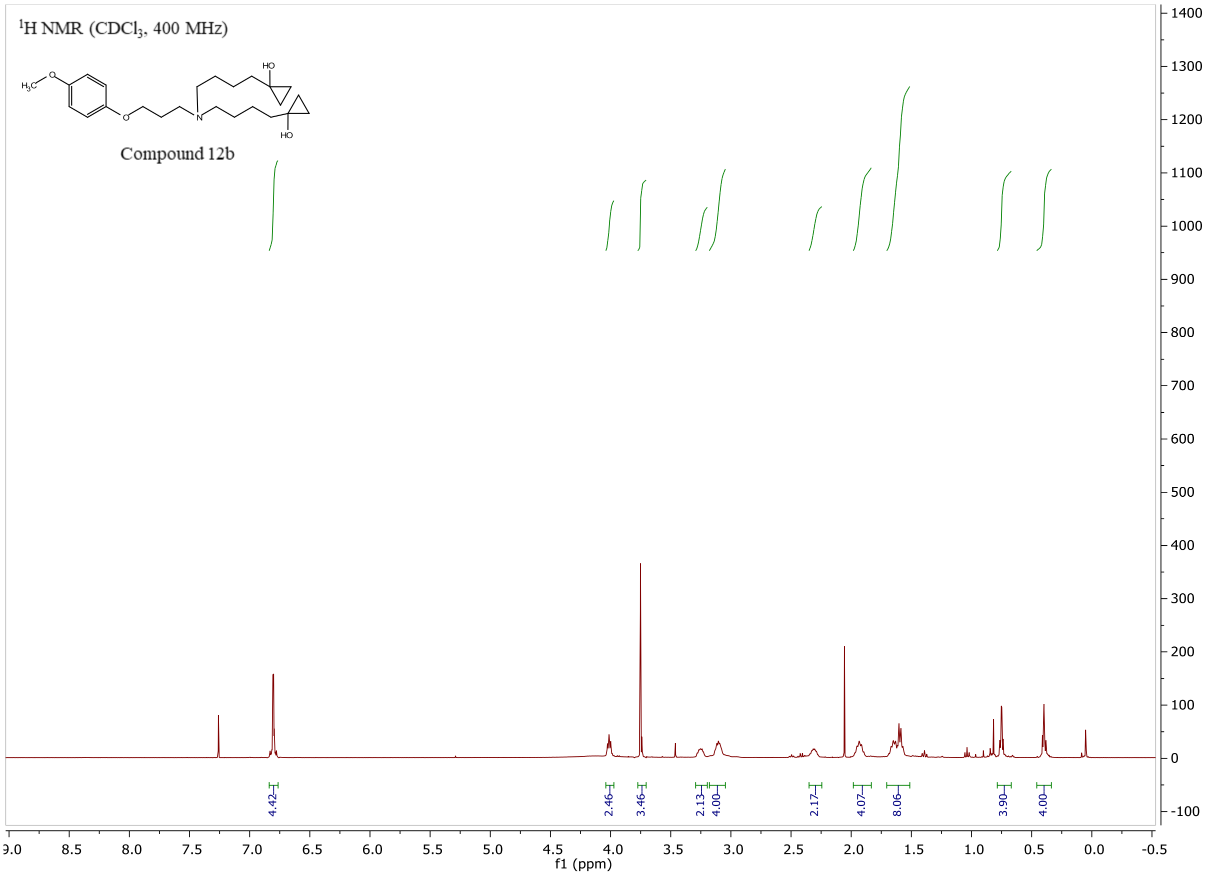

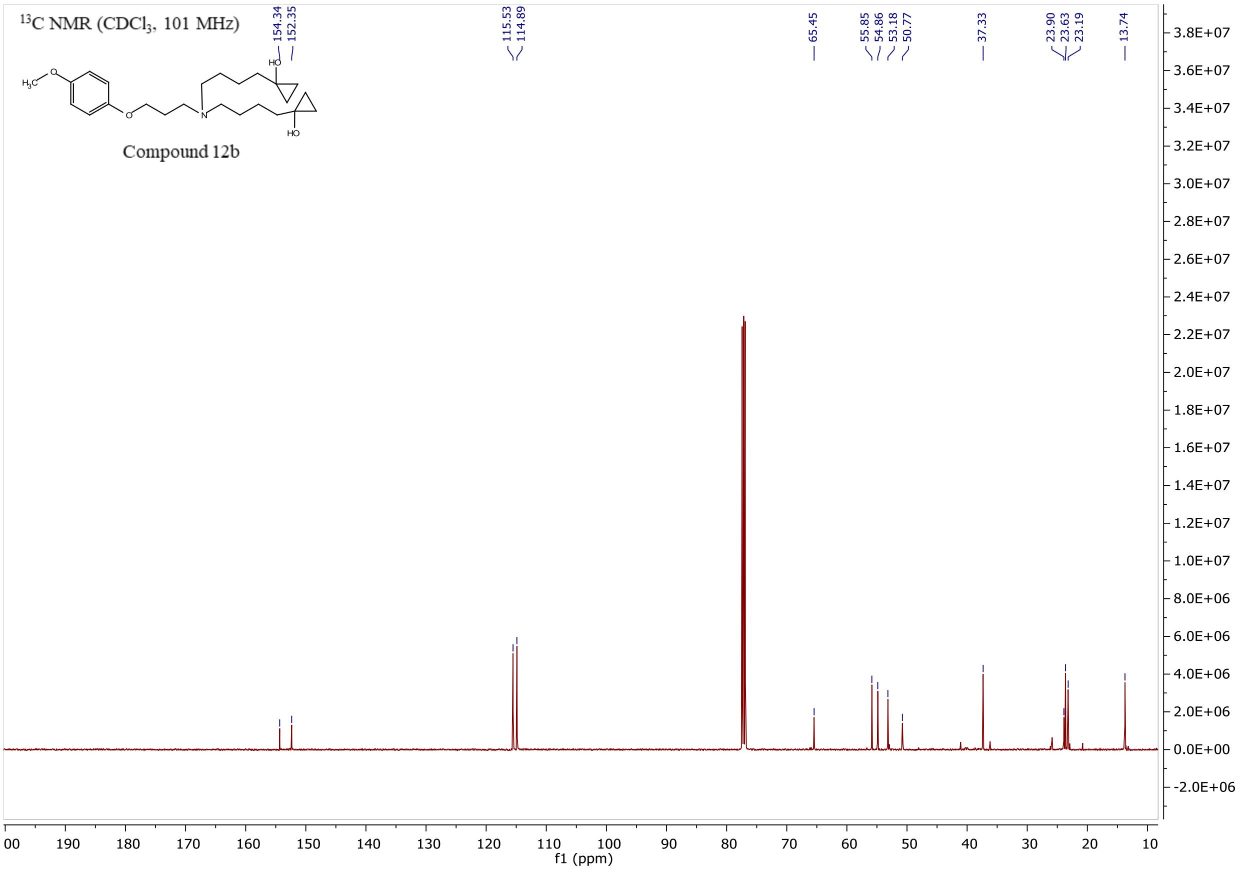

Figure S19. ^{1}H and ^{13}C spectra of compound 12b.

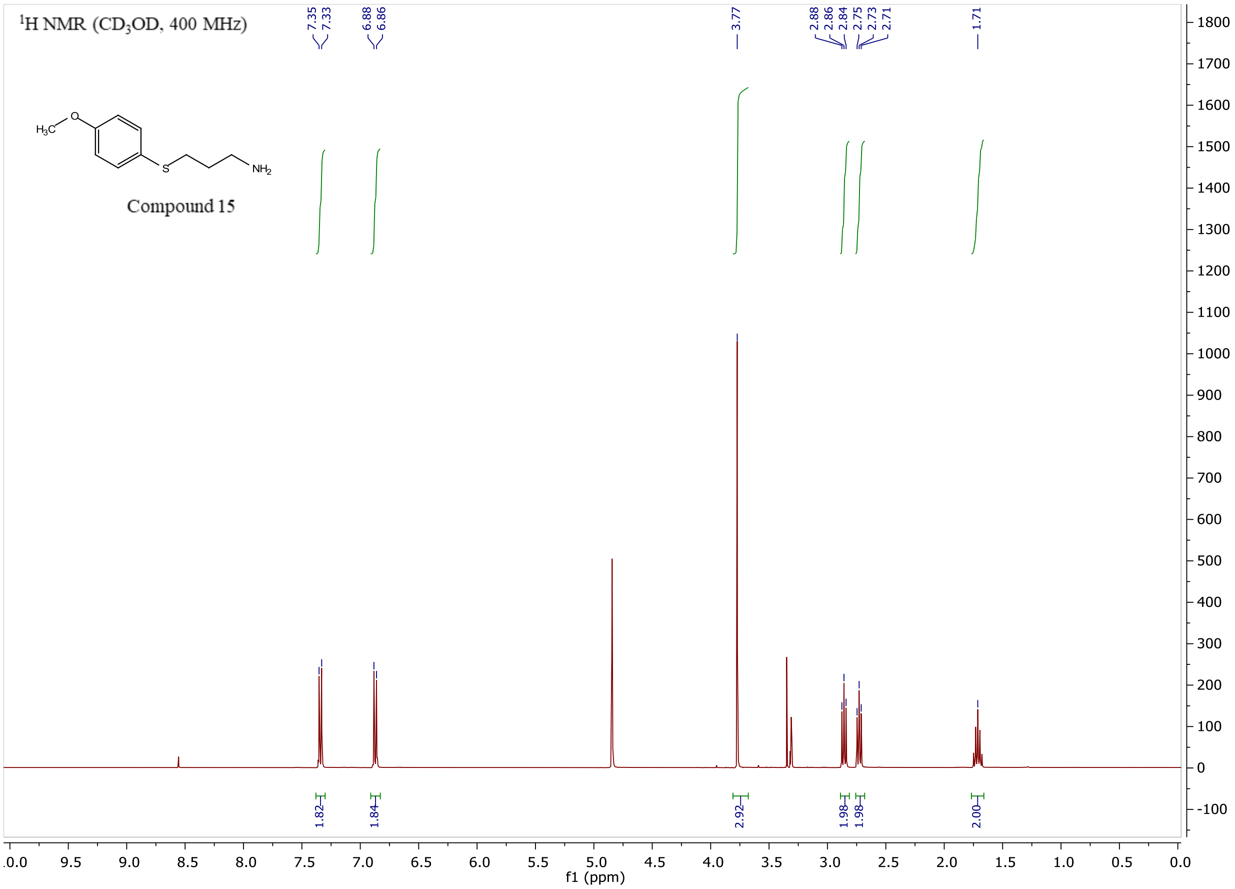

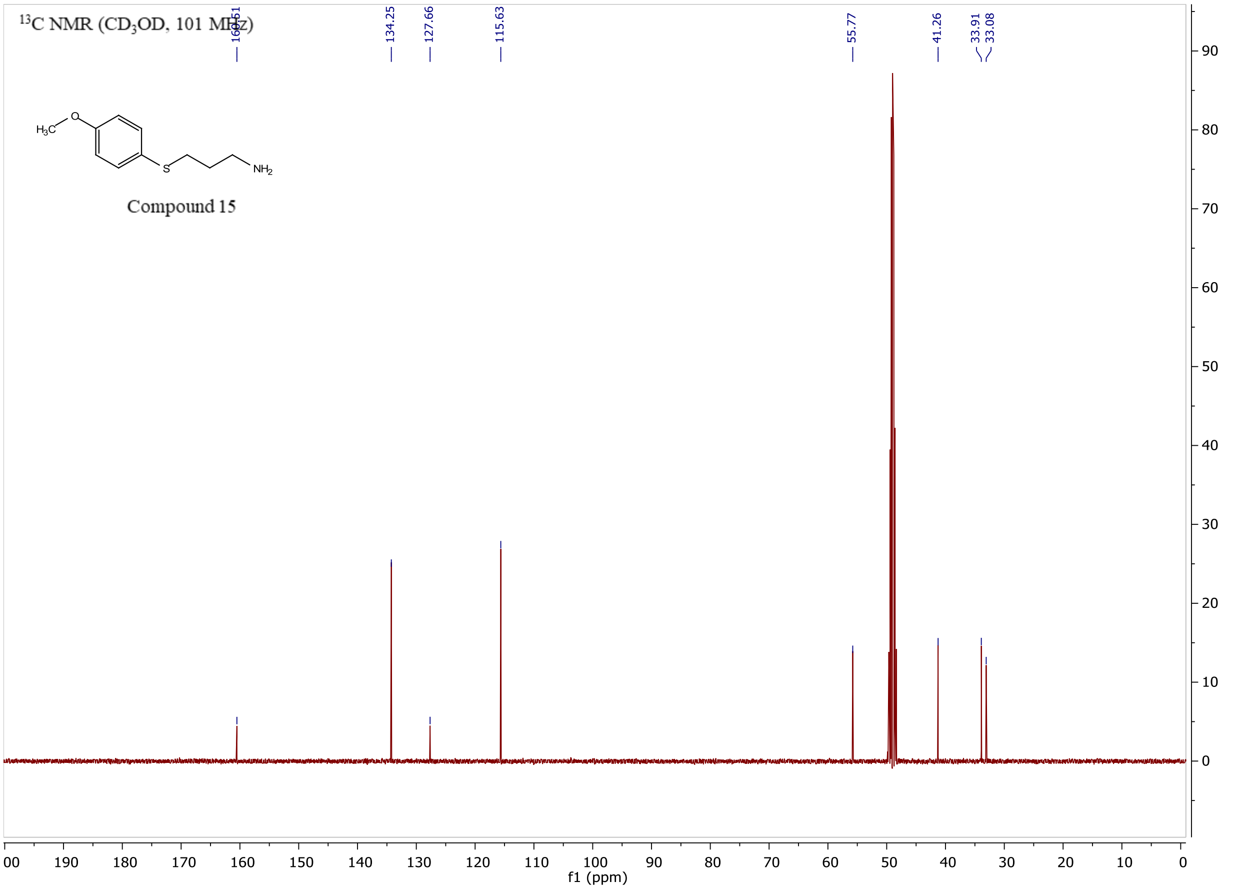

Figure S20. ^{1}**H and** ^{13}**C spectra of compound 15.**

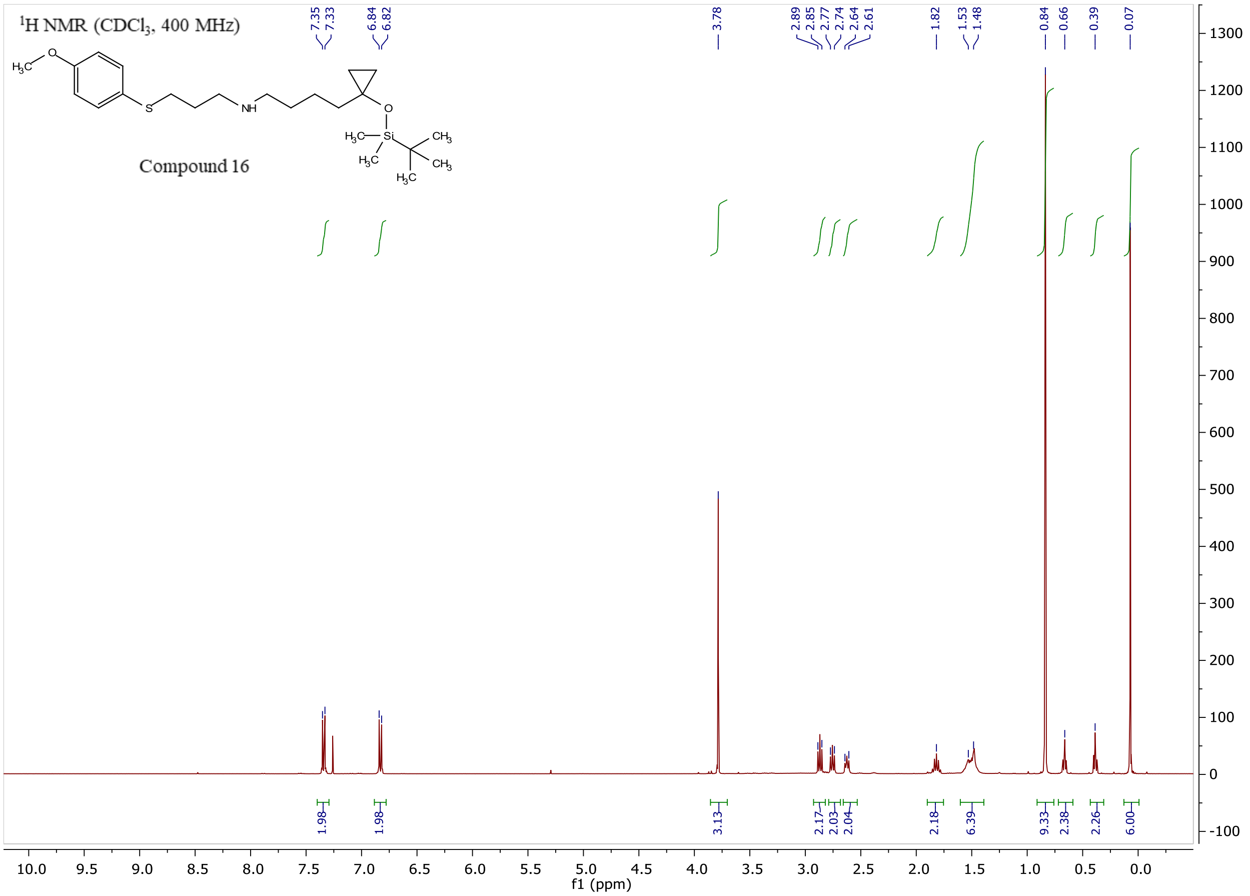

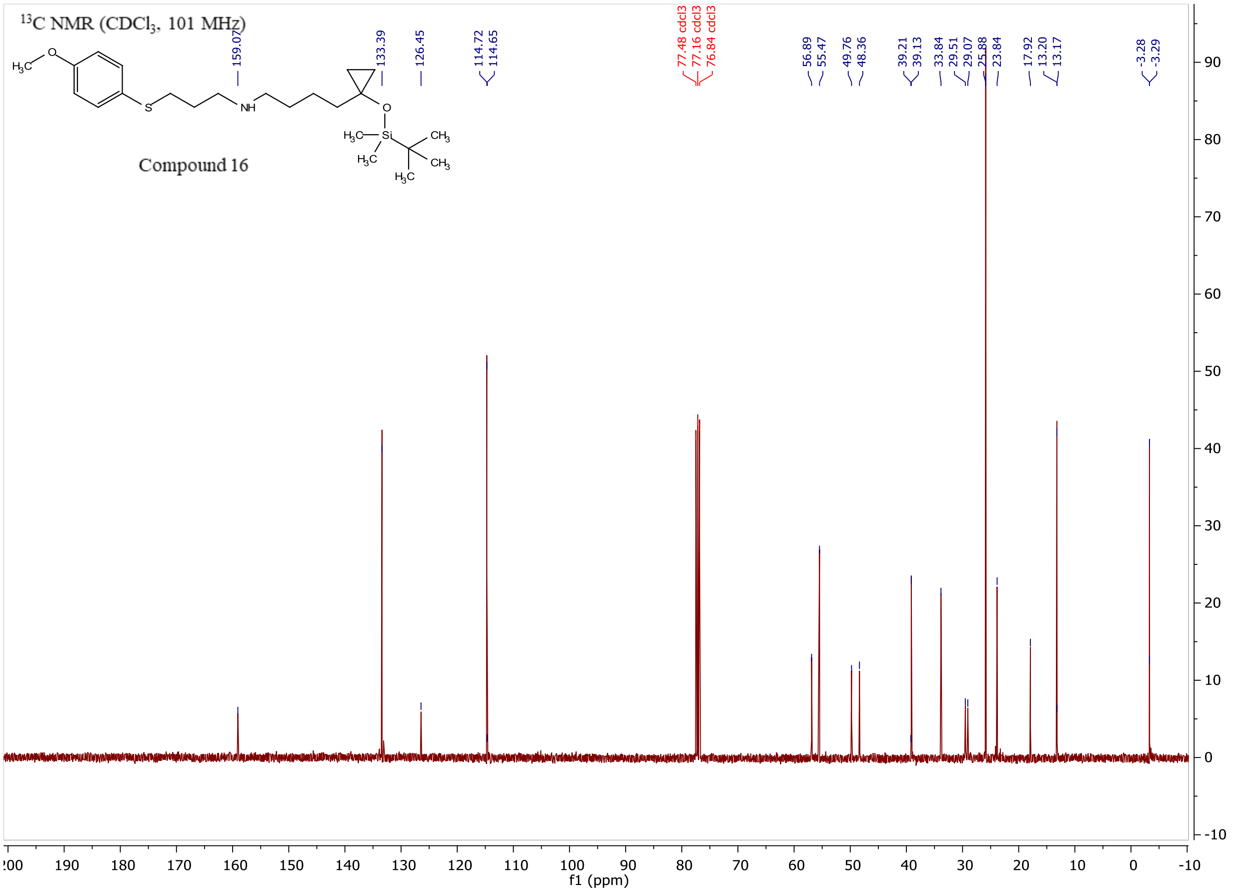

Figure S21. ¹H and ¹³C spectra of compound 16.

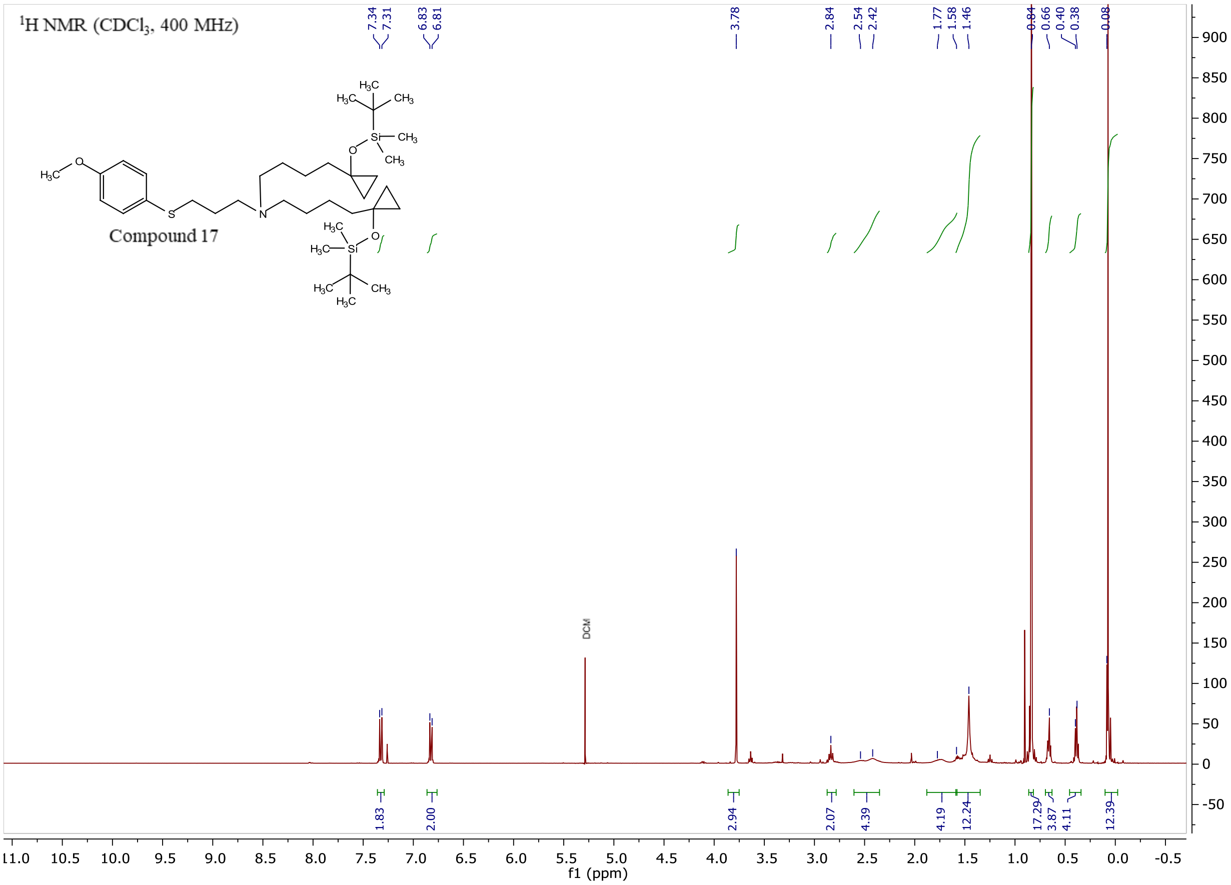

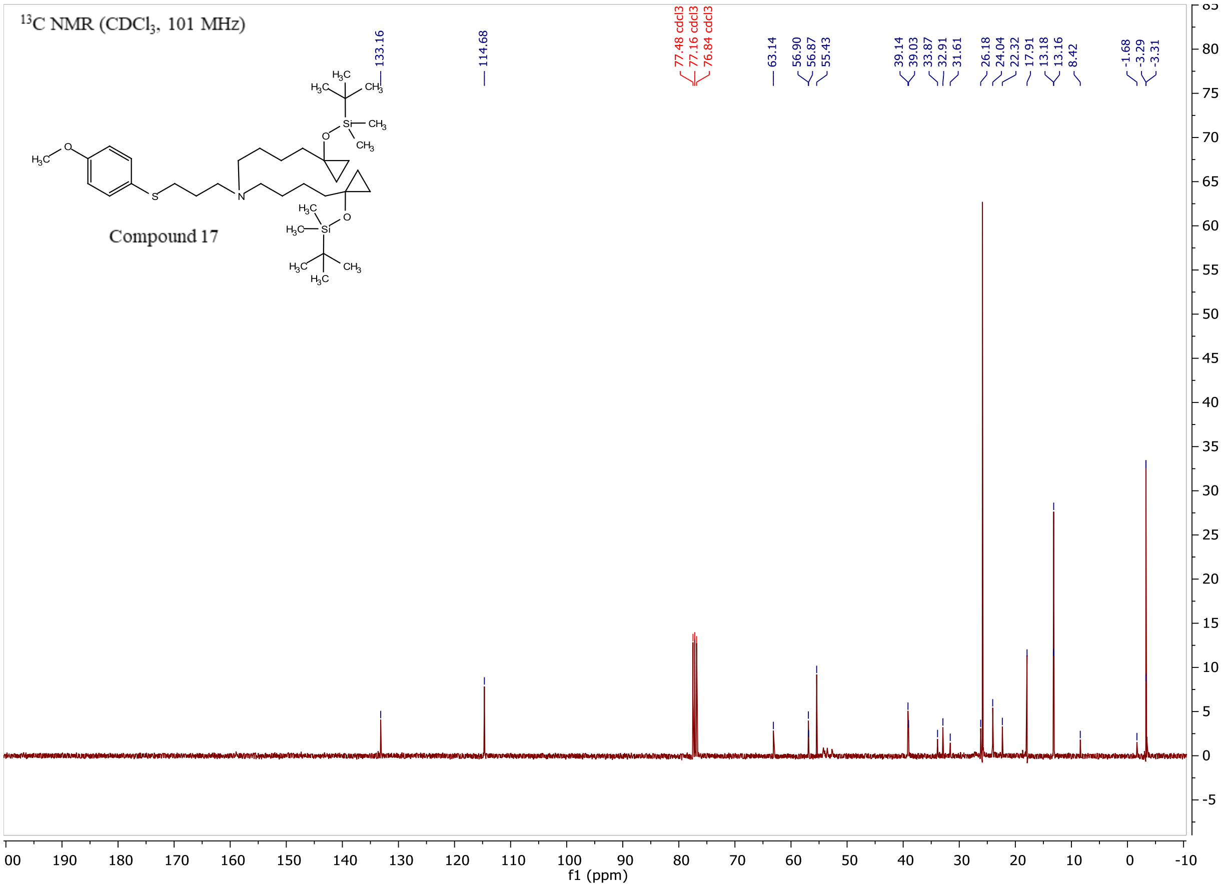

Figure S22. ^{1}H and ^{13}C spectra of compound 17.

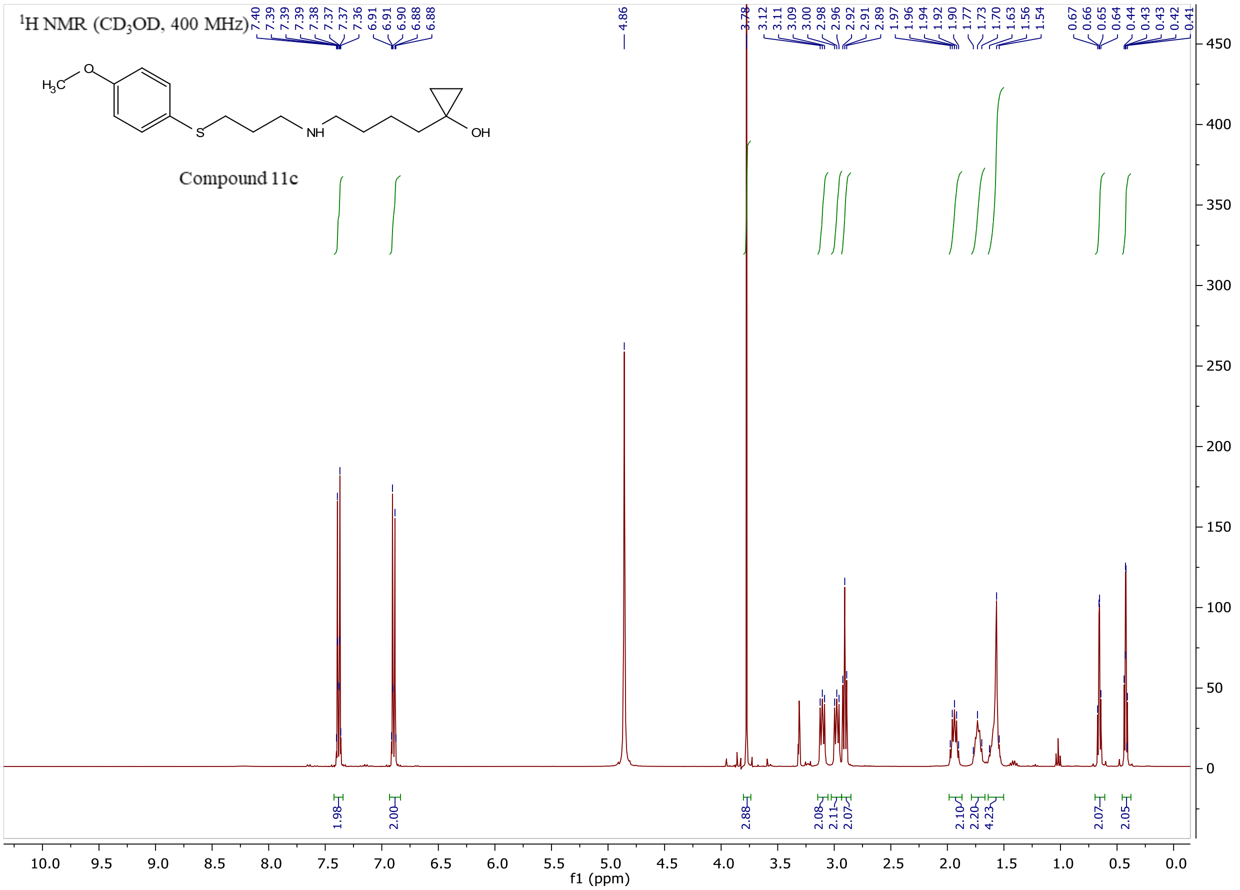

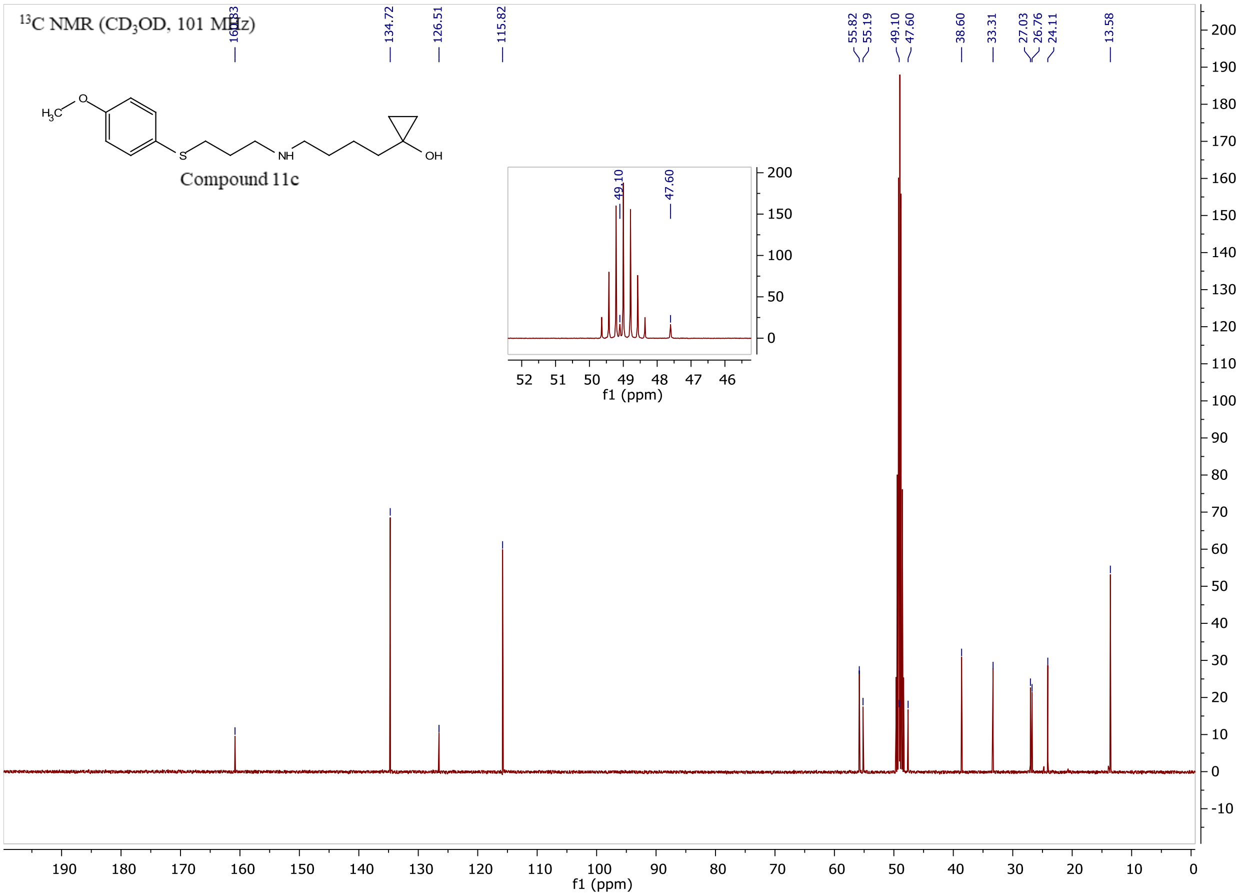

Figure S23. ^1H and ^{13}C spectra of compound 11c.

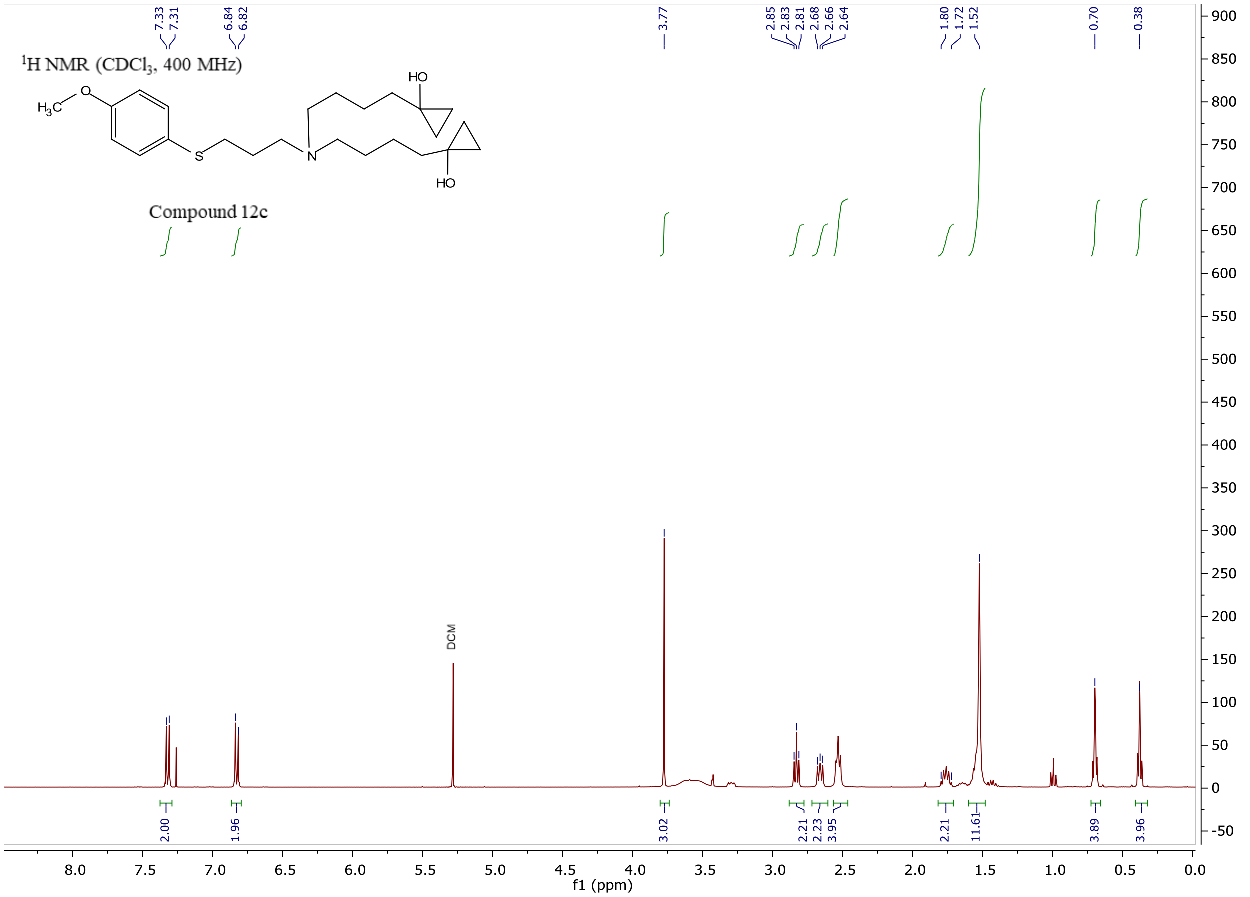

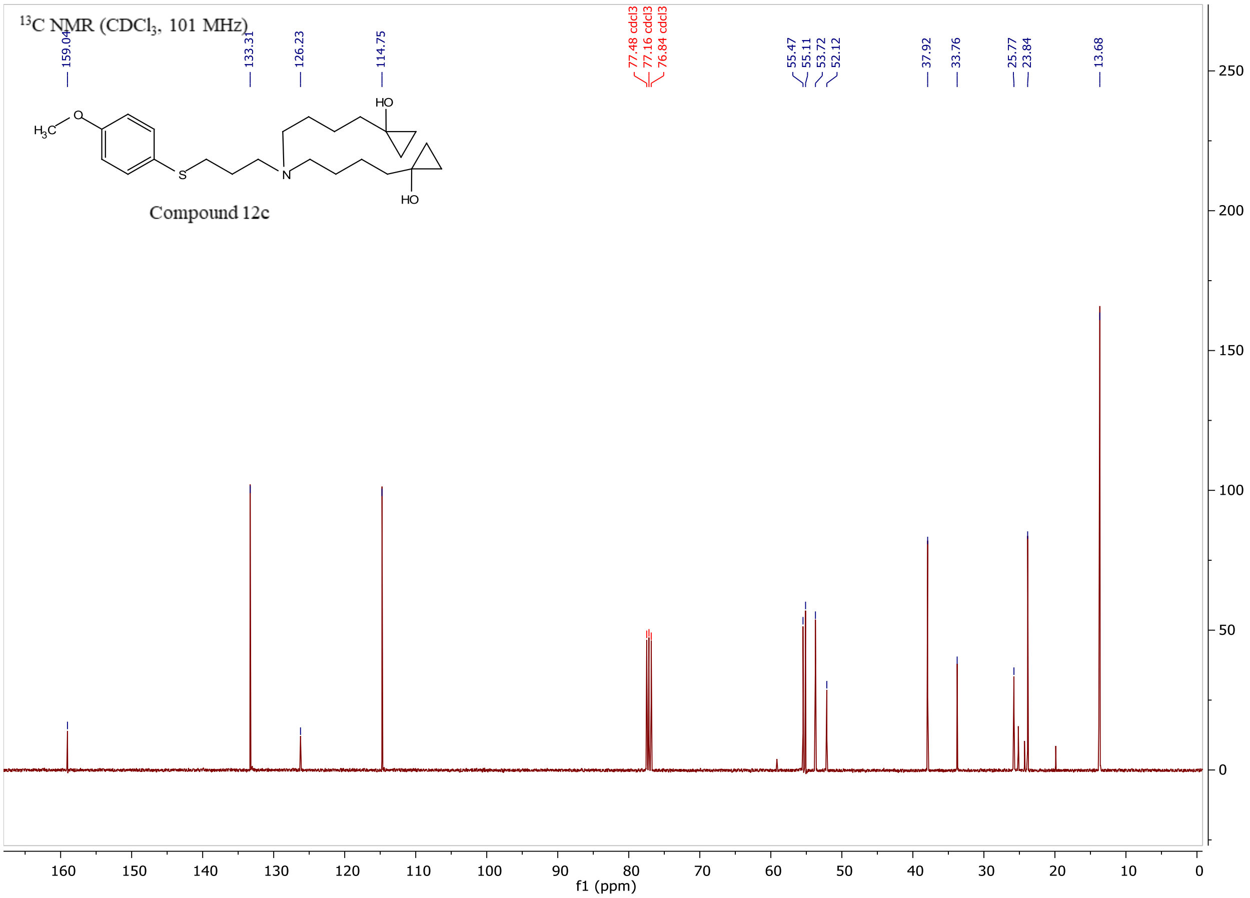

Figure S24. ^{1}H and ^{13}C spectra of compound 12c.

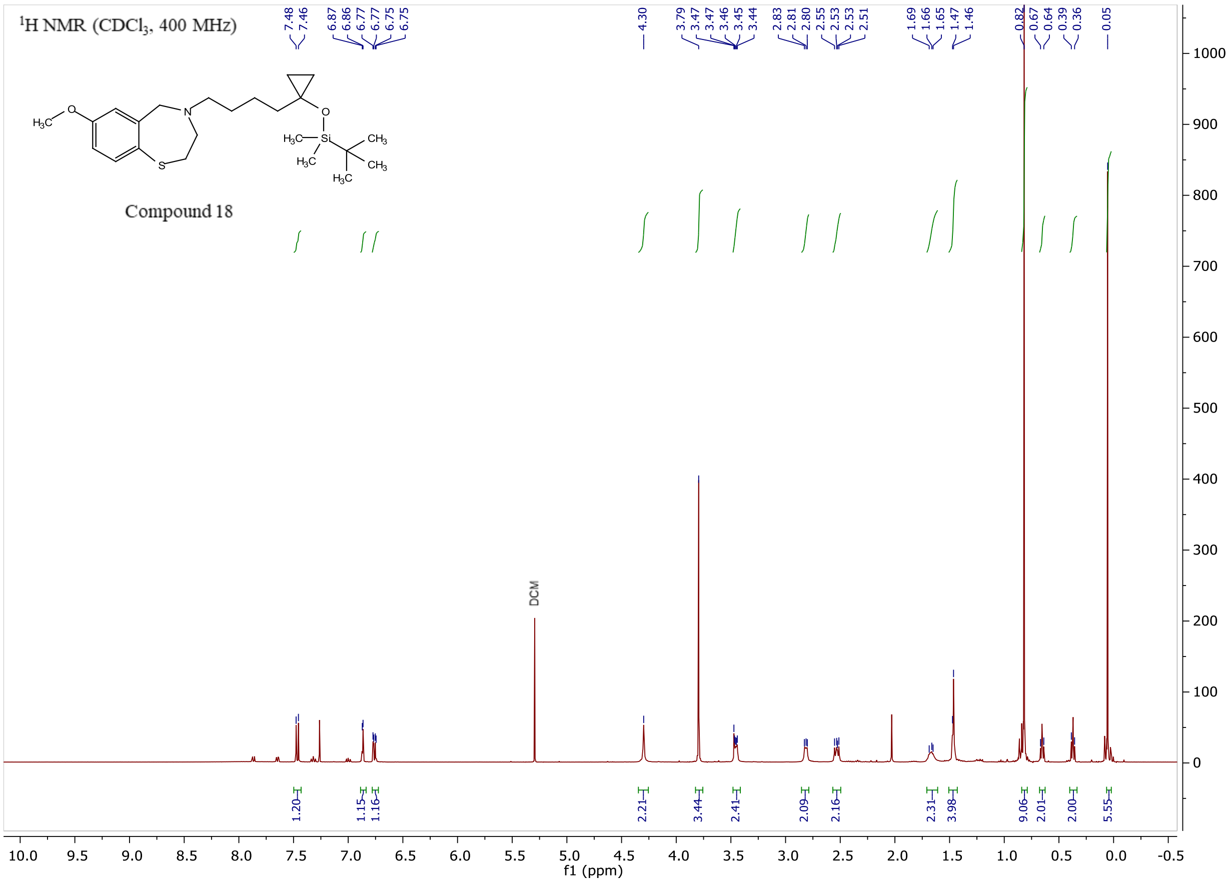

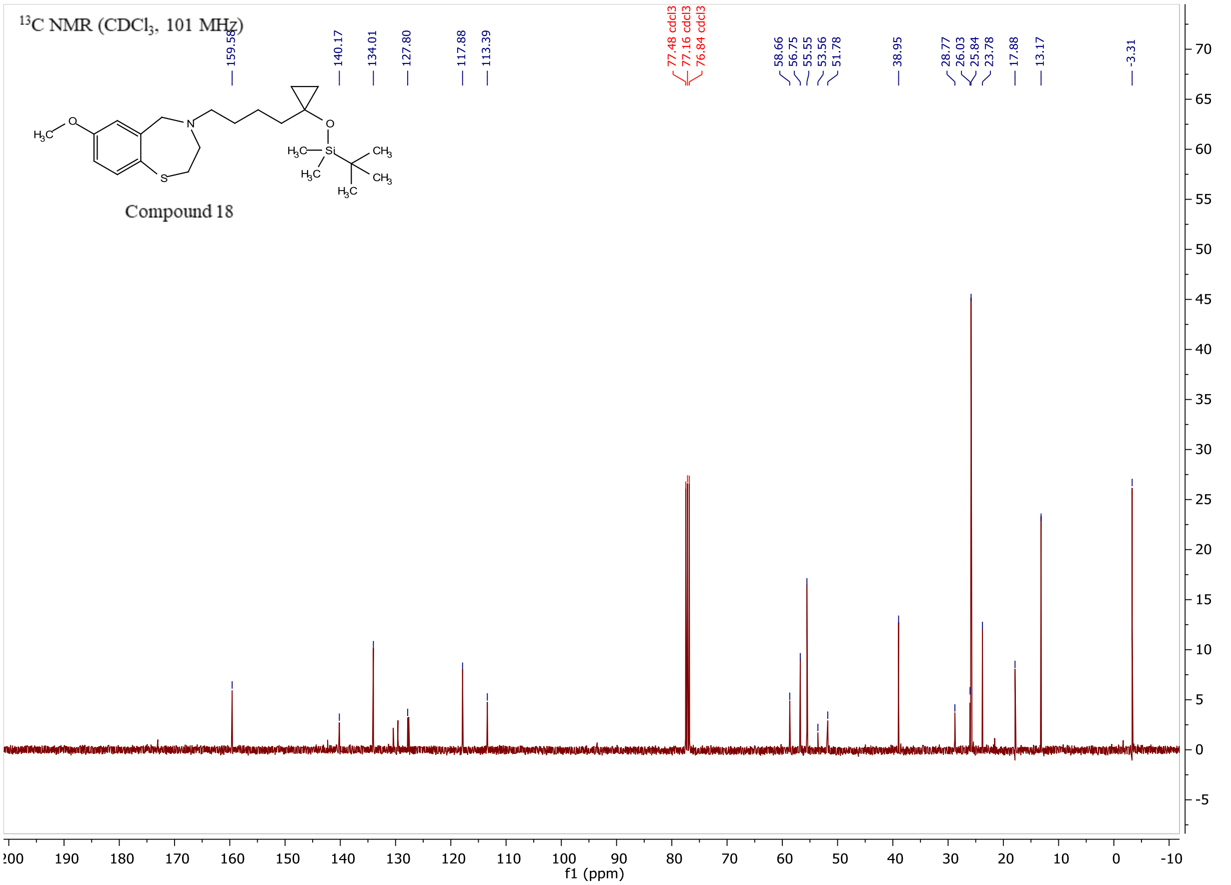

Figure S25. ¹H and ¹³C spectra of compound 18.

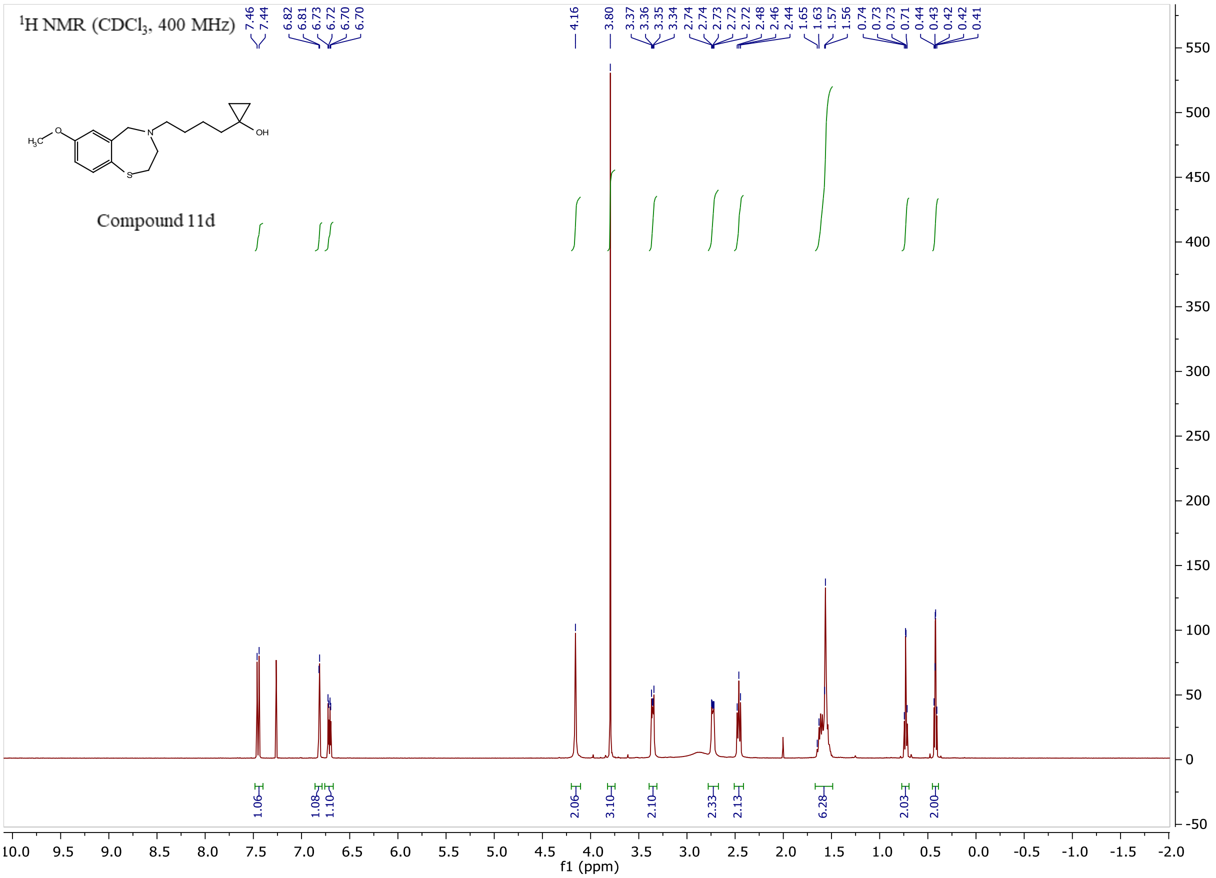

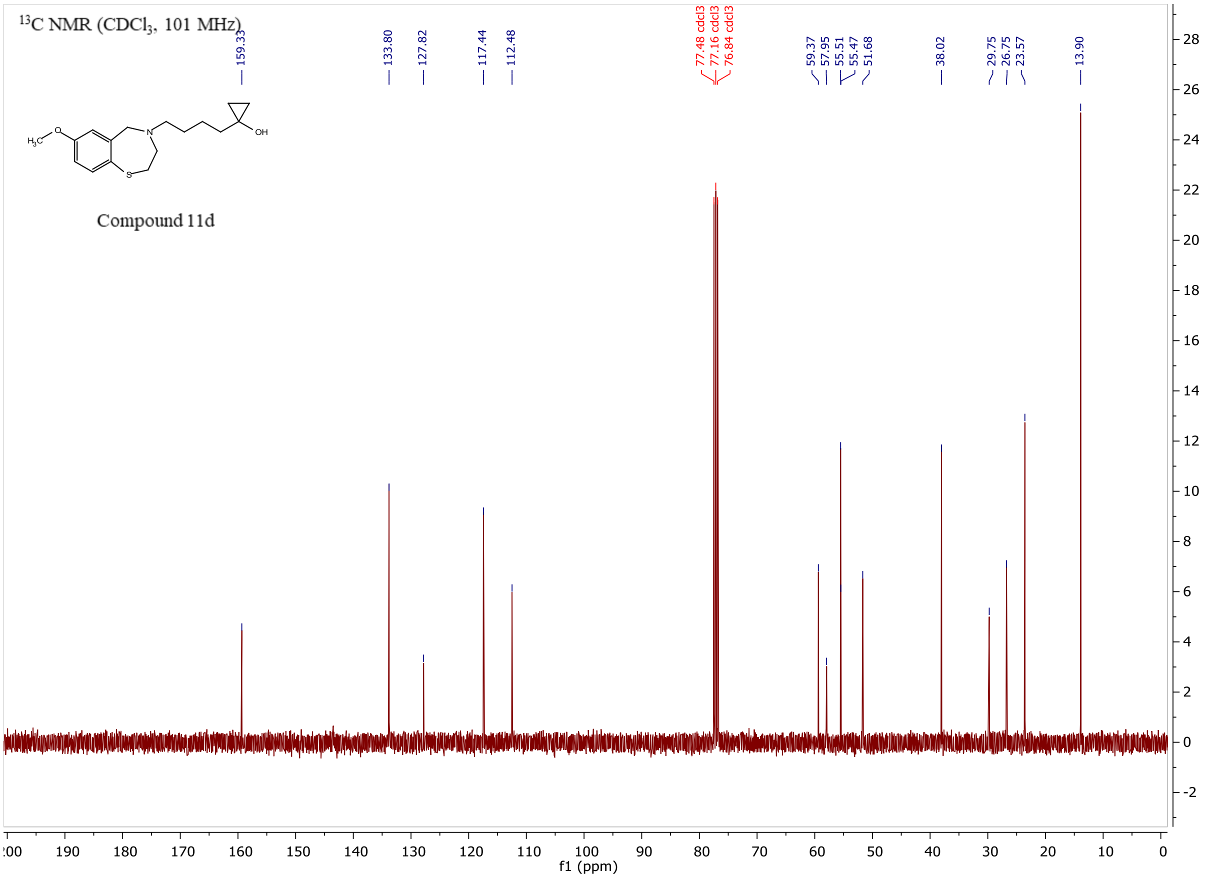

Figure S26. ¹H and ¹³C spectra of compound 11d.

HPLC TRACES

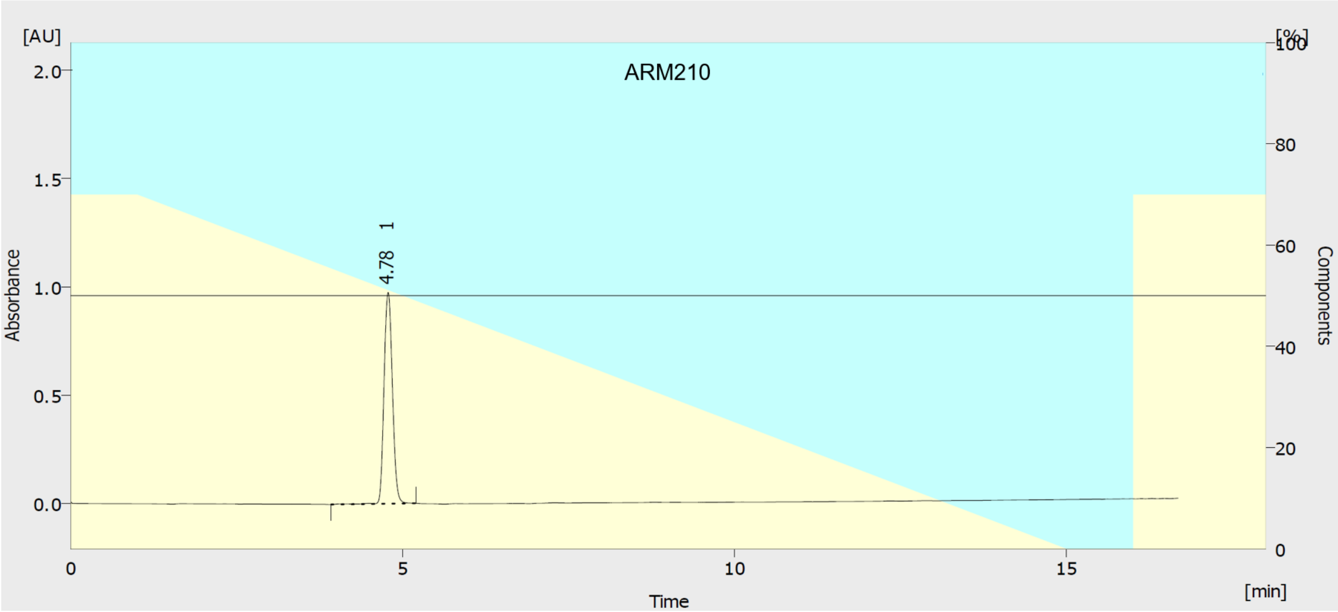

Figure S27. HPLC trace of ARM210. t_R = 4.8 min, 100 % (B/A: 20/80 → 100/0 in 14 min, flow 1.2 ml/min, 254 nm).

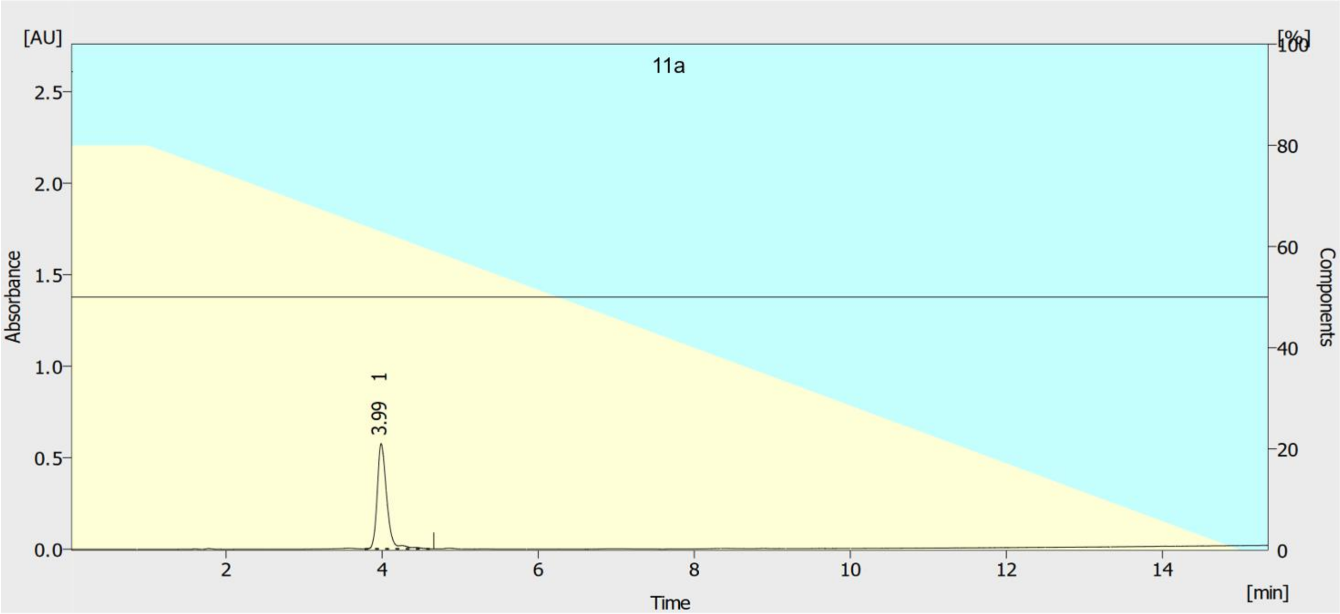

Figure S28. HPLC trace of compound 11a. t_R = 4.0 min, 100 % (B/A: 20/80 → 100/0 in 14 min, flow 1.2 ml/min, 254 nm).

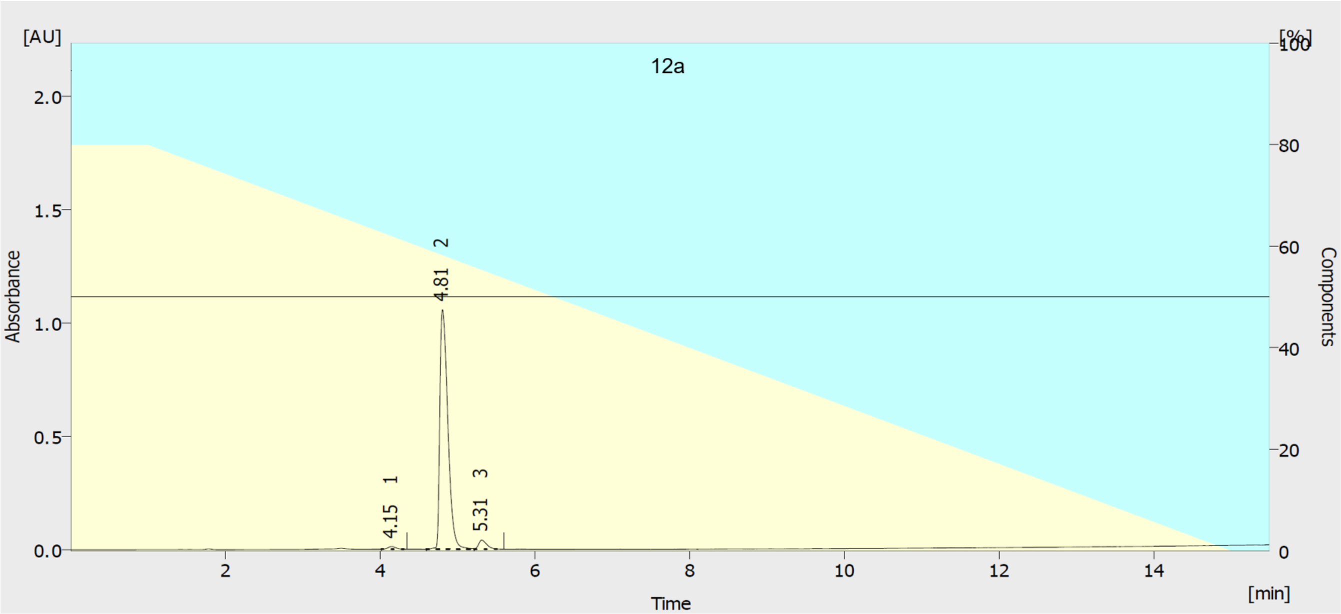

Figure S29. HPLC trace of compound 12a. t_R = 4.8 min, 97% (B/A: 20/80 → 100/0 in 14 min, flow 1.2 ml/min, 254 nm).

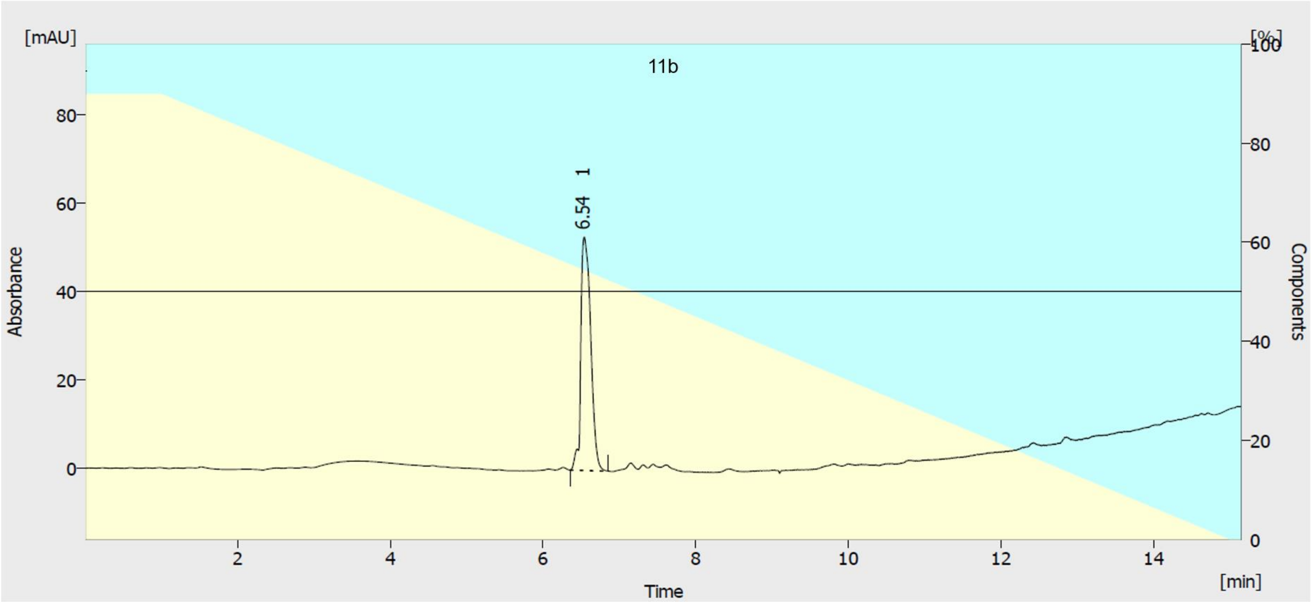

Figure S30. HPLC trace of compound 11b. t_R = 6.5 min, 97% (B/A: 10/90 → 100/0 in 14 min, flow 1.2 ml/min, 254 nm).

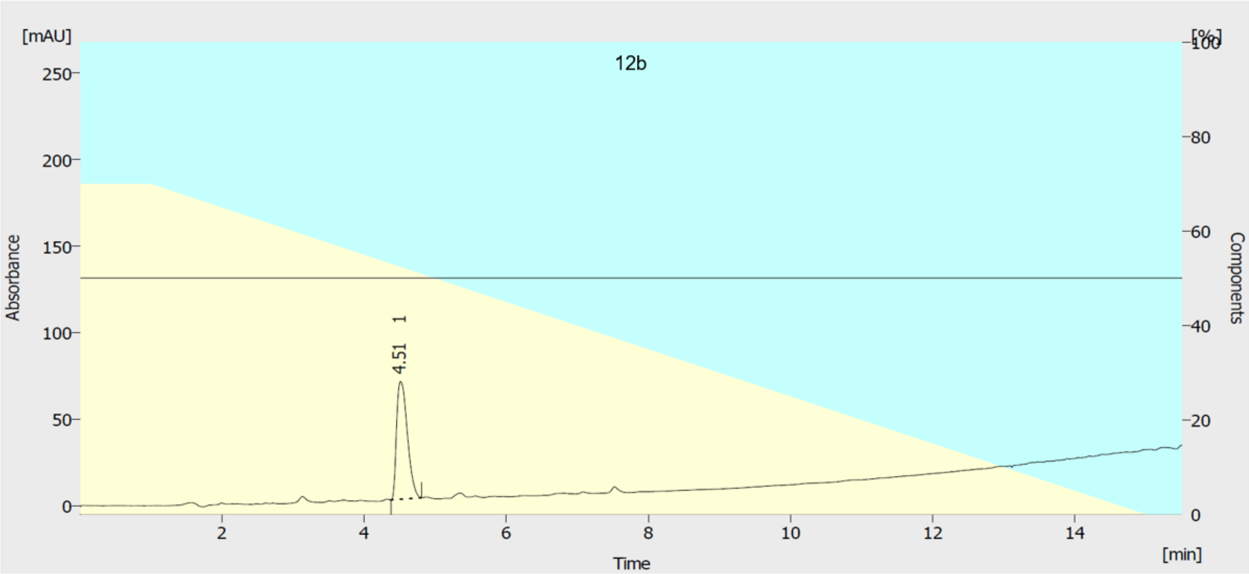

Figure S31. HPLC trace of compound 12b. t_R = 4.5 min, 100 % (B/A: 30/70 – 100/0 in 14 min, flow 1.2 ml/min, 254 nm).

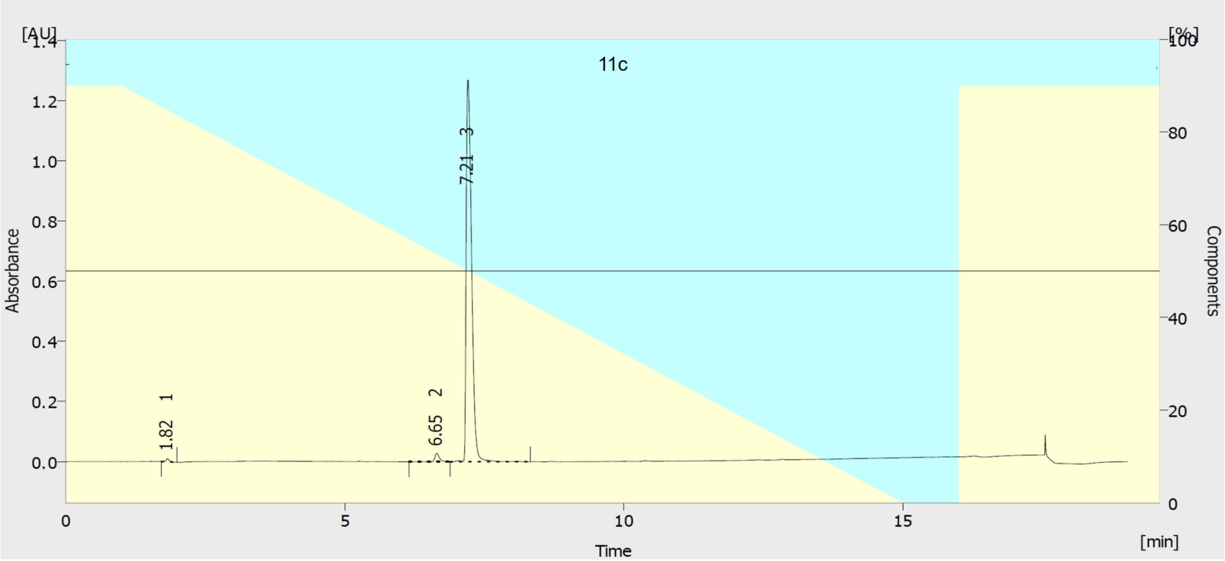

Figure S32. HPLC trace of compound 11c. t_R = 7.2 min, 98 % (B/A: 10/90 – 100/0 in 14 min, flow 1.2 ml/min, 254 nm).

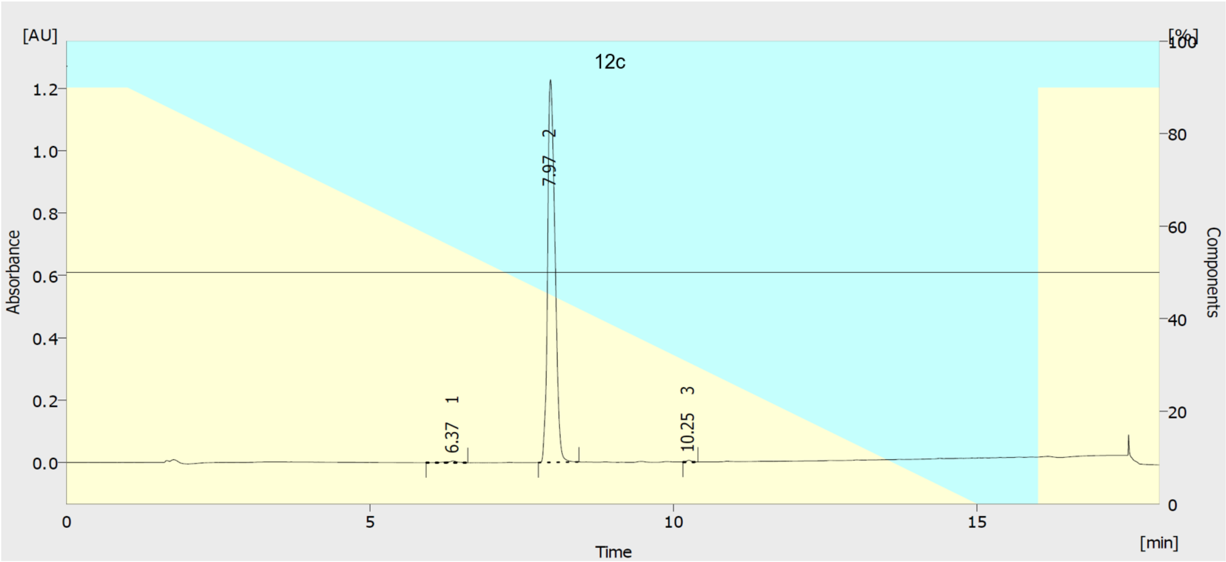

Figure S33. HPLC trace of compound 12c. t_R = 8.0 min, 99 % (B/A: 10/90 – 100/0 in 14 min, flow 1.2 ml/min, 254 nm).

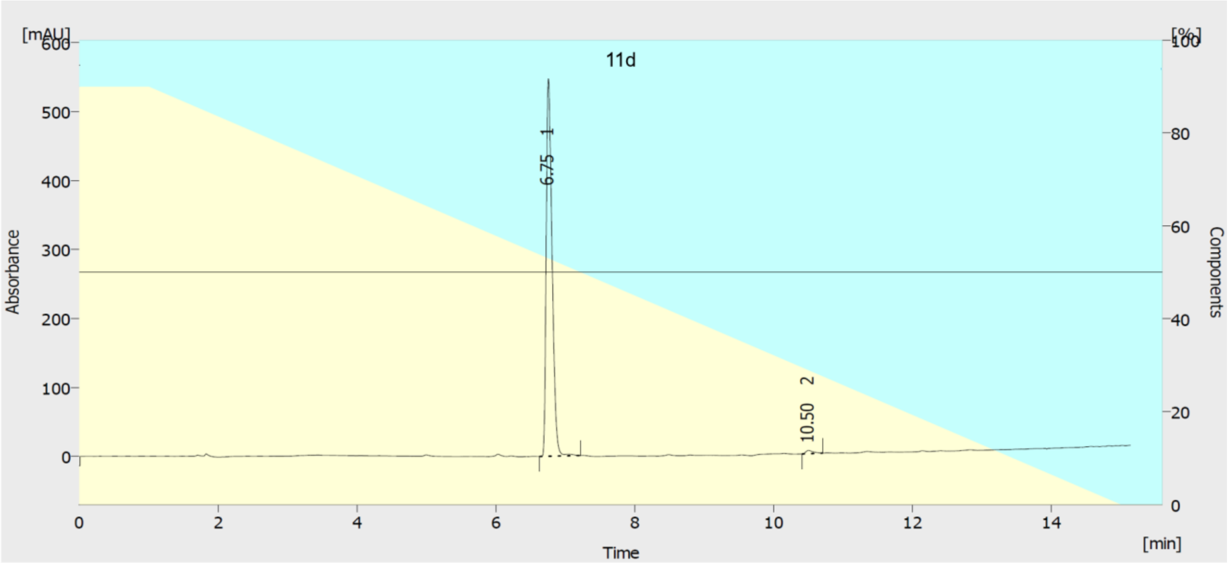

Figure S34. HPLC trace of compound 11d. t_R = 6.8 min, 99 % (B/A: 10/90 – 100/0 in 14 min, flow 1.2 ml/min, 254 nm).

LCMS SPECTRA

LCMS Spectra for compound 11a

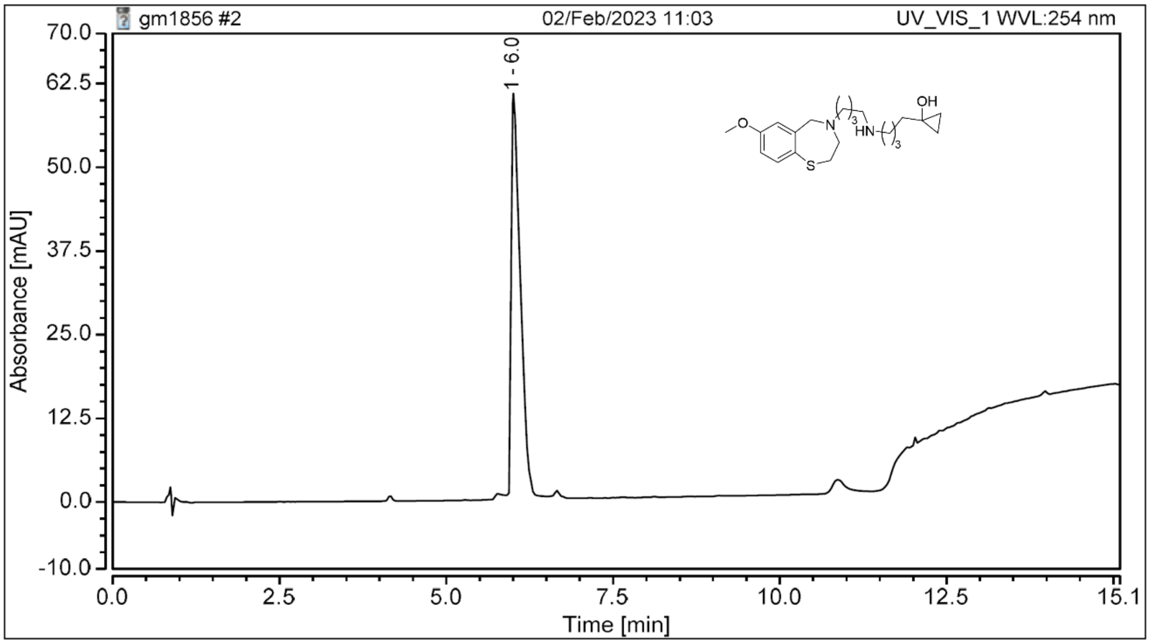

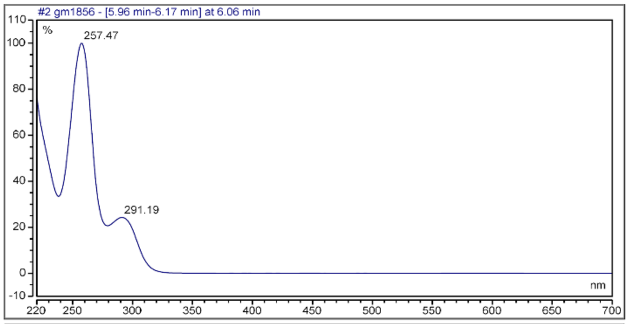

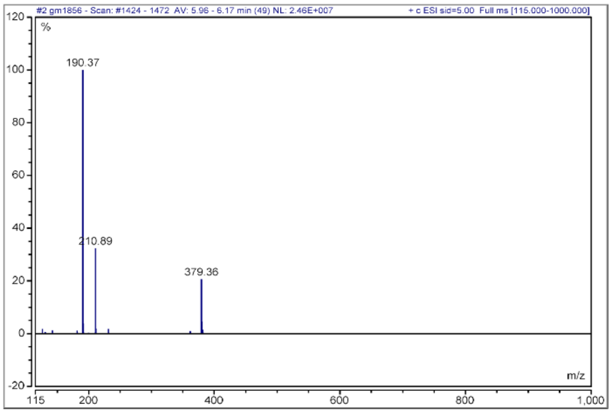

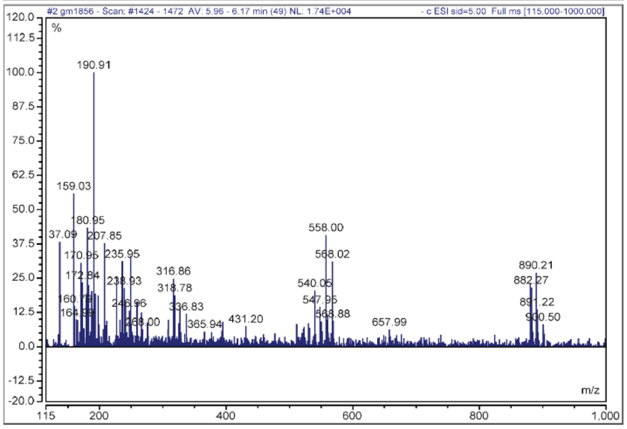

Column: Phenomenex 2.6 µm
Length: 75 mm
iO: 3.0 mm
Flow: 0.5 ml / Min
Temperature : 25.0 °C
Detector: DAD-3000
Pump: HPG-3200SD
Sampler: WPS-3000
Eluent: A = Acetonitrile + 0.1% FA
B = Water + 0.1% FA
Gradient: A 2.0 % B 98.0 % ----> A 30.0 % B 70.0 % in 10 Min.

Figure S35. LCMS spectra of compound 11a.

LCMS Spectra for compound 11b

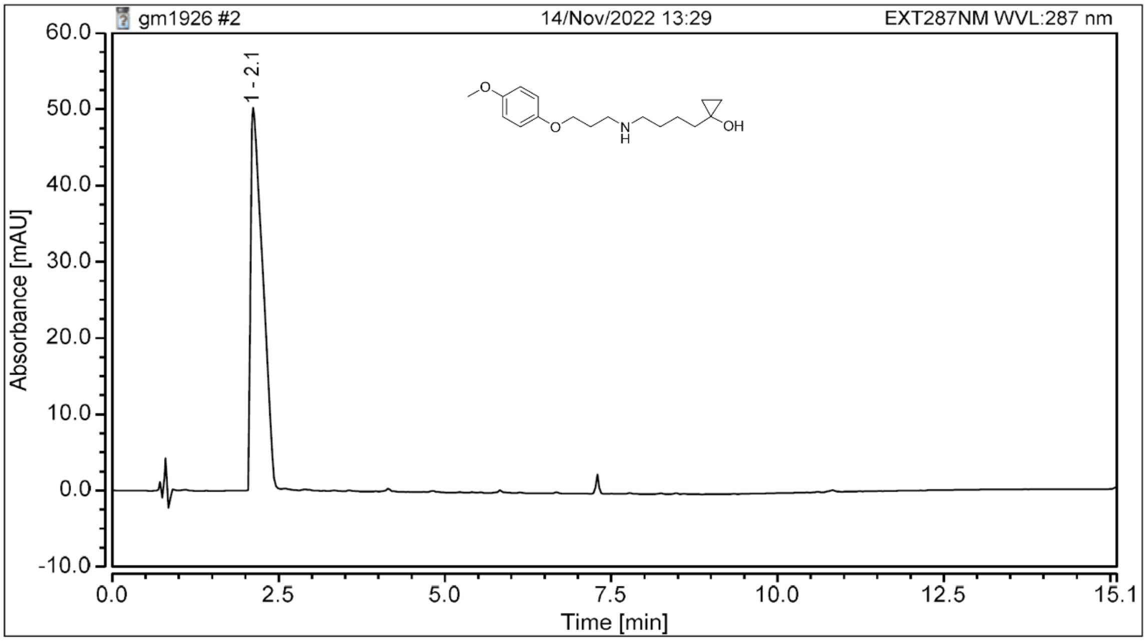

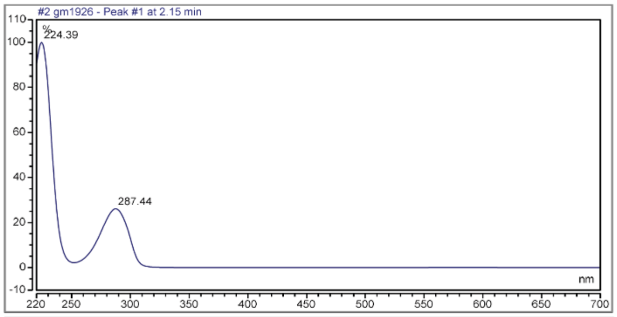

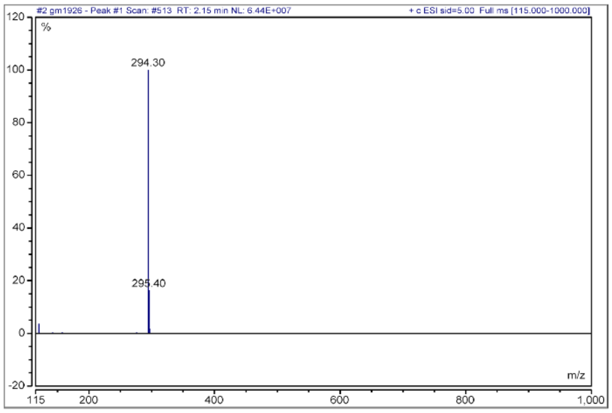

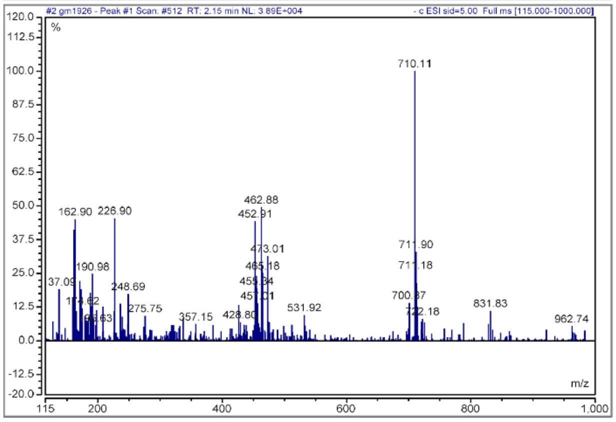

Column: Phenomenex 2.6 µm
Length: 75 mm
iO: 3.0 mm
Flow: 0.5 ml / Min
Temperature : 25.0 °C
Detector: DAD-3000
Pump: HPG-3200SD
Sampler: WPS-3000
Eluent: A = Acetonitrile + 0.1% FA
B = Water + 0.1% FA
Gradient: A 20.0 % B 80.0 % ----> A 100.0 % B 0.0 % in 10 Min.

Figure S36. LCMS spectra of compound 11b.

LCMS Spectra for compound 11c

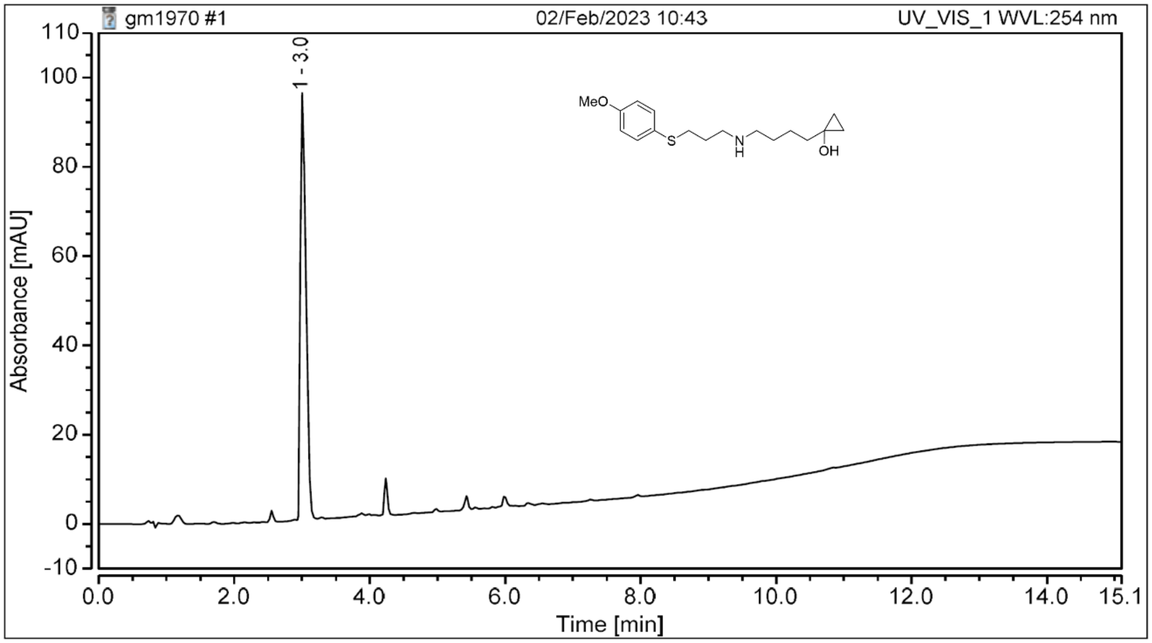

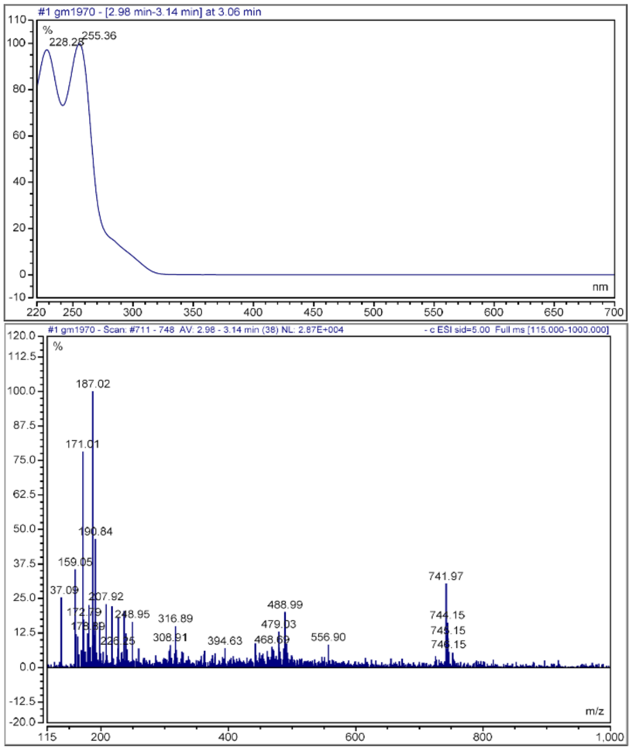

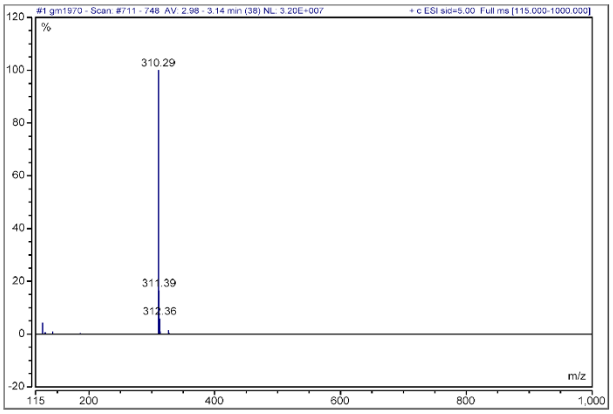

Column: Phenomenex 2.6 µm
Length: 75 mm
iO: 3.0 mm
Flow: 0.5 ml / Min
Temperature : 25.0 °C
Detector: DAD-3000
Pump: HPG-3200SD
Sampler: WPS-3000
Eluent: A = Acetonitrile + 0.1% FA
B = Water + 0.1% FA
Gradient: A 20.0 % B 80.0 % ----> A 100.0 % B 0.0 % in 10 Min.

Figure S37. LCMS spectra of compound 11c.

LCMS Spectra for compound 11d

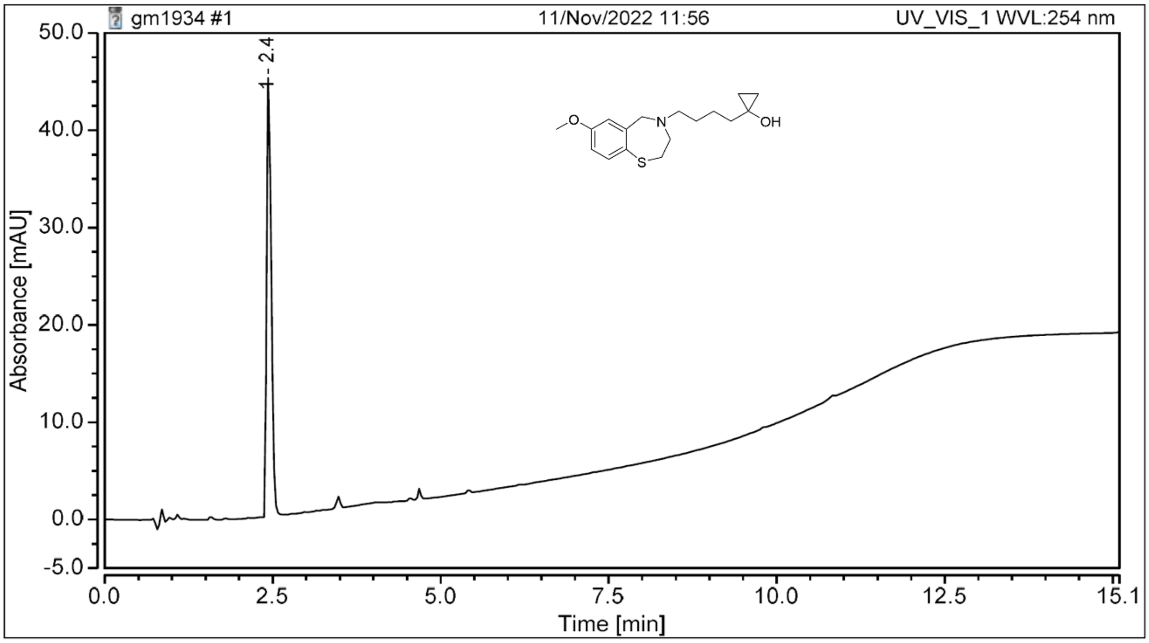

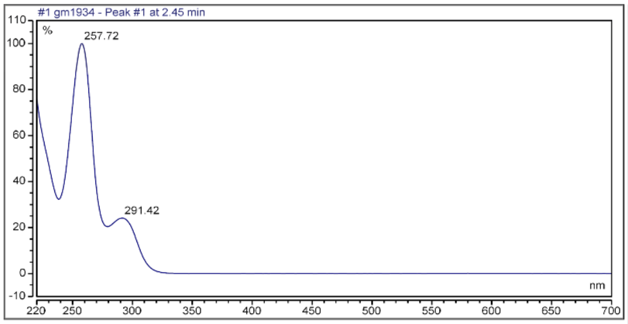

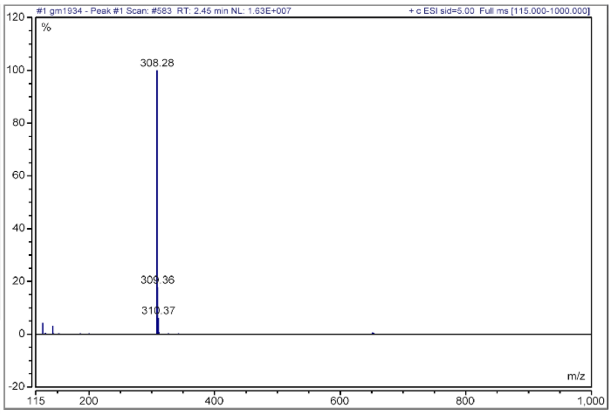

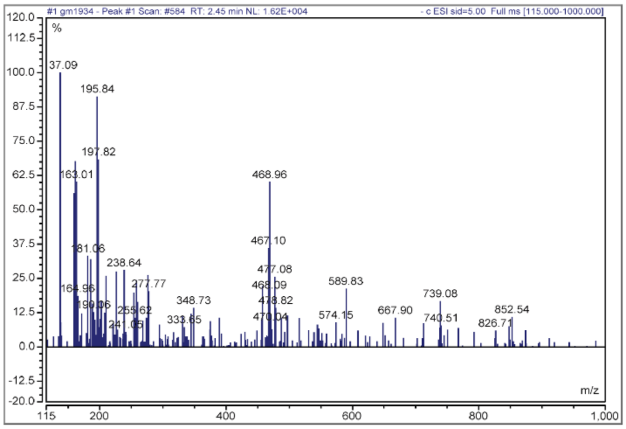

Column: Phenomenex 2.6 µm
Length: 75 mm
iO: 3.0 mm
Flow: 0.5 ml / Min
Temperature : 25.0 °C
Detector: DAD-3000
Pump: HPG-3200SD
Sampler: WPS-3000
Eluent: A = Acetonitrile + 0.1% FA
B = Water + 0.1% FA
Gradient: A 20.0 % B 80.0 % ----> A 100.0 % B 0.0 % in 10 Min.

Figure S38. LCMS spectra of compound 11d.

LCMS Spectra for compound 12a

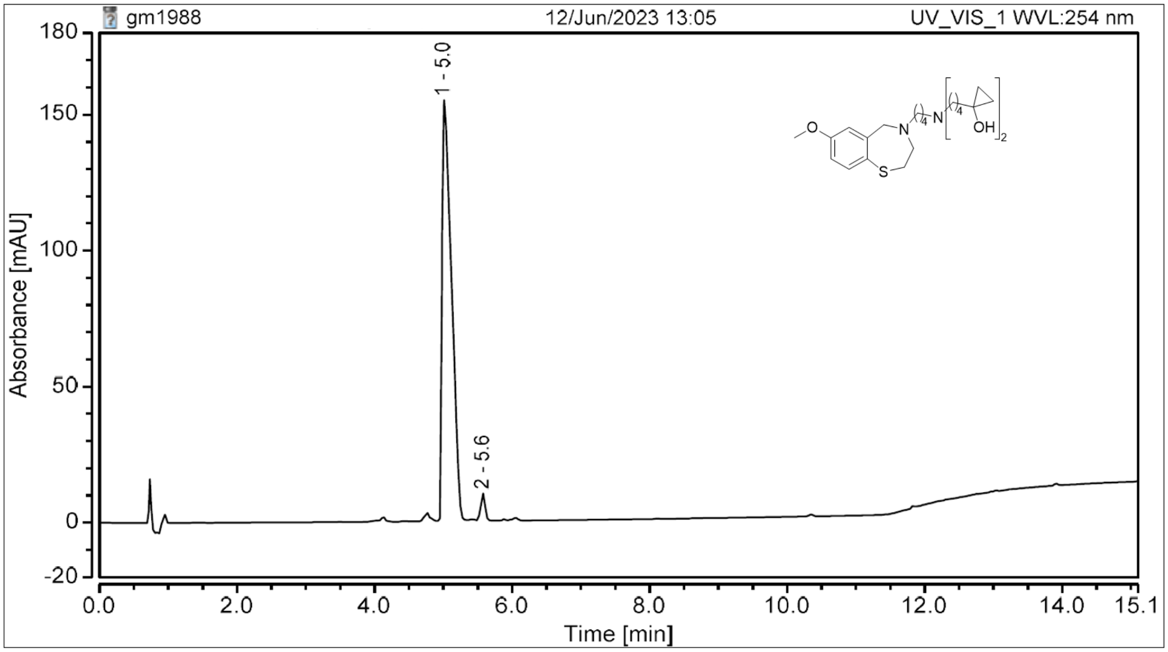

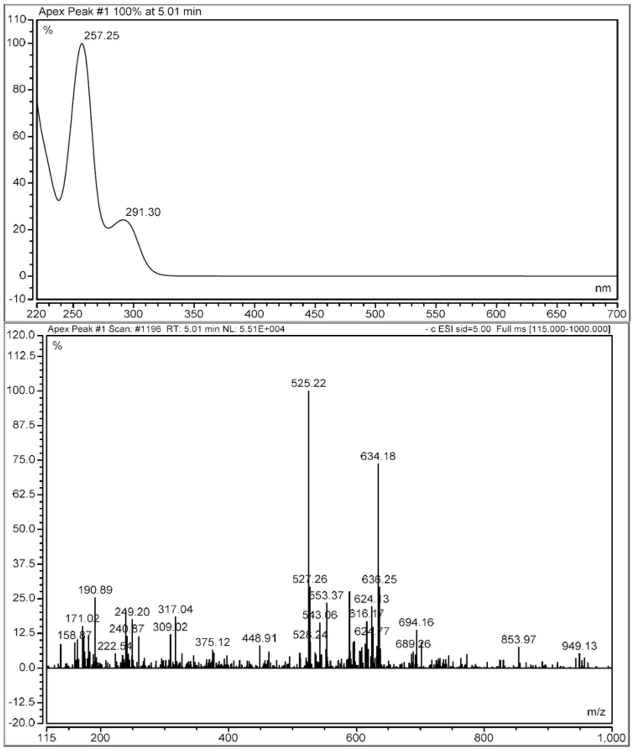

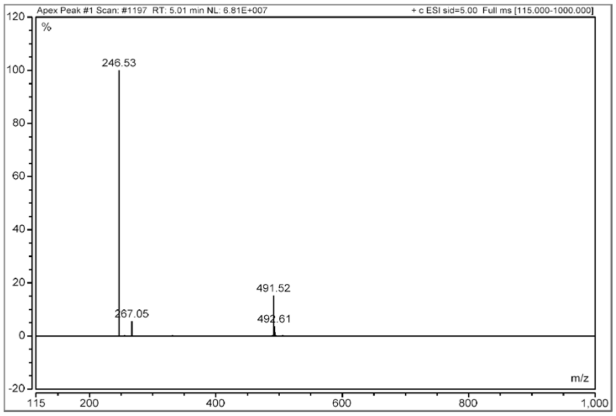

Column: Phenomenex 2.6 µm
Length: 75 mm
iO: 3.0 mm
Flow: 0.5 ml / Min
Temperature : 25.0 °C
Detector: DAD-3000
Pump: HPG-3200SD
Sampler: WPS-3000
Eluent: A = Acetonitrile + 0.1% FA
B = Water + 0.1% FA
Gradient: A 5.0 % B 95.0 % ----> A 50.0 % B 50.0 % in 10 Min.

Figure S39. LCMS spectra of compound 12a.

LCMS Spectra for compound 12c

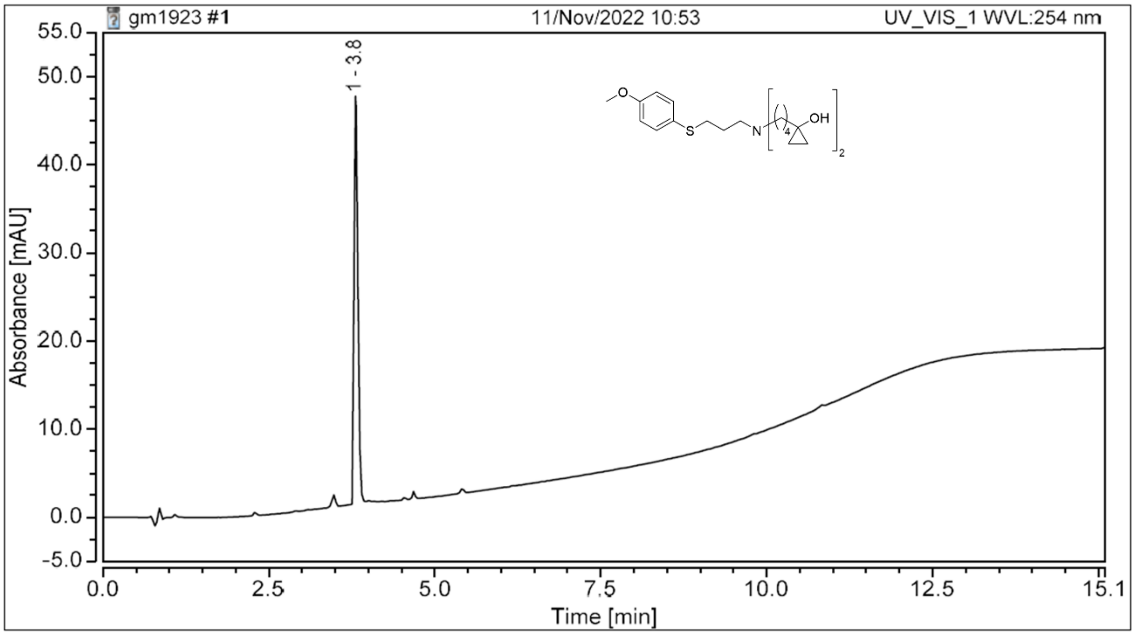

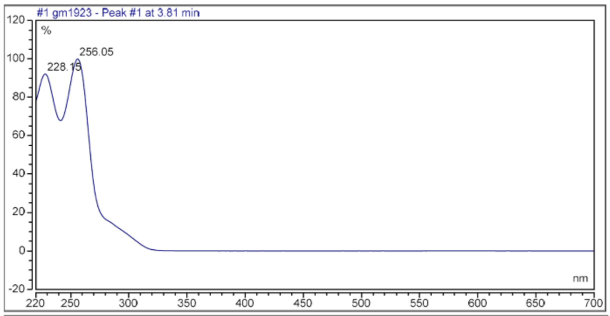

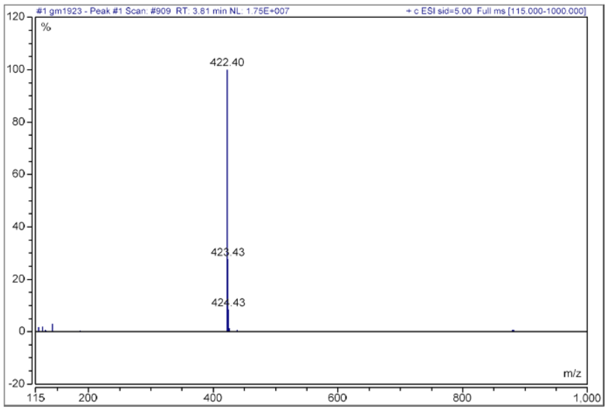

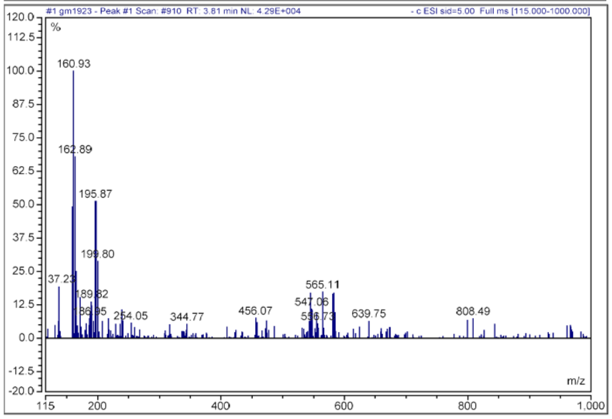

Column: Phenomenex 2.6 µm
Length: 75 mm
iO: 3.0 mm
Flow: 0.5 ml / Min
Temperature : 25.0 °C
Detector: DAD-3000
Pump: HPG-3200SD
Sampler: WPS-3000
Eluent: A = Acetonitrile + 0.1% FA
B = Water + 0.1% FA
Gradient: A 20.0 % B 80.0 % ----> A 100.0 % B 0.0 % in 10 Min.

Figure S40. LCMS spectra of compound 12c.

Notes

PDB 7BT2[1] was used to represent SERCA2a in the TOC graphic. RyR2 and SERCA2a were visualized using VMD molecular visualization program.[2]

ABBREVIATIONS

CAMKII, calcium/calmodulin-dependent kinase; DMEM, Dulbecco's modified eagle medium; ER, endoplasmic reticulum; EtMgBr, ethylmagnesium bromide; FKBP12.6, peptidyl-propyl-cis-trans isomerase calstabin2; HBSS, Hanks' balanced salt solution; HEK-293, human embryonic kidney 293 cells; HEPES, 4-(2-hydroxyethyl)-1-piperazineethanesulfonic acid; HF, heart failure; HL-1, atrial muscle cells; NCX, sodium-calcium exchanger; PLB, phospholamban; RyR2, ryanodine receptor 2; SERCA2a, sarco/endoplasmic reticulum Ca^{2+}-ATPase 2a; SR, sarcoplasmic reticulum; STED, stimulated emission depletion; TBAF, tetrabutylammonium fluoride; TBDMS, *tert*-butyldimethylsilyl; TSTU, *N,N,N',N'*-tetramethyl-*O*-(*N*-succinimidyl)uroniumtetrafluorborat.

REFERENCES

1. Kabashima, Y.; Ogawa, H.; Nakajima, R.; Toyoshima, C., What ATP binding does to the Ca2+ pump and how nonproductive phosphoryl transfer is prevented in the absence of Ca^{2+}. *PNAS* **2020,** *117* (31), 18448-18458.
2. Humphrey, W. D., A.; Schulten, K., VMD - Visual Molecular Dynamics. *J. Mol. Graph.* **1996,** *14*, 33-38.
